# Supplementary material for: Custom tuning of Rieske oxygenase reactivity
Source: Nat Commun. 2023 Sep 20;14:5858. doi: 10.1038/s41467-023-41428-x (PMC10511449; doi:10.1038/s41467-023-41428-x)
Supplement: Supplementary file 1 — Supplementary Information [file 41467_2023_41428_MOESM1_ESM.pdf]

Supplementary Information

Custom Tuning of Rieske Oxygenase Reactivity

Jiayi Tian, Jianxin Liu, Madison Knapp, Patrick H. Donnan, David G. Boggs, and Jennifer Bridwell-Rabb*

Department of Chemistry, University of Michigan, Ann Arbor, Michigan, 48109

*To whom correspondence should be addressed: jebridwe@umich.edu

Supplementary Table 1. Compounds used in this work. An asterisk indicates the compound is not commercially available and the number corresponds to the designation used in the main text document.

	#	NAME		#	NAME
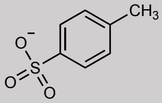	**1**	*p*-toluenesulfonate	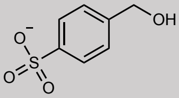	**3**	4-(hydroxymethyl)benzenesulfonate
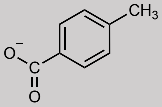	**2**	4-methylbenzoate	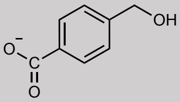	**4**	4-(hydroxymethyl)benzoate
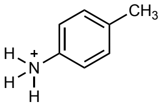	**5**	*p*-aminotoluene	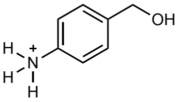	**8**	4-aminobenzyl alcohol
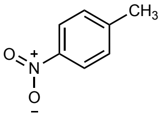	**6**	*p*-nitrotoluene	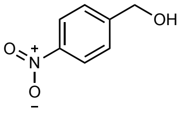	**9**	4-nitrobenzyl alcohol
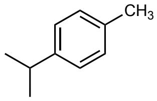	**7**	*p*-isopropyltoluene	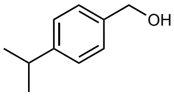	**10**	4-isopropylbenzyl alcohol
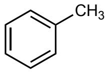	**11**	toluene	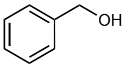	**12**	benzyl alcohol
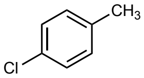	**13**	*p*-chlorotoluene	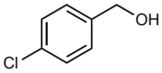	**14**	4-chlorobenzyl alcohol
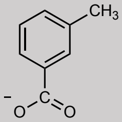	**15**	3-methylbenzoate	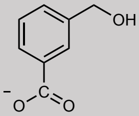	**16**	3-(hydroxymethyl)benzoate
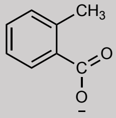	**17**	2-methylbenzoate	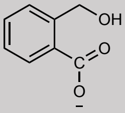	**18**	2-(hydroxymethyl)benzoate
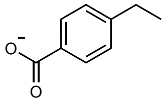	**19**	4-ethylbenzoate	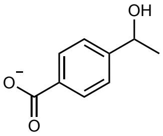	**20**	4-(1-hydroxyethyl)benzoate
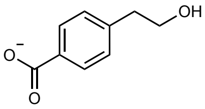	**21**	4-(2-hydroxyethyl)benzoate	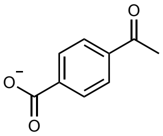	**23**	4-acetylbenzoate
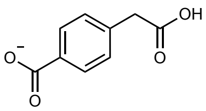	* **22**	4-(carboxymethyl)benzoate	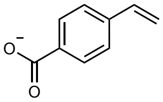	**24**	4-vinylbenzoate
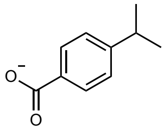	**25**	4-isopropylbenzoate	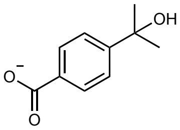	**26**	4-(2-hydroxy-2-propyl) benzoate

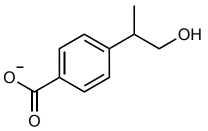	**27** *	4-(1-hydroxypropan-2-yl) benzoate	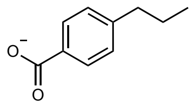	**28**	4-propylbenzoate
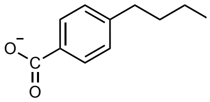	**29**	4-butylbenzoate	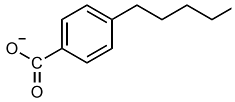	**30**	4-pentylbenzoate
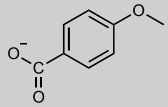	**31**	*p*-(methoxy)benzoate	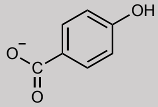	**32**	4-hydroxybenzoate
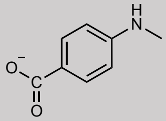	**33**	*p*-(methylamino) benzoate	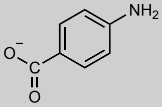	**35**	4-aminobenzoate
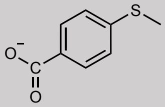	**34**	*p*-(methylthio) benzoate	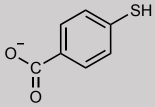	**36**	4-mercapto benzoate
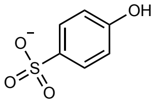	**37**	benzenesulfon ate	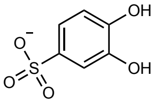	**39**	3,4-dihydroxybenzene sulfonate
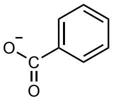	**38**	benzoate	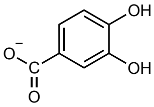	**40**	3,4-dihydroxybenzoate
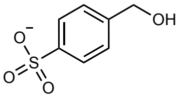	**41**	4-hydroxybenzene-sulfonate	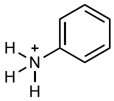	**42**	aniline
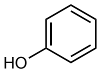	**43**	phenol	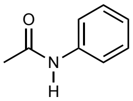	**44**	*N*-phenylacetamide
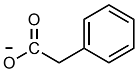	**45**	phenylacetate	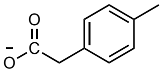	**46**	4-methylphenyl acetate
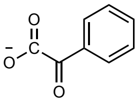	**47**	benzoylformate	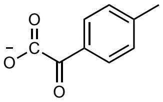	**48**	4-methyl benzoylformate
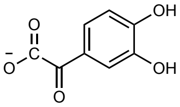	**49** *	3,4-dihydroxybenzoyl formate	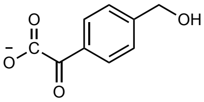	**50** *	4-hydroxymethyl benzoylformate
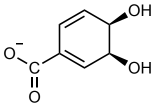	**51** *	(3*R*,4*S*)-3,4-dihydroxycyclohexa-1,5-diene-1-carboxylate	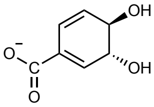	**52**	(3*R*,4*R*)-3,4-dihydroxycyclohexa-1,5-diene-1-carboxylate
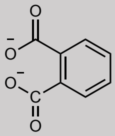	**53**	phthalate	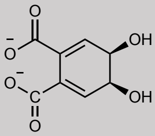	**54** *	(4*R*,5*S*)-4,5-dihydroxycyclohexa-2,6-diene-1,2-dicarboxylic acid

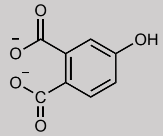	**55**	4-hydroxyphthalate	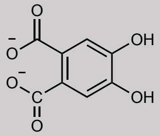	* **56**	3,4-dihydroxyphthalate
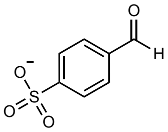	**57**	4-formyl benzenesulfonate	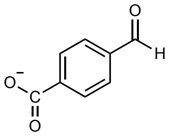	**58**	4-formyl benzoate

Supplementary Table 2. Primers for site-directed mutagenesis used in this work.

**Primer name**	**Primer DNA sequence**
TsaM_M230F	5'-ggcgctgctgcgtttcgataccggcagcg-3'
TsaM_T232F	5'-ggcgctgctgcgtatggatttcggcagcgcg-3'
TsaM_M230W	5'-ccggcgctgctgcgttgggataccggca-3'
TsaM_T232W	5'-ggcgctgctgcgtatggattggggcagcgcgc-3'
TsaM_M230G/T232G	5'-cgccggcgctgctgcgtggggatggcggcagcgc-3'
TsaM_T232I	5'-gctgctgcgtatggatatcggcagcgc-3'
TsaM_S257R	5'-gtttcgtcacaccaggattcaaaccccggag-3'
TsaM_M230W/T232I	5'-cgccggcgctgctgcgttgggatatcggcagcgc-3'
TsaM_Y269V	5'-gaaaccaccagccacgtctggttctgccaggc-3'
PDO_I256G	5'-cggcgactgccctgggtccgcccaacaacc-3'
PDO_I256A	5'-ggcgactgccctggctccgcccaacaacc-3'
PDO_R207V	5'-accgataagacctggttggtaccgtctactgacaaggc-3'
PDO_S182I	5'-cgacagcgcacacatctccagtctgcact-3'
VanA_V232F	5'-ccacgtgctaatcgagttcggtgttgcacatgccg-3'

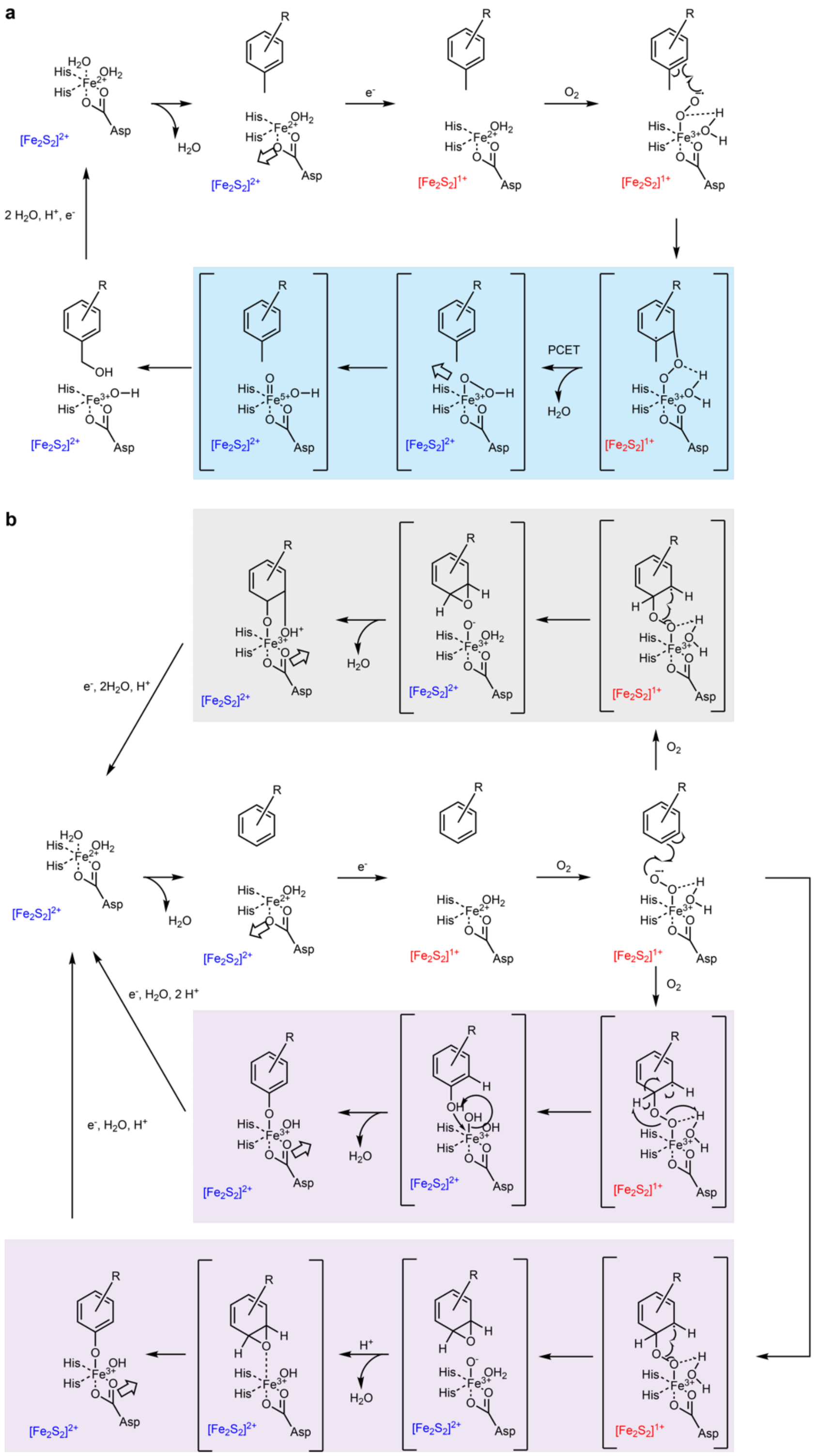

Supplementary Figure 1. Mechanistic proposals for a Rieske non-heme iron dioxygenase and a Rieske non-heme iron monooxygenase[1-3]. (a) Proposed mechanism for methyl hydroxylation by a Rieske oxygenase. Boxed species represent possible intermediates after formation of a common superoxo intermediate, and block arrows indicate conformational shifts of the enzyme[1-3]. (b) Proposed mechanism for mono- and dioxygenation of an aromatic substrate by a Rieske oxygenase. Boxed species represent possible intermediates after formation of a common superoxo intermediate, and block arrows indicate conformational shifts of the enzyme[1-3]. PCET; proton coupled electron transfer.

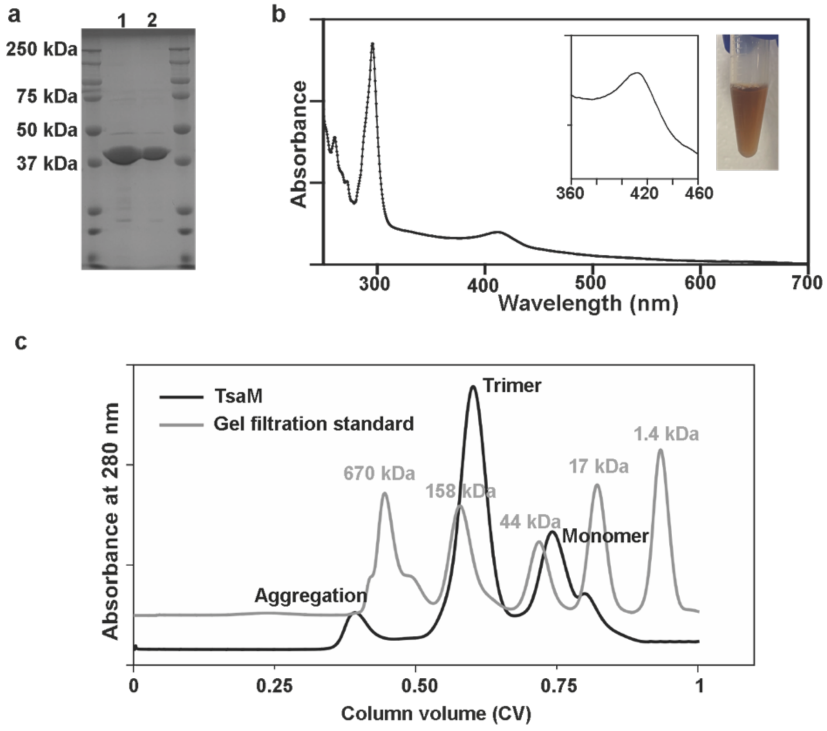

Supplementary Figure 2. The TsaM protein used in this work was recombinantly expressed and purified using affinity and gel filtration chromatography and previously described methods[4]. (a) The purity of TsaM was assessed using SDS-PAGE. TsaM has a molecular weight of approximately 38 kDa and its presence was verified by comparison to a protein standard (unnumbered lanes). (b) A UV-Vis spectrum of purified TsaM shows the characteristic Fe-S cluster absorption peak at 420 nm (see inset). (c) Analysis of the retention time of TsaM on the gel filtration column reveals that the majority of the protein exists in a trimeric or monomeric state, which have molecular weights of approximately 120 and 40 kDa, respectively. This protein was purified and SDS-PAGE assessed approximately 12 times throughout the duration of this work with similar results.

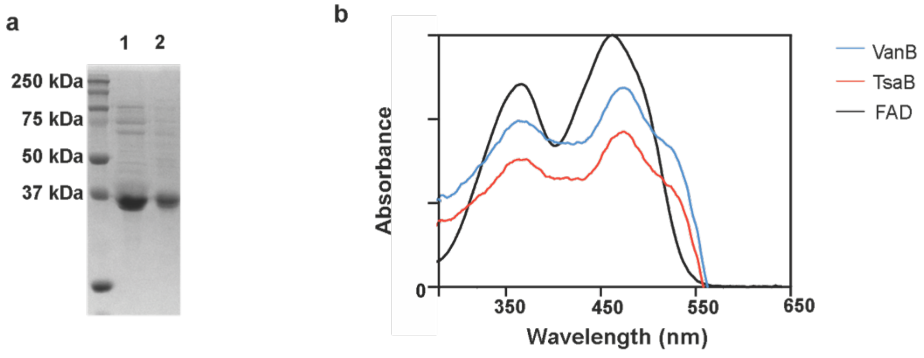

Supplementary Figure 3. The TsaB and VanB reductase proteins used for enzymatic assays in this work were recombinantly expressed and purified as previously described[4]. (a) The purity of TsaB and VanB protein samples was assessed using SDS-PAGE. These proteins have molecular weights of 35 and 36 kDa, respectively. (b) As previously described[4], the characteristic UV-Vis absorbance spectrum of FAD at 450 nm was used to quantify the amount of cofactor incorporated into TsaB and VanB. TsaB purifies with approximately 49-percent FAD bound, whereas VanB is approximately 77-percent bound with FAD. These proteins were purified and SDS-PAGE assessed approximately three and six times for TsaB and VanB, respectively, throughout the duration of this work with similar results.

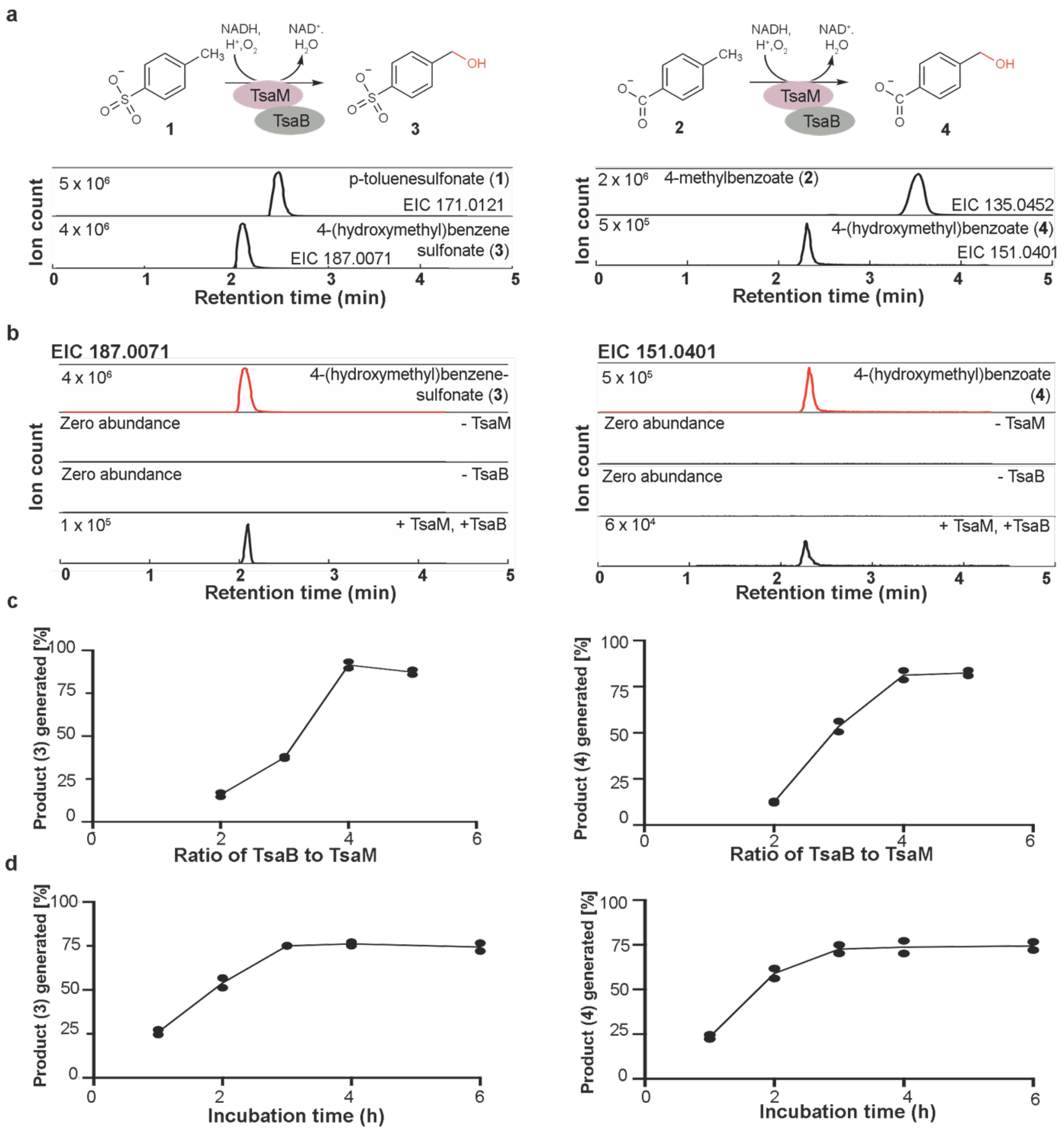

Supplementary Figure 4. The activity of TsaM with the native substrates *p*-toluenesulfonate (**1**) and 4-methylbenzoate (**2**) was tested with the reductase TsaB. (a) To perform this experiment, an LC-MS method[4] that separates the substrates **1** and **2** from the products, 4-(hydroxymethyl)benzenesulfonate (**3**) and 4-(hydroxymethyl)benzoate (**4**) was implemented. Shown here are the extracted ion chromatograms of the substrate and product standards. (b) The activity of TsaM-TsaB on both **1** and **2** was tested using LC-MS. The standard of the expected products, **3** and **4** are shown in red (m/z=187.0071 and m/z=151.0401). As previously indicated[4], these products are only formed when TsaM is combined with TsaB. (c) The extracted ion chromatograms for the TsaM-TsaB reaction products with **1** and **2** reveals that the optimal ratio of TsaB:TsaM trimer is 4:1. (d) The extracted ion chromatograms for the TsaM-TsaB reaction shows that the maximum amount of product is generated after a 3 h incubation. In this figure, the data points in panels c and d were measured using n = 2 independent experiments.

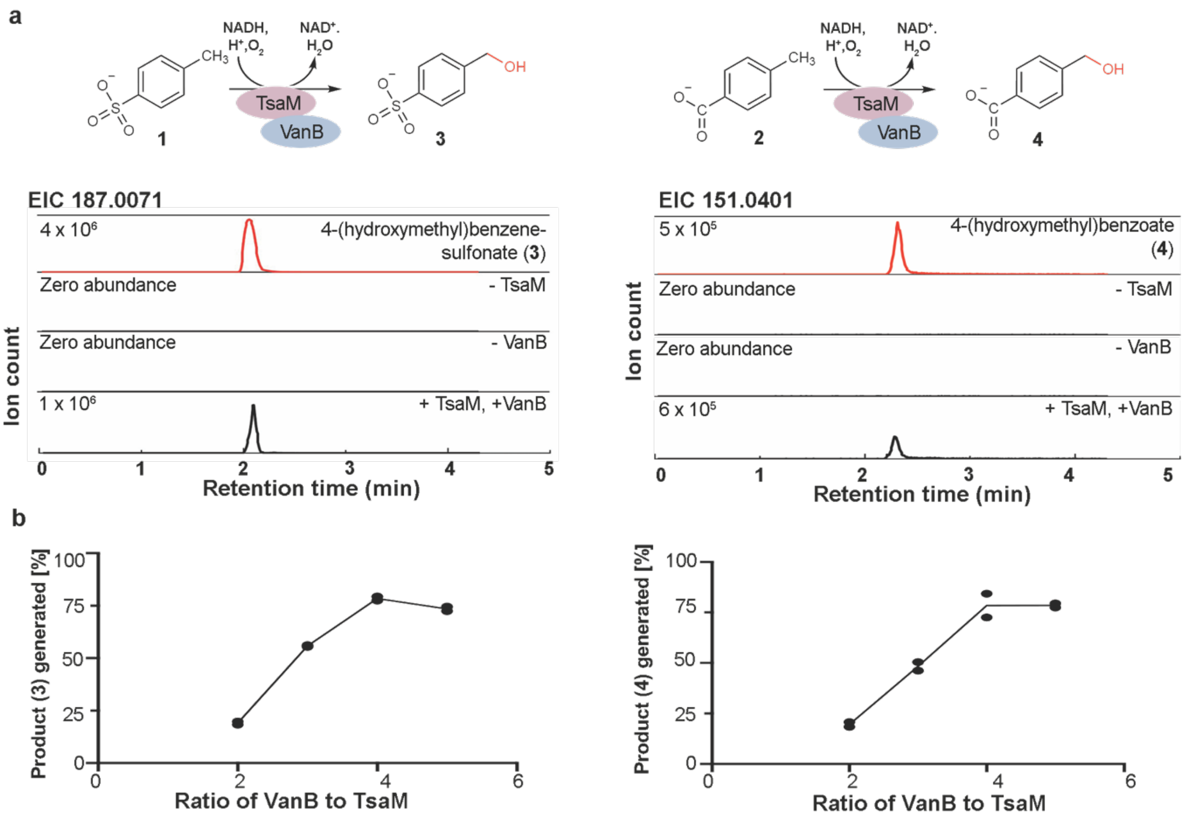

Supplementary Figure 5. The activity of TsaM with the native substrate *p*-toluenesulfonate (**1**) and 4-methylbenzoate (**2**) was tested with the reductase VanB using previously described methods[4]. (a) The standards of the expected products, 4-(hydroxymethyl)benzenesulfonate (**3**) and 4-(hydroxymethyl)benzoate (**4**) are shown in red (m/z=187.0071 and 151.0401). As expected based on prior work[4], these products form when TsaM is combined with VanB. (b) The extracted ion chromatograms for the products formed by the TsaM-VanB system with **1** and **2** reveals that the optimum ratio of VanB:TsaM is 4:1. In this figure, the data points in panel b were measured using n = 2 independent experiments**.**

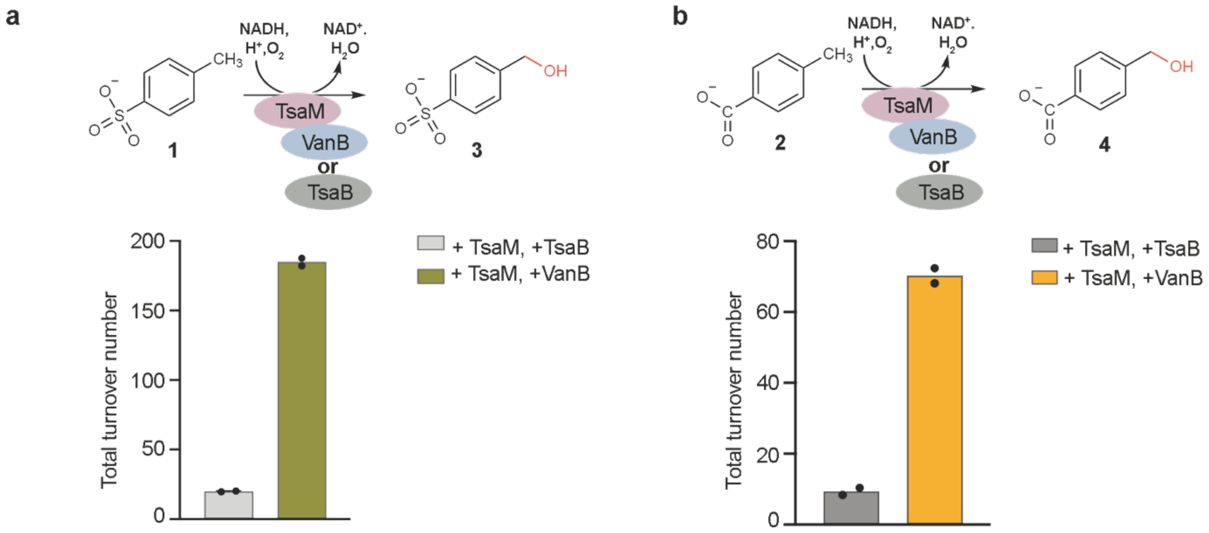

Supplementary Figure 6. A comparison of products generated when the TsaM-TsaB and TsaM-VanB systems are provided with the native substrates indicates that VanB allows for the formation of more monooxygenated product. This reductase trend is consistent with what has been previously determined for TsaM[4]. (a) A comparison of the amount of 4-(hydroxymethyl)benzenesulfonate (**3**) formed using TsaB or VanB as a reductase partner for TsaM reveals that approximately ten times more product is generated when VanB is included in the reaction. (b) Similarly, more 4-(hydroxymethyl)benzoate product is formed using the TsaM-VanB system than the TsaM-TsaB system. In this figure, the data were measured using n = 2 independent experiments**.** Source data are provided as a Source Data file.

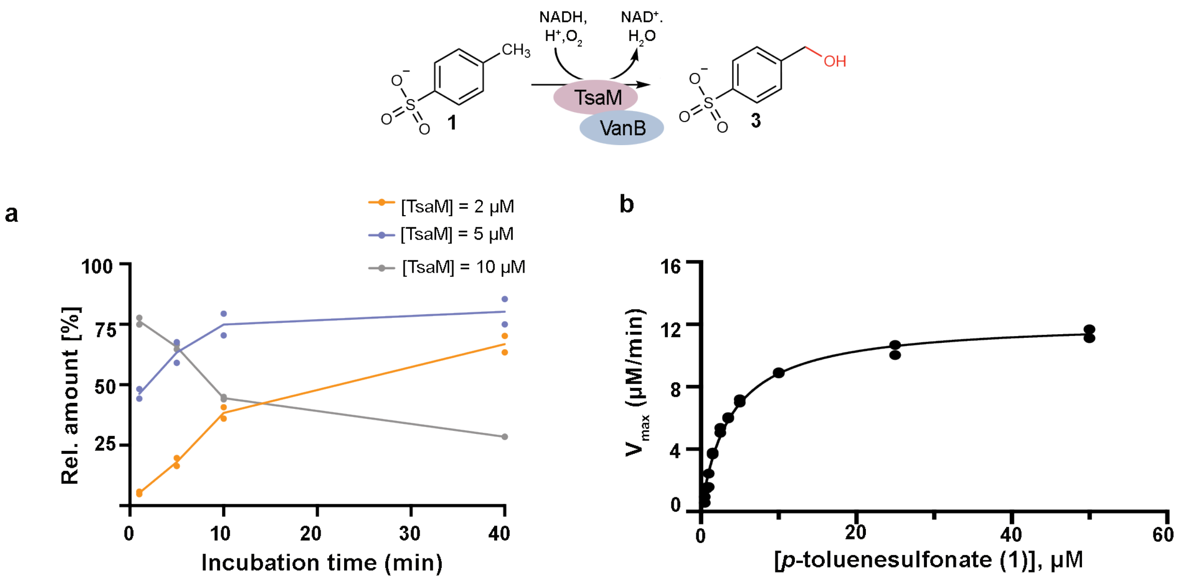

Supplementary Figure 7. The apparent kinetic parameters for the TsaM-VanB system were measured using *p*-toluenesulfonate (**1**) as a substrate. (a) The linear range of 4-(hydroxymethyl)benzenesulfonate (**3**) formation was investigated. A time of 5 min with 2 μM of TsaM was chosen for performing the kinetic assay. (b) Fitting of the data to the Michaelis-Menten equation revealed the kinetic parameters for the reaction (see Table 1). In this figure, the data were measured using n = 2 independent experiments**.** Source data are provided as a Source Data file.

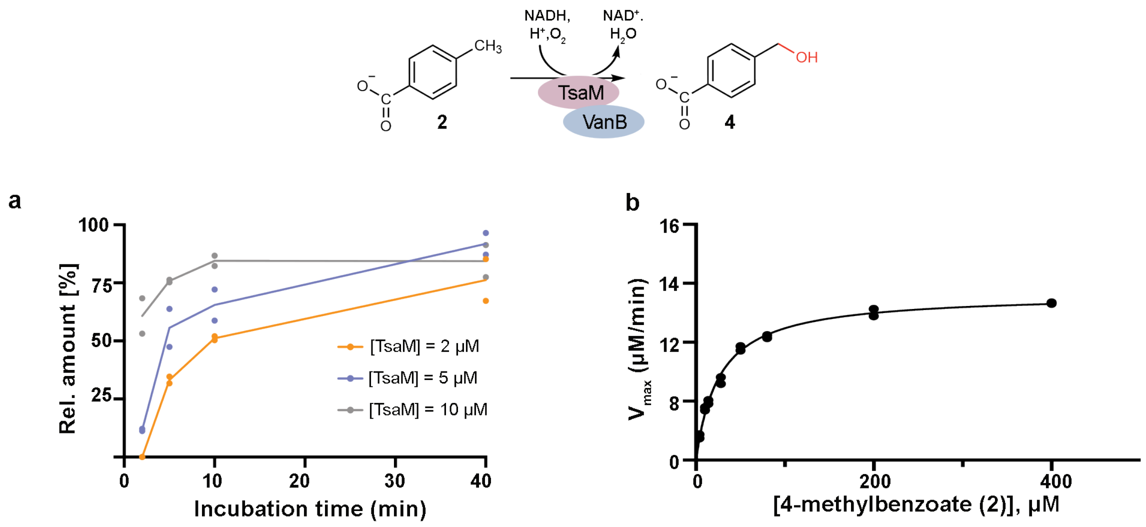

Supplementary Figure 8. The apparent kinetic parameters for the TsaM-VanB system were measured using the second reported native substrate, 4-methylbenzoate (**2**). (a) The linear range for production of 4-(hydroxymethyl)benzoate (**4**) was investigated. This measurement suggested a measurement time of 5 min with 2 μM of TsaM is appropriate for the kinetic measurements. (b) Fitting of the data to the Michaelis-Menten equation revealed the kinetic parameters for the reaction (see Table 1). In this figure, the data were measured using n = 2 independent experiments**.** Source data are provided as a Source Data file.

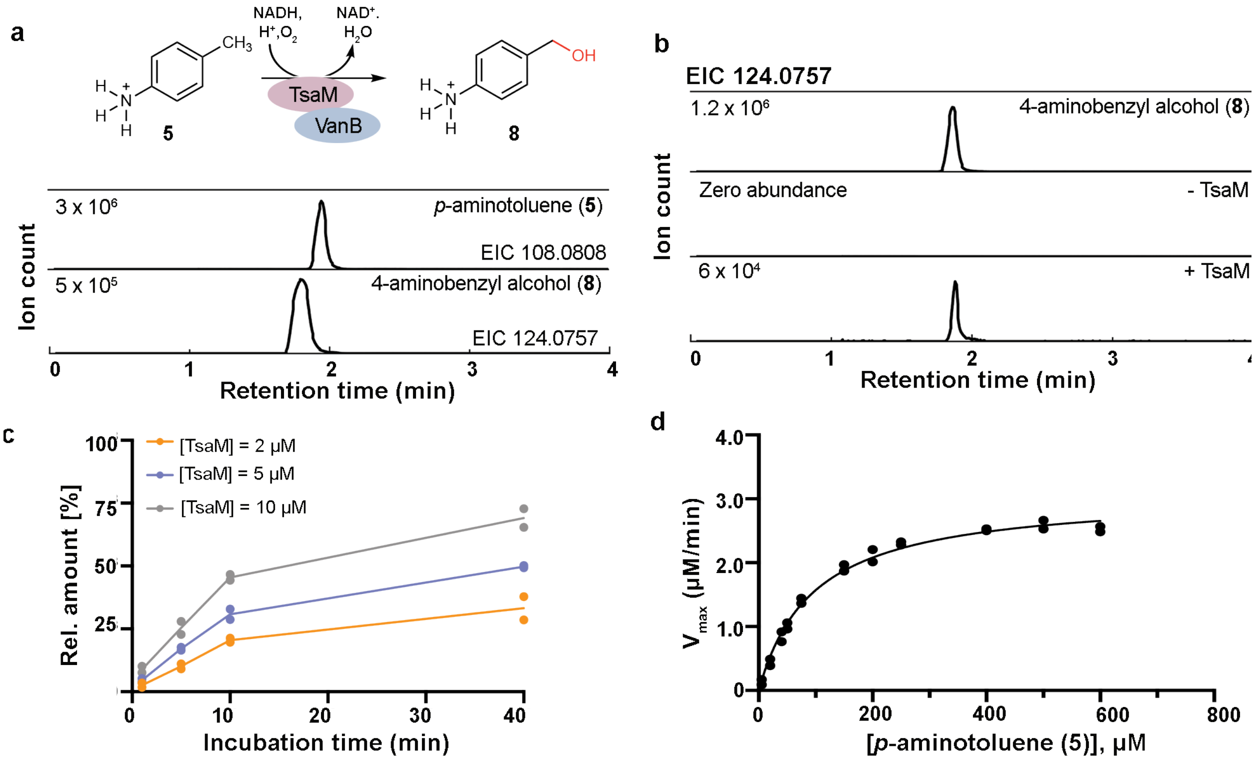

Supplementary Figure 9. The apparent kinetic parameters for the TsaM-VanB system were measured using *p*-aminotoluene (**5**) as a substrate. (a) An LC-MS method was created that permits separation of **5** from 4-aminobenzyl alcohol (**8**). The extracted ion chromatograms for standards of these molecules are shown in this panel. (b) The TsaM-VanB system converts **5** into the expected product, **8** (m/z=124.0757). (c) The linear range of product formation was investigated, and it was determined that 5 min with 5 μM of TsaM is an optimal time for running the assays. (d) Fitting of the data to the Michaelis-Menten equation revealed the kinetic parameters for the reaction (see Table 1). In this figure, the data in panels c and d were measured using n = 2 independent experiments. Source data are provided as a Source Data file.

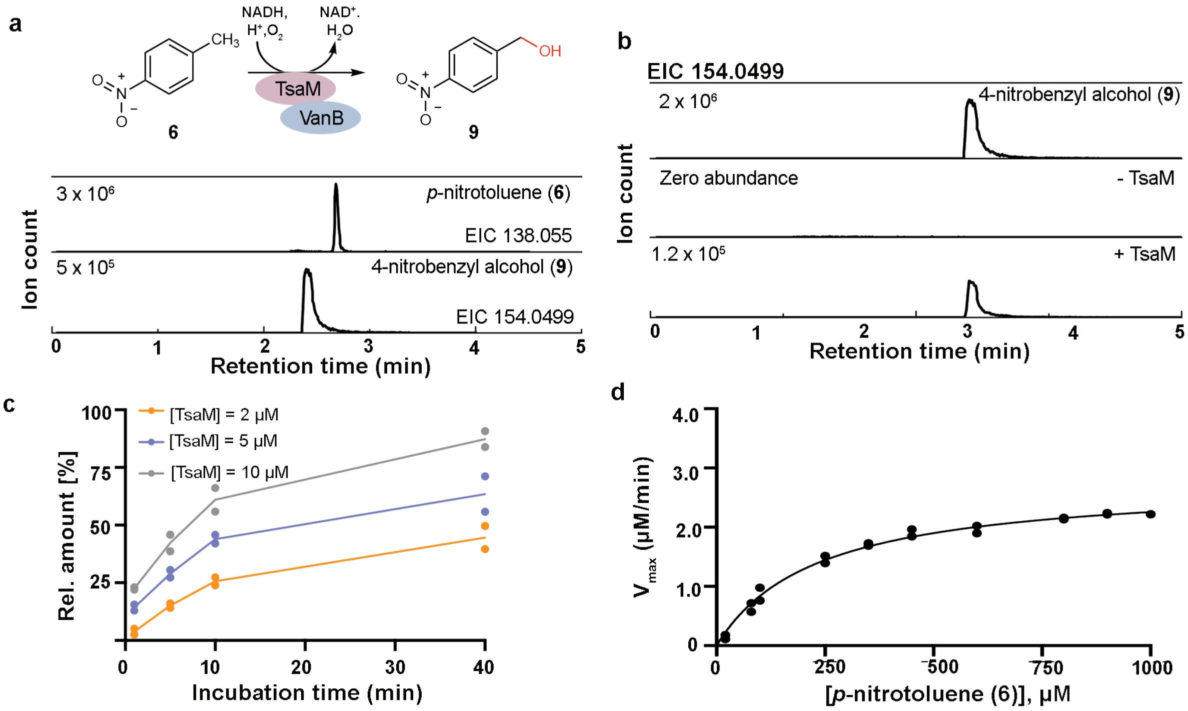

Supplementary Figure 10. The apparent kinetic parameters for the TsaM-VanB system were measured using *p*-nitrotoluene (**6**) as a substrate. (a) An LC-MS method was created to separate a standard of **6** from a standard of 4-nitrobenzyl alcohol (**9**). (b) LC-MS shows that combination of the TsaM-VanB system with **6** results in formation of **9** (m/z=154.0499). (c) The linear range of product formation was investigated, and it was determined that 5 min with 5 μM enzyme is appropriate for running the assays. (d) Fitting of the data to the Michaelis-Menten equation revealed the kinetic parameters for the reaction (see Table 1). In this figure, the data in panels c and d were measured using n = 2 independent experiments. Source data are provided as a Source Data file.

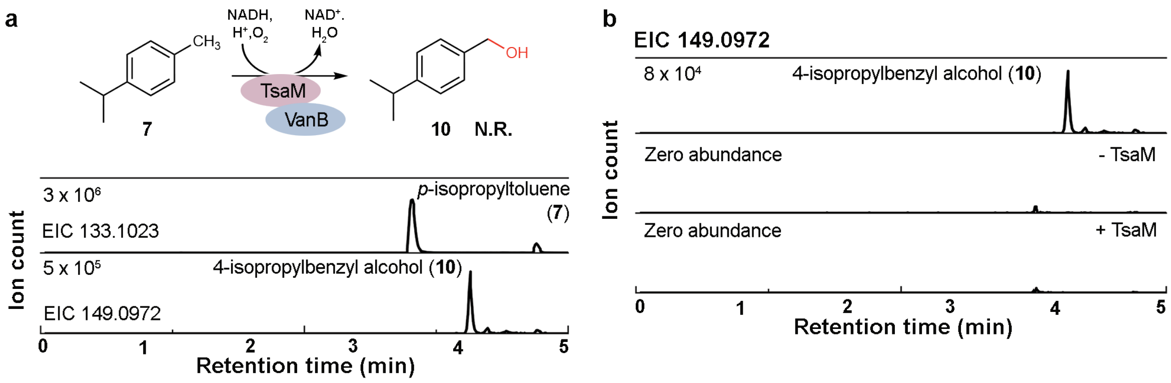

Supplementary Figure 11. The TsaM-VanB system does not show activity on *p*-isopropyltoluene (**7**). (a) To evaluate the ability of the TsaM-VanB system to accept **7** as a substrate, an LC-MS method was created to separate **7** from 4-isopropylbenzyl alcohol (**10**). N.R. indicates no reaction. (b) Using the determined LC-MS method from panel a, it was shown that production of **10** (m/z=149.0972) is not observed when *p*-isopropyltoluene is provided as a substrate.

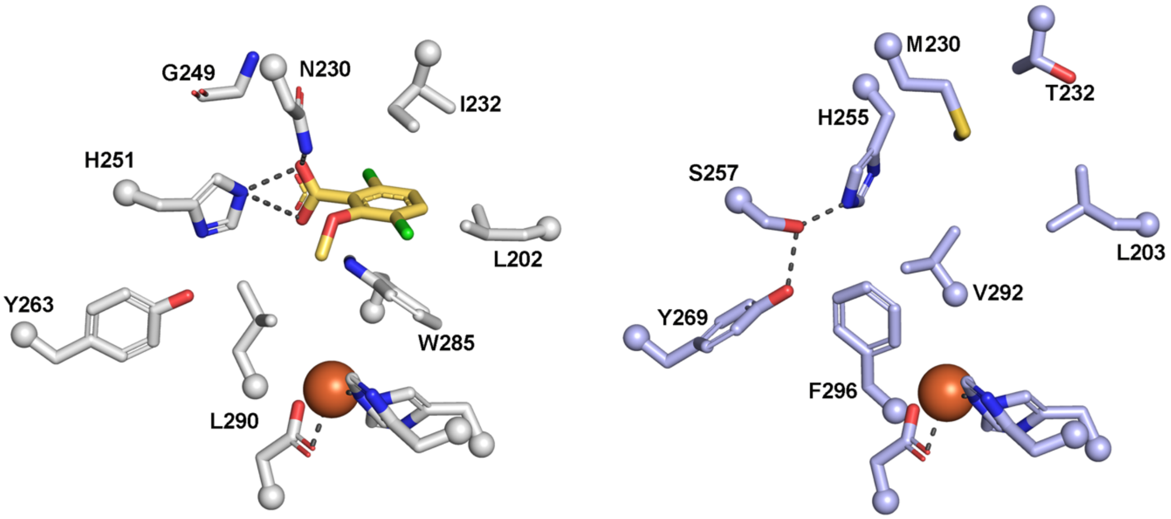

Supplementary Figure 12. Comparison of the substrate (dicamba) bound structure of DdmC (PDB: 3GL2[5]) with an AlphaFold[6,7] model of TsaM reveals a potential triad of polar residues (Ser257, His255, and Tyr269) for interacting with the carboxylate or sulfonate functional groups of the *p*-toluenesulfonate or 4-methylbenzoate substrates, respectively. The AlphaFold[6,7] model of TsaM was generated using previously described methods[4]. Both structures were visualized and analyzed using Pymol 2.5.2_93 software.

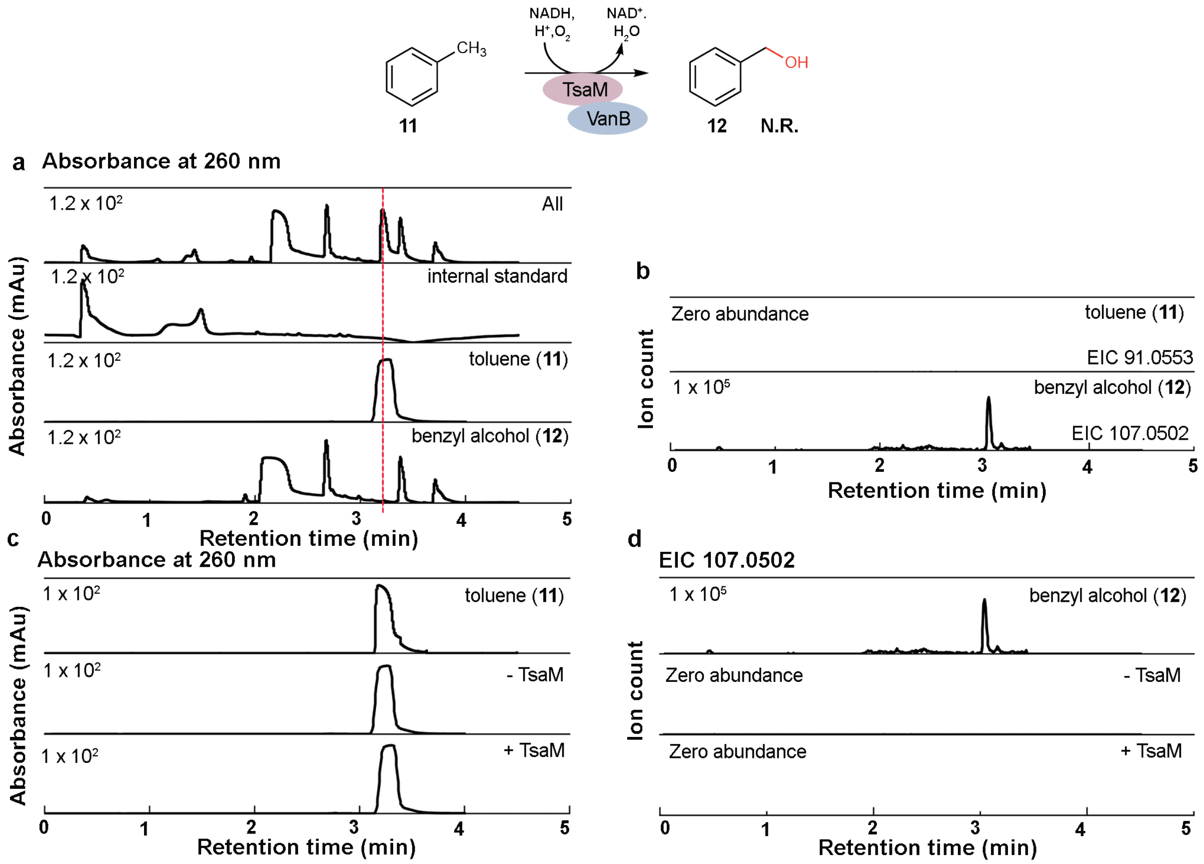

Supplementary Figure 13. Activity assays on the TsaM-VanB system reveal that toluene (**11**) is not a substrate of TsaM. (a) Due to the low ionizability of **11**, its presence was monitored using a diode array detector to identify peaks at 260 nm that had a retention time of 3.2 mins. The red dashed line is showing the alignment of the substrate standard with its presence in the reaction mixture. The top trace of this panel shows the combination of all three standards (substate, product, and internal). (b) An LC-MS method was created to monitor formation of the benzyl alcohol (**12**) product of the reaction (m/z=107.0502). As described in panel a, no peak was observed for toluene (**11**) due to its low ionizability. (c) Using the UV-Vis absorbance peak at 260 nm to detect **11** reveals that no substrate is consumed throughout the duration of the experiment. (d) The extracted ion chromatograms for the TsaM-VanB reaction products using the established LC-MS methods for measuring product formation reveal that the TsaM-VanB system does not produce an oxygenated product when combined with **11**. N.R. indicates no reaction.

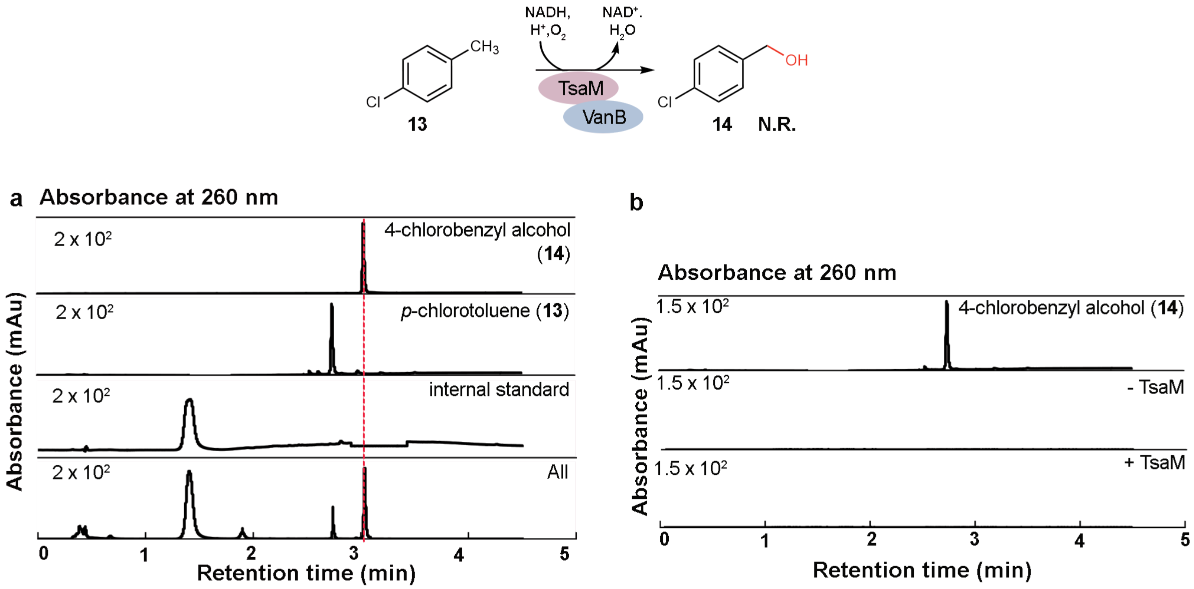

Supplementary Figure 14. Activity assays on the TsaM-VanB system reveal that *p*-chlorotoluene (**13**) is not a substrate of TsaM. (a) Due to the low ionizability of both the substrate **13** and expected 4-chlorobenzyl alcohol (**14**) product, the presence of **13** and **14** was monitored using a diode array detector to find peaks at 260 nm that had retention times near 3 mins. The red dashed line shows the alignment of the product standard (**14**) in each of the different traces. The bottom trace contains all of the standards. (b) The UV-Vis absorbance peak at 260 nm was used to detect the production of **14** in the enzymatic reaction. This detection method revealed that no oxygenated product is formed. N.R. indicates no reaction.

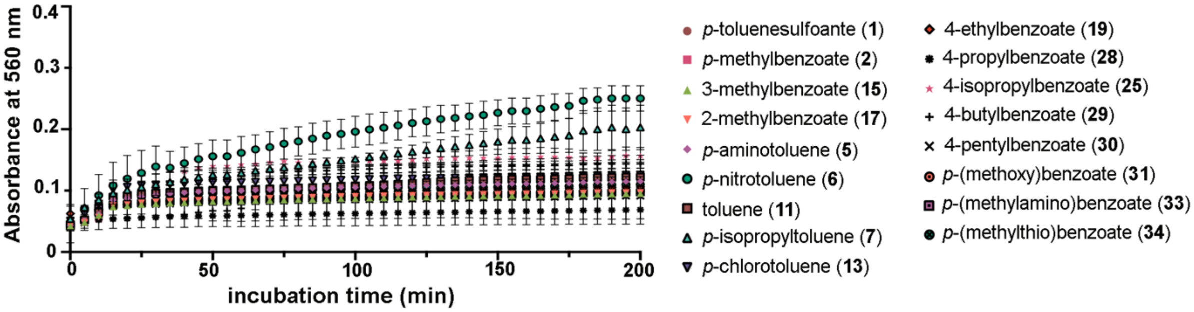

Supplementary Figure 15. An uncoupling assay reveals that supplying *p*-nitrotoluene or *p*-isopropyltoluene as a substrate to the TsaM-VanB system leads to uncoupling of O_2 activation from substate functionalization. Using previously described methods and a standard curve[4], uncoupling was investigated for TsaM with different substrates. Specifically, it was determined that he absorbance at 560 nm does not change when the TsaM-VanB system is combined with *p*-toluenesulfonate, *p*-methylbenzoate, and many of the other tested substrates over a 3 h incubation. However, when TsaM-VanB is incubated with *p*-nitrotoluene or *p*-isopropyltoluene, an increased amount of H_2O_2 formation is detected. In this figure, all the data were measured in n = 3 independent experiments and are presented as the mean value ± SD of these measurements. Source data are provided as a Source Data file.

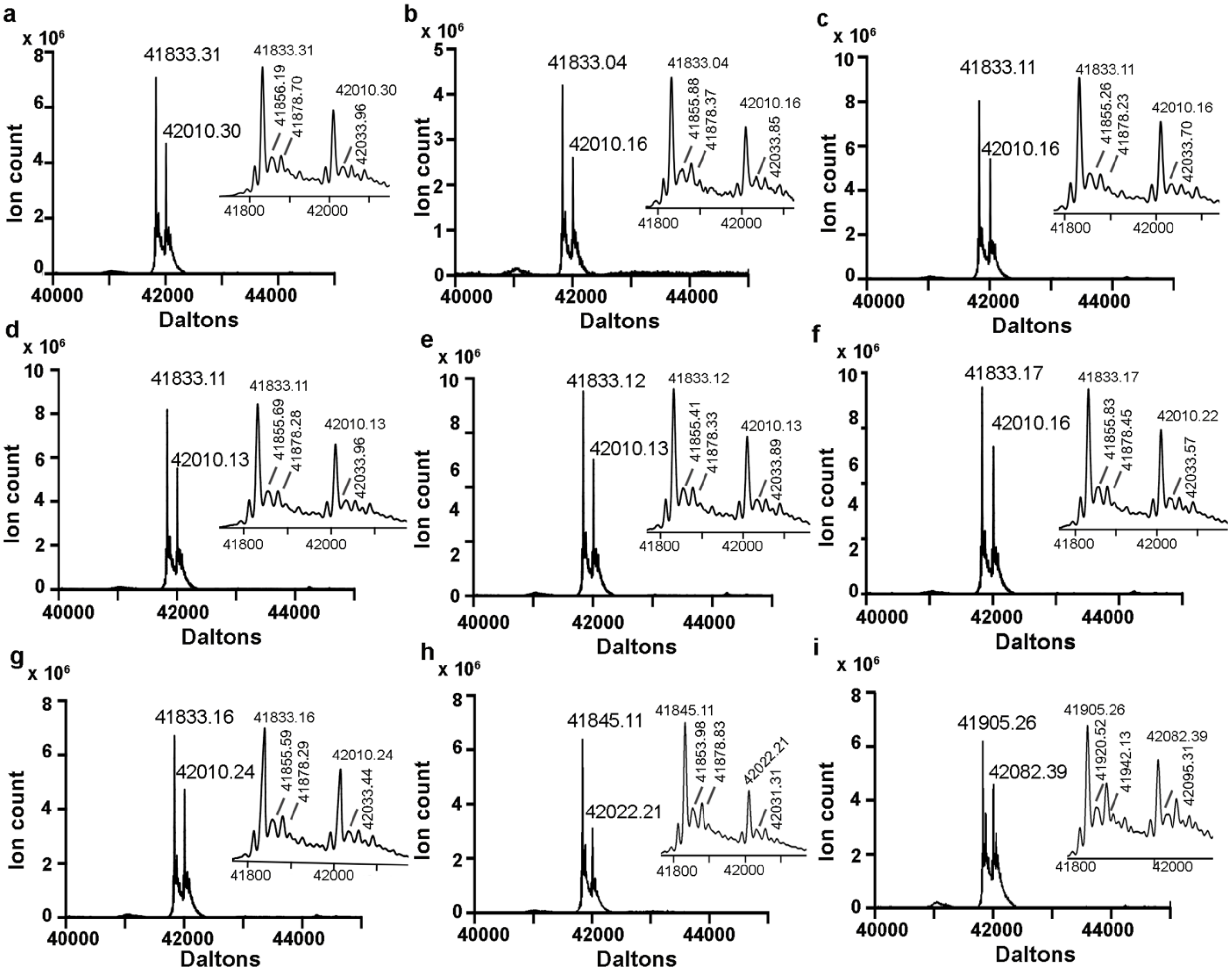

Supplementary Figure 16. Intact protein mass spectrometry (MS) experiments reveal that TsaM is not oxidatively modified in reactions that show increased formation of H_2O_2 relative to TsaM with *p*-toluenesulfonate and 4-methylbenzoate substrates. Intact mass spectrometry experiments are shown for reactions that contained (a) TsaM and VanB, (b) TsaM-VanB and *p*-toluenesulfonate, (c) TsaM-VanB and 4-methylbenzoate, (d) TsaM-VanB and *p*-nitrotoluene, (e) TsaM-VanB and *p*-isopropyltoluene, (f) TsaM-VanB and phenylacetate, (g) TsaM-VanB and 4-methylphenylacetate, (h) TsaM T232I-VanB and benzoate, and (i) TsaM M230W/T232I/S257R/Y269V-VanB and benzoate. In panels a-g, 42010 Da corresponds to the actual mass of TsaM and 41833 Da is the mass of TsaM after losing the N-terminal methionine residue (-131 Da) and the C-terminal carboxylate moiety (-46 Da). In panels h and i, the actual mass of TsaM is 42022 Da and 42082 Da, respectively.

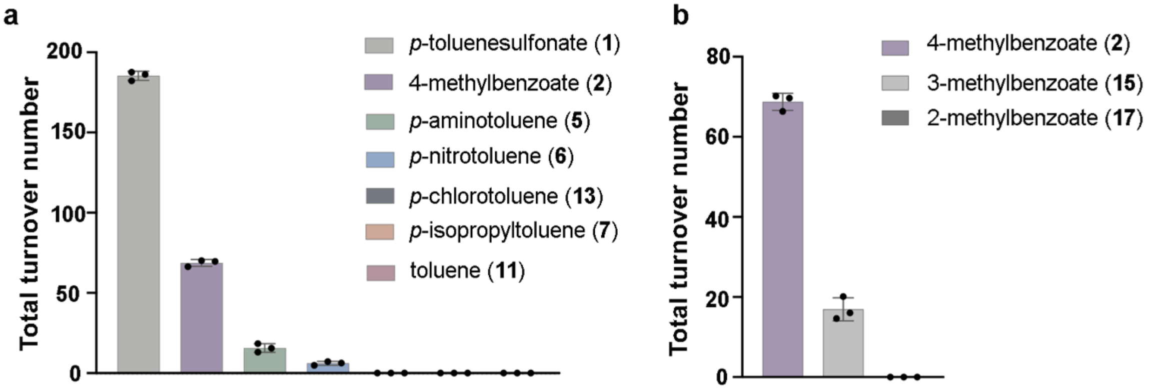

Supplementary Figure 17. TsaM demonstrates a preference for oxygenating the methyl group at the *p*-position of substrates that contain a polar functional group. (a) TsaM accepts and oxygenates a range of substrates that are described in the text and contain a polar functional group (sulfonate, carboxylate, amino, or nitro) at the *p*-position. This data complements the heat map data shown in Figure 3a. (b) TsaM accepts and oxygenates compounds that contain the methyl group at the *p*- or *m*- position. This data reflects previously measured and described trends for TsaM with these substrates[4] and is included to complement the heat map data shown in Figure 3b. In both panels, data were measured using n = 3 independent experiments and is are presented as mean values ± SD. Source data are provided as a Source Data file.

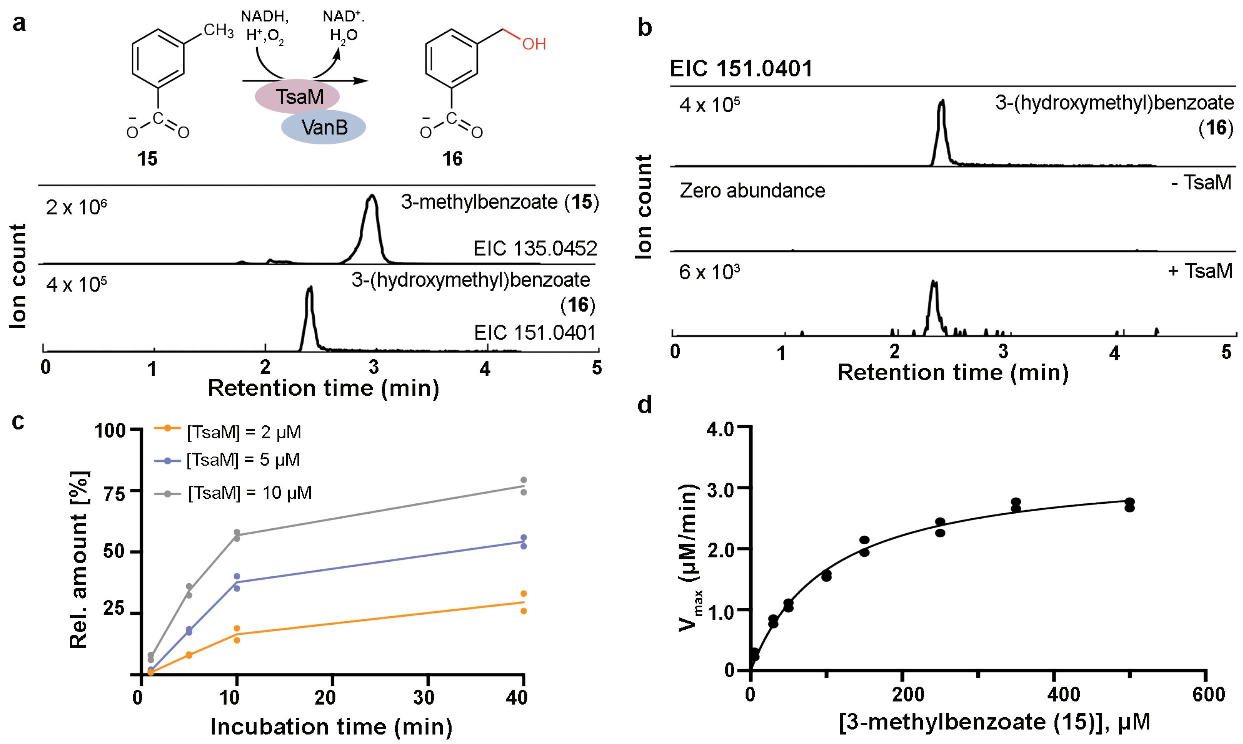

Supplementary Figure 18. The TsaM-VanB system accepts and oxygenates a 3-methylbenzoate (**15**) substrate. (a-b) To verify the previously determined[4] ability of the TsaM-VanB system to accept and oxygenate **15**, an LC-MS method was used to separate **15** from the expected 3-(hydroxymethyl)benzoate (**16**) product. (c) To add to previous knowledge, the linear range of product formation was investigated, and it was determined that 5 min with 5 μM of TsaM is an optimal time for running the assays. (d) Fitting of the data to the Michaelis-Menten equation revealed the kinetic parameters for the reaction (see Table 1). In this figure, the data in panels c and d were measured using n = 2 independent experiments. Source data are provided as a Source Data file.

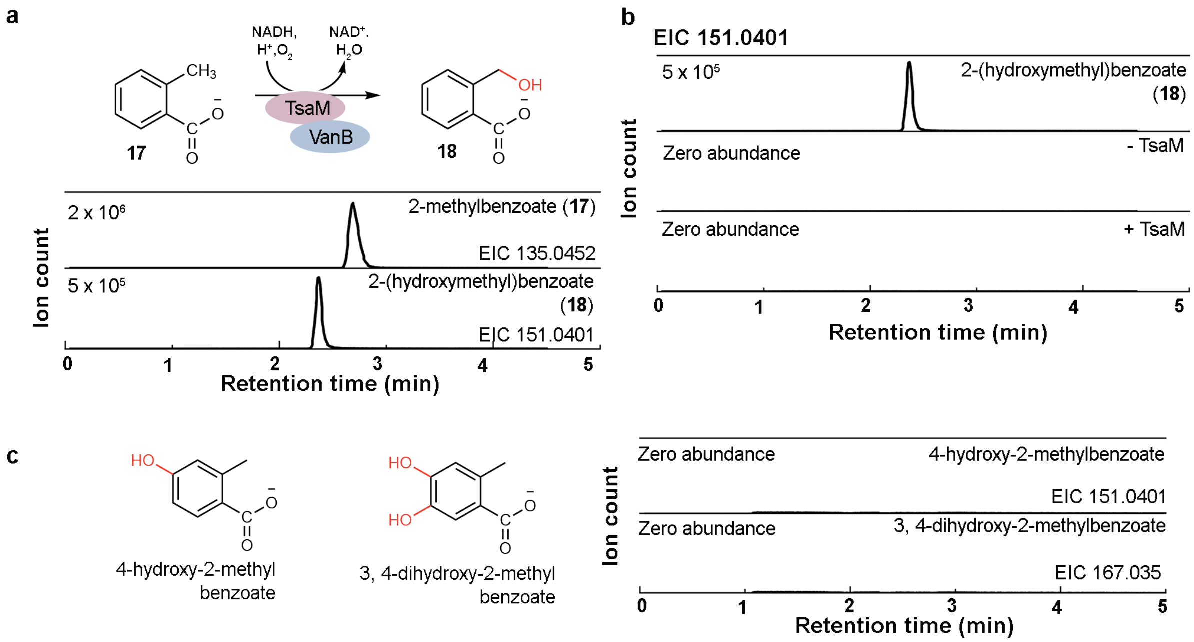

Supplementary Figure 19. Assays on the TsaM-VanB system with a 2-methylbenzoate (**17**) substrate reveal that movement of the methyl group from the *para*- to the *ortho*-position is detrimental to the activity of TsaM. (a-b) To verify the previously determined[4] inability of TsaM to oxygenate **17**, an LC-MS method was customized for separating **17** and the expected 2-(hydroxymethyl)benzoate (**18**) product (m/z=151.0401). (c) The LC-MS trace also indicates that there is no formation of 4-hydroxy-2-methylbenzoate (a single hydroxylated product at the *para*-position with m/z = 151.0401), nor is there formation of a 3,4-dihydroxy-2-methylbenzoate product (a dihydroxylated product at both the *para*- and *meta*-positions with m/z = 167.035) when provided with **17.**

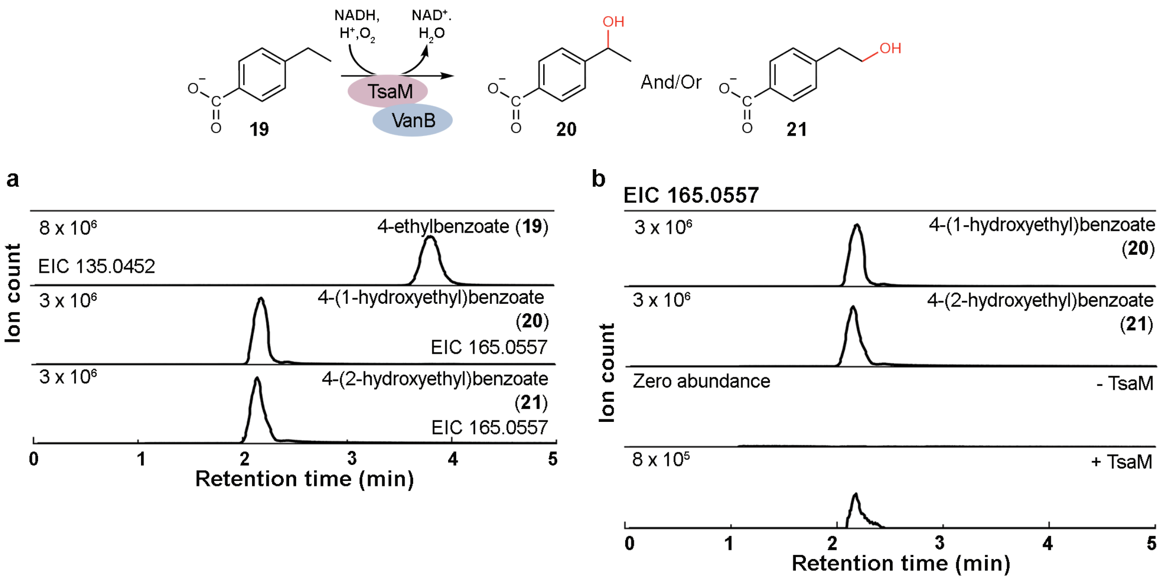

Supplementary Figure 20. Combination of the TsaM-VanB system with a 4-ethylbenzoate (**19**) substrate results in the formation of a monooxygenated product. (a) Separation of 4- ethylbenzoate and two of its potential monooxygenated products 4-(1-hydroxyethyl)benzoate (**20**) and 4-(2-hydroxyethyl)benzoate (**21**). (b) The extracted ion chromatograms of the TsaM-VanB reaction when provided with **19** reveals formation of a monooxygenated product (m/z=165.0557). The top two traces correspond to the two possible products (**20** and **21**).

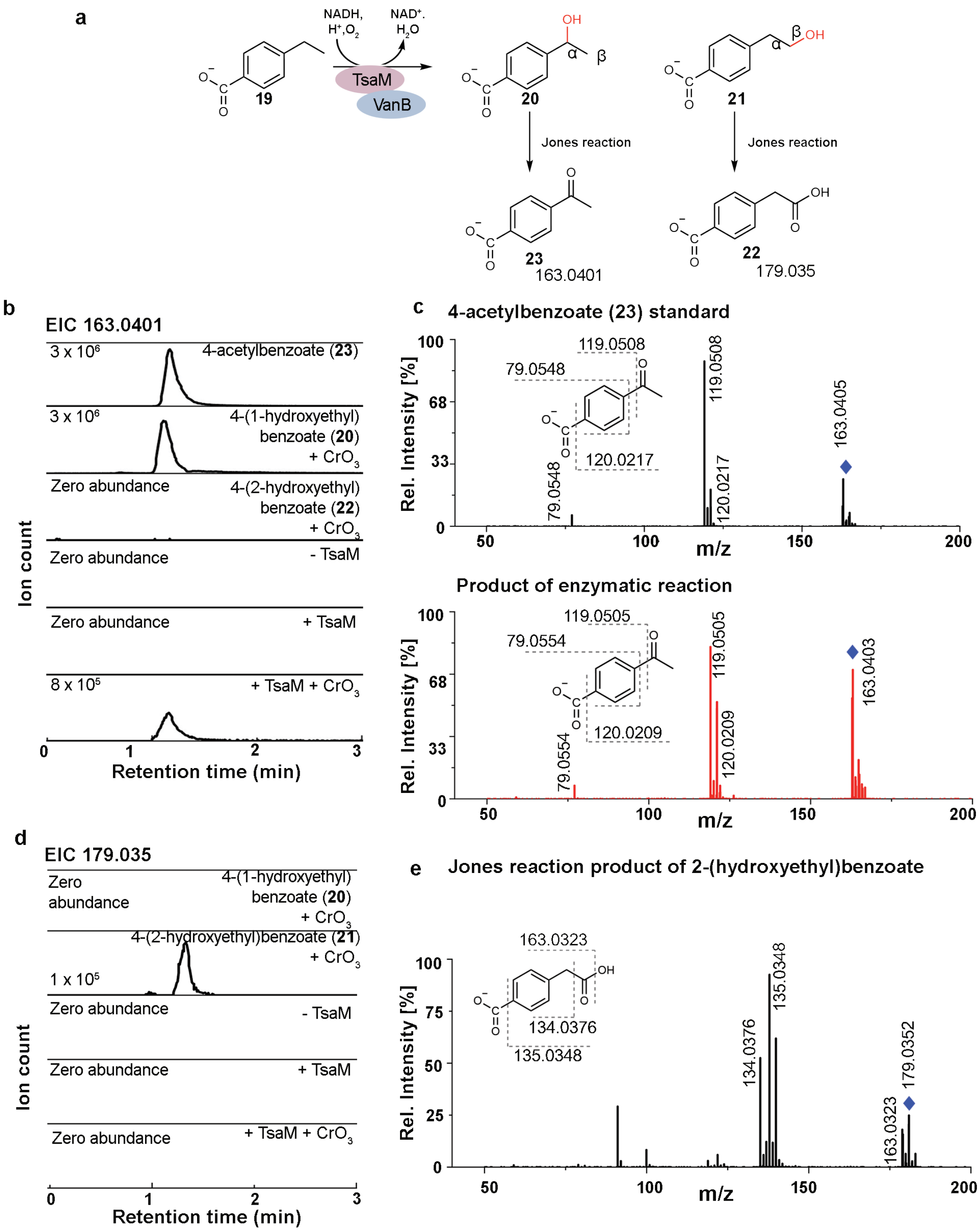

Supplementary Figure 21. The Jones reaction indicates that TsaM selectively hydroxylates the Cα position of **19** to generate 4-(1-hydroxyethyl)benzoate (**20**). (a) The reaction scheme for coupling the Jones reagent with the potential enzymatic products. (b) The extracted ion chromatograms reveal formation of the ketone product, 4-acetylbenzoate (**23,** m/z=163.0401), indicating the identity of the reaction product is **20**. (c) The product identity was confirmed by comparison of the MS/MS fragmentation patterns of the ketone product standard (**23**) and the Jones oxidation product. (d) Consistent with **20** being the product of the TsaM-VanB reaction, Jones reagent does not form a carboxylic acid product, 4-(carboxymethyl)benzoate (**22**). (e) The MS/MS fragmentation of **22** (**21** treated with Jones reagent) also supports the identification of the product of this reaction being **20.**

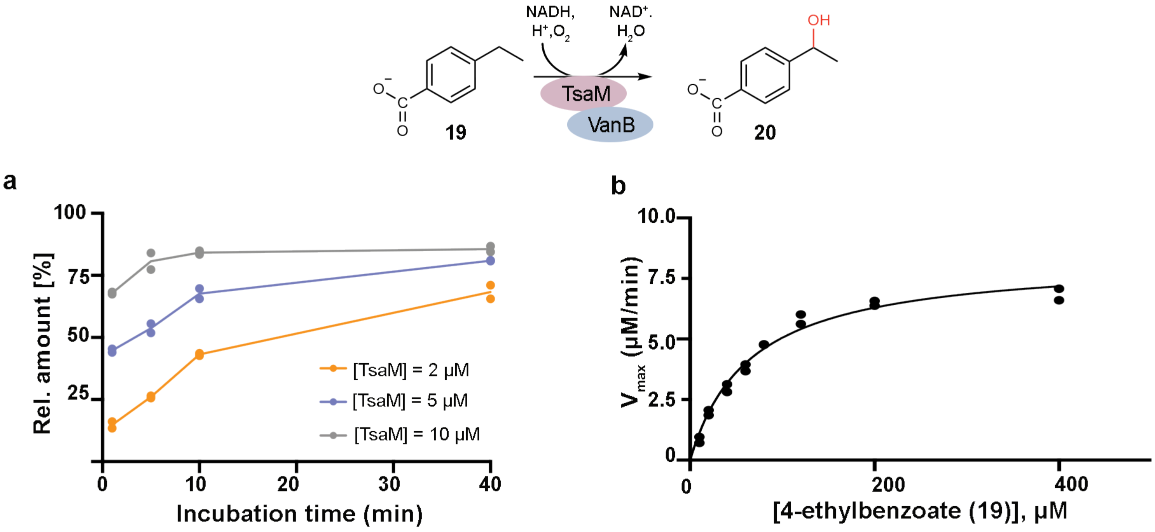

Supplementary Figure 22. As the TsaM-VanB system shows the ability to catalyze a monooxygenation reaction on a 4-ethylbenzoate (**19**) substrate, the apparent kinetic parameters were investigated. (a) The linear range of product formation was investigated, and it was determined that 5 min with 5 μM enzyme is an optimal time for running the assays. (b) Fitting of the data to the Michaelis-Menten equation revealed the apparent kinetic parameters for the reaction (see Table 1). In this figure, the data were measured using n =2 independent experiments**.** Source data are provided as a Source Data file.

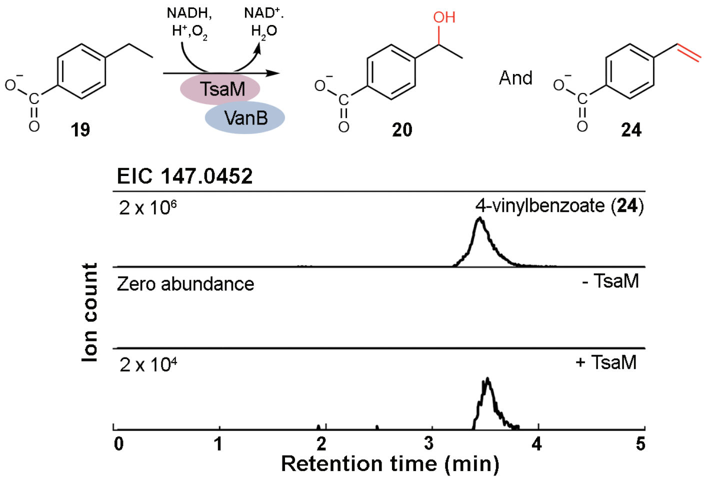

Supplementary Figure 23. In addition to forming a major 4-(1-hydroxyethyl)benzoate (**20**) product, the reaction of TsaM-VanB with **19** results in formation of a minor product, 4-vinylbenzoate (**24**). These products are formed in an 11:1 ratio of 4-(1-hydroxyethyl)benzoate (**20**): 4-vinylbenzoate (**24**). The amount of **20** and **24** generated was calculated by using a constructed standard curve of **20** and **24** (see Supplementary Figure 73h). Here, the top trace corresponds to a product standard of **24** and the bottom trace shows production of **24** in the presence of TsaM-VanB.

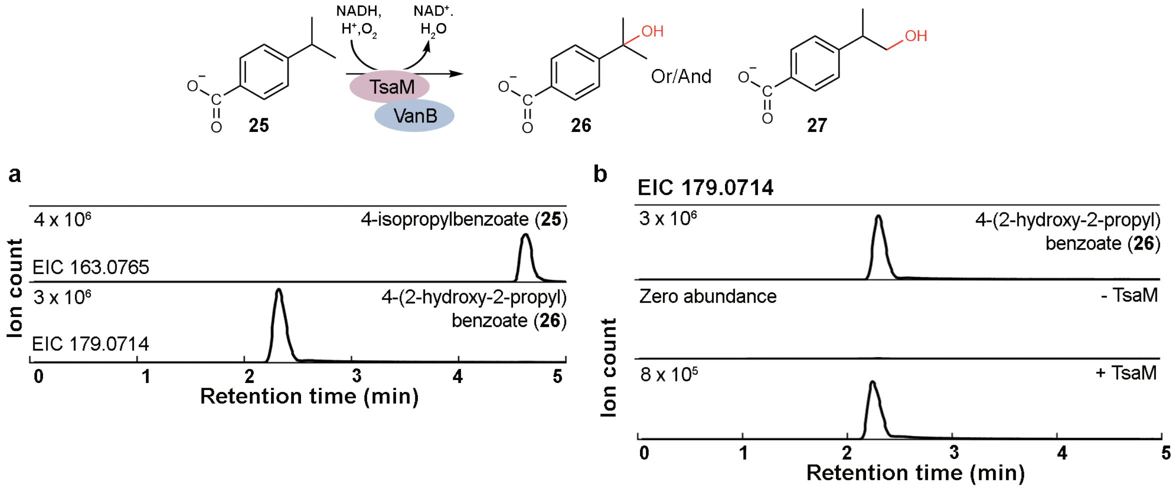

Supplementary Figure 24. The TsaM-VanB system accepts a 4-isopropylbenzoate substrate (**25**). (a) To probe the competence of 4-isopropylbenzoate as a substrate, LC-MS methods were developed to separate **25** from the proposed product 4-(2-hydroxy-2-propyl)benzoate (**26**). The product standard for 4-(1-hydroxy-2-propyl)benzoate (**27**) is not available and was not included in this analysis. (b) The extracted ion chromatogram of the reaction between the TsaM-VanB system and the 4-isopropylbenzoate substrate shows formation of a monooxygenated product.

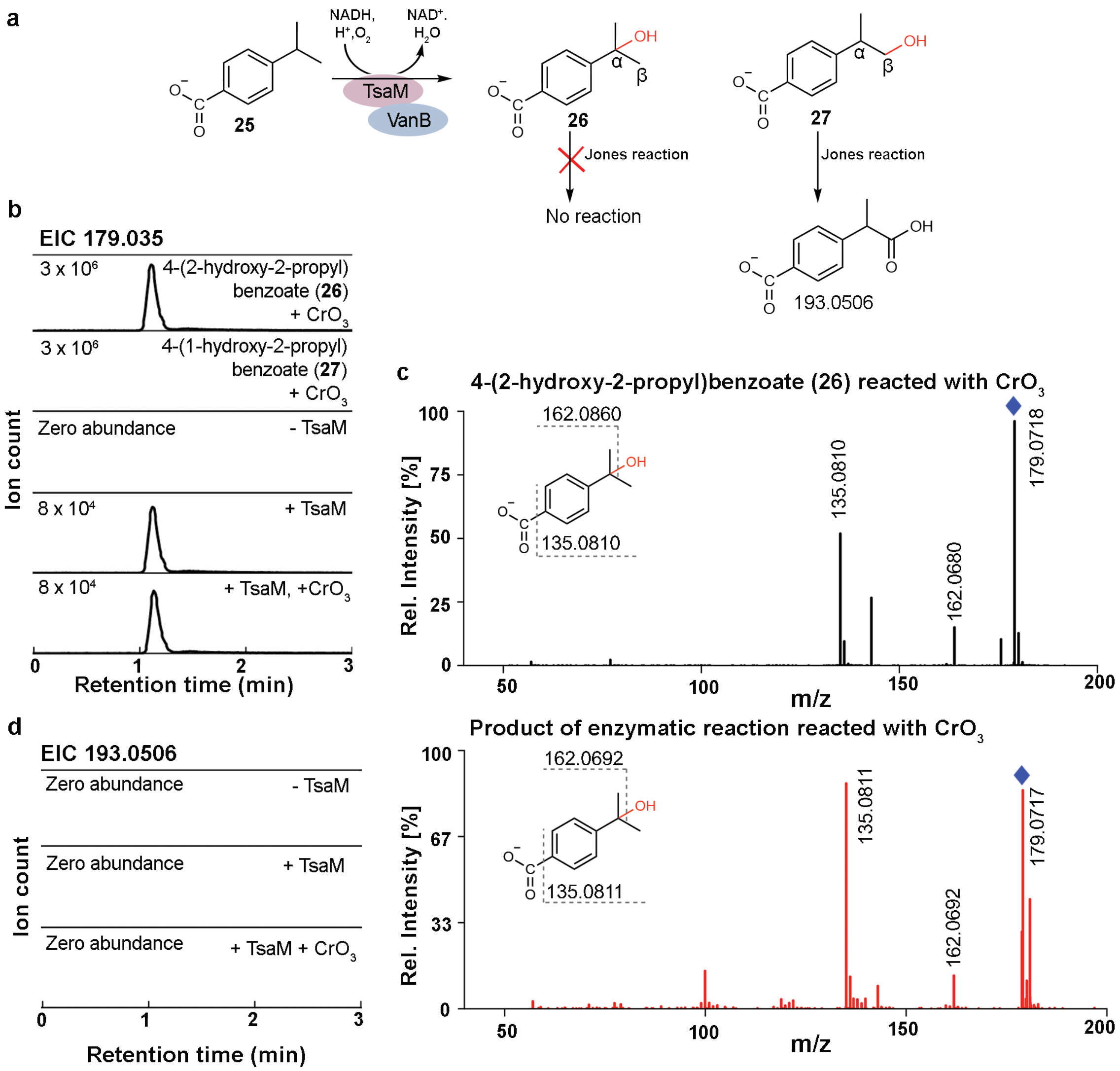

Supplementary Figure 25. The Jones reaction indicates that TsaM selectively hydroxylates the Cα position of 4-isopropylbenzoate (**25**) to generate 4-(2-hydroxy-2-propyl)benzoate (**26**). (a) The reaction scheme for coupling Jones reagent with the potential enzymatic products. (b) Based on the lack of formation of a Jones reaction product, it was presumed that the product of the reaction is **26**. (c) The product identity was confirmed by comparison of the MS/MS fragmentation patterns of a standard of **26** and the reaction product treated with Jones reagent. (d) As revealed in LC-MS, consistent with **26** being the product of the TsaM-VanB reaction, Jones reagent does not form a carboxylic acid (m/z=193.0506).

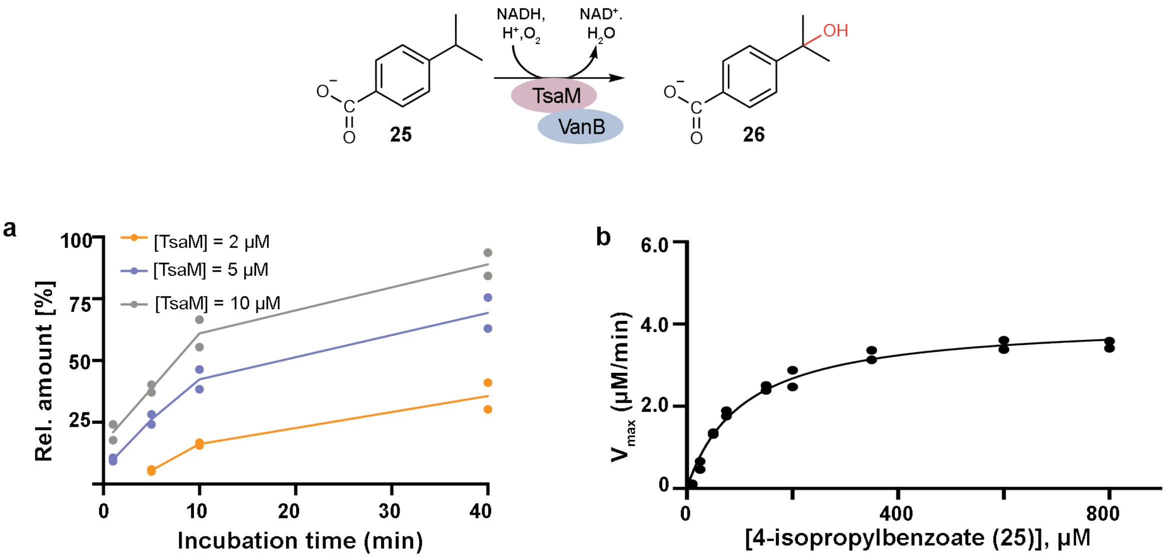

Supplementary Figure 26. As the TsaM-VanB system demonstrates the ability to catalyze a monooxygenation reaction on a 4-isopropylbenzoate substrate (**25**), the apparent kinetic parameters were investigated. (a) First, the linear range of product formation was investigated, and it was determined that 5 min with 5 μM enzyme is an optimal time for running the assays. (b) Using LC-MS to measure product formation over a range of concentrations of **25**, the Michaelis-Menten kinetic parameters for the reaction were determined (see Table 1). In this figure, the data were measured using n = 2 independent experiments. Source data are provided as a Source Data file.

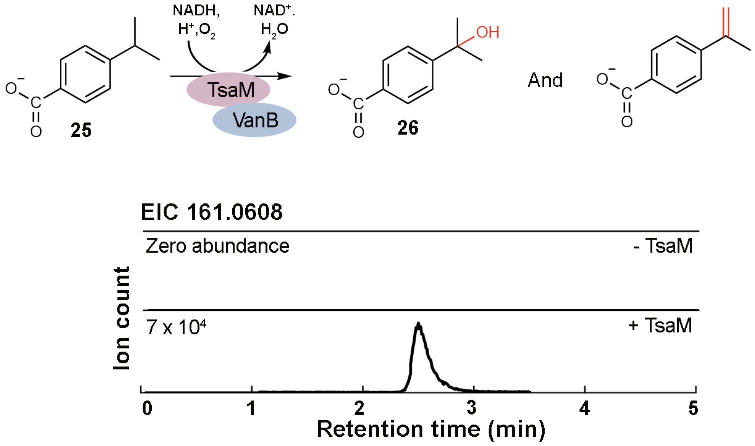

Supplementary Figure 27. In addition to forming the major product 4-(2-hydroxy-2-propyl)benzoate (**26**), the reaction of TsaM-VanB with **25** results in formation of a minor desaturation product (m/z=161.0608). The ratio of generated **26** to desaturated product is approximately 18:1. The amount of **26** generated was calculated by using a constructed standard curve of **26**. The amount of desaturated product generated was calculated by using the consumed substrate amount (**25**) minus the generated hydroxylated product (**26**) (see Supplementary Figure 74a).

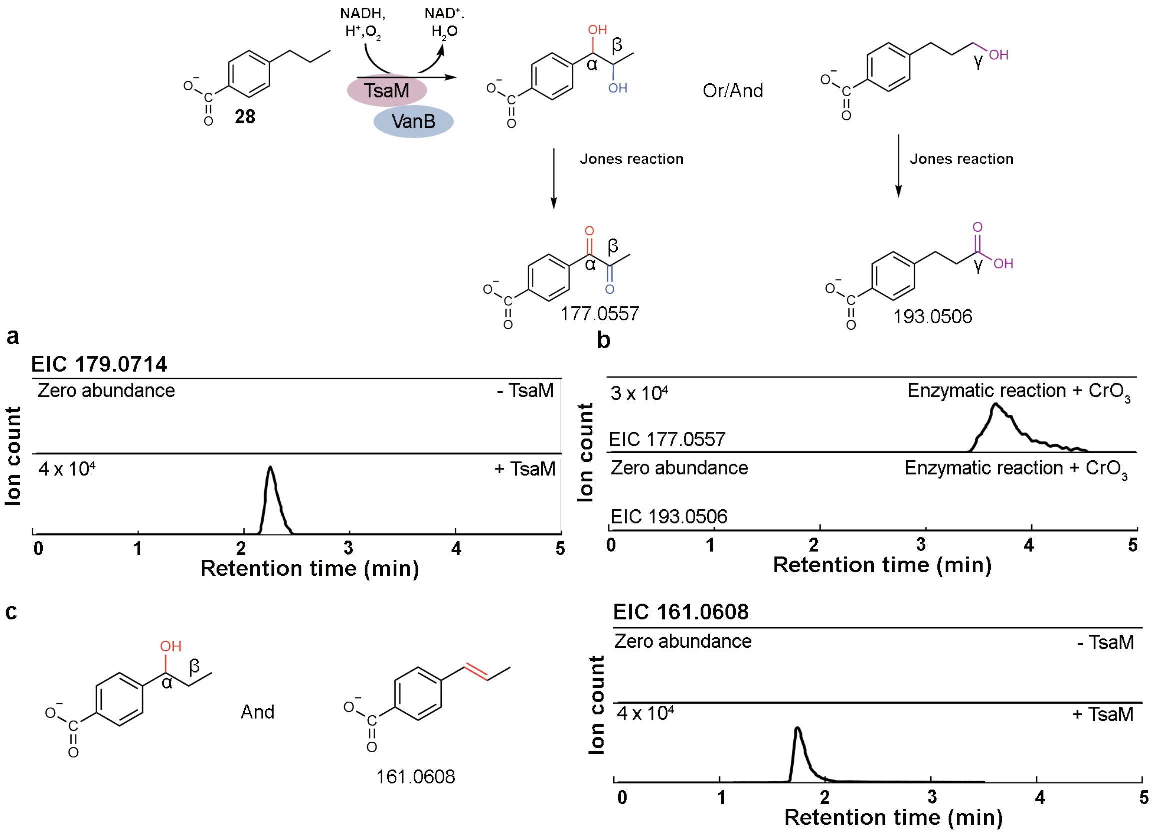

Supplementary Figure 28. To further probe the substrate scope of the TsaM-VanB system, the competence of a 4-propylbenzoate (**28**) substrate was tested. (a) LC-MS revealed that the TsaM-VanB system can perform a monooxygenation reaction on **28**. The product of this reaction has an m/z = 179.0714, which could correspond to the formation of a compound that is oxygenated at the α, β, or γ position. (b) Reaction of the product from the enzymatic assays with Jones reagent results in formation of a ketone product (m/z=177.0557). This result is consistent with the identity of the product in the assay being a secondary, rather than a primary, alcohol (*e.g.* hydroxylation occurred at the α or β position). (c) As observed with the ethyl- and isopropylbenzoate substrates, a desaturation product (m/z=161.0608) is also observed with the 4-propylbenzoate substrate.

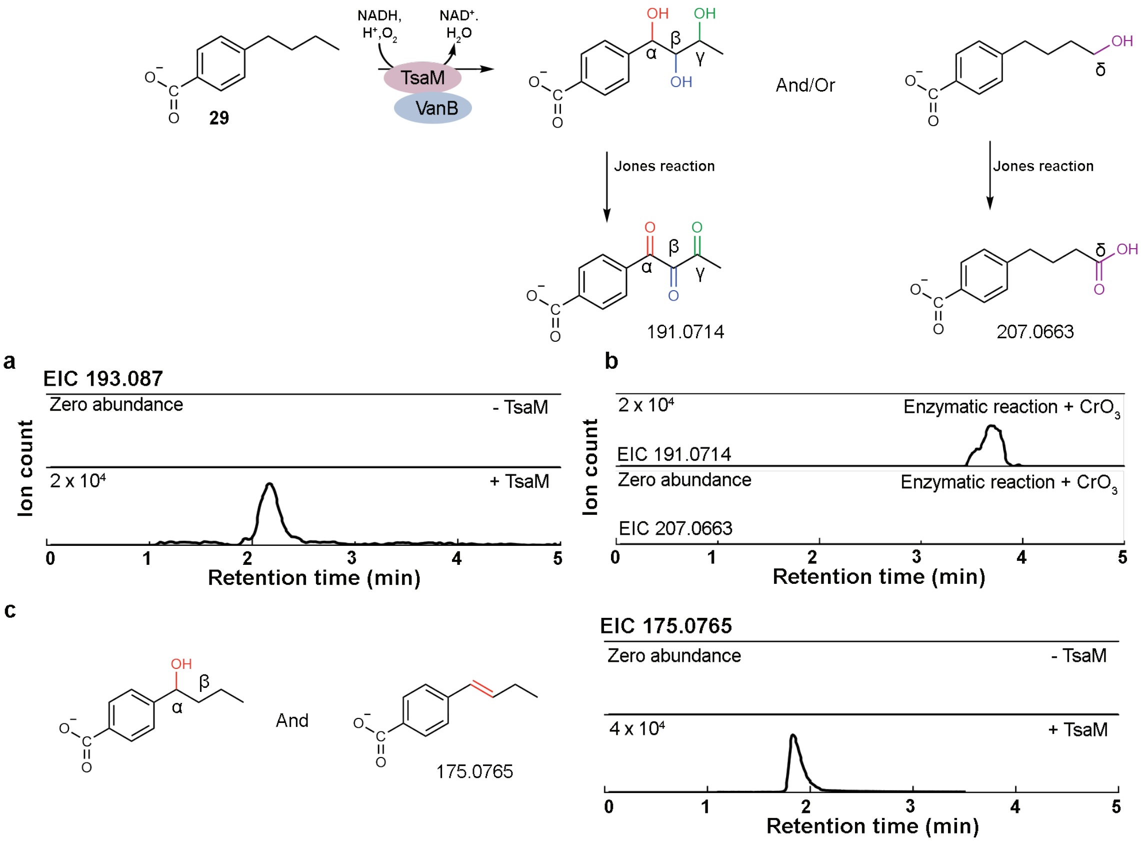

Supplementary Figure 29. Additional studies on the substrate scope of the TsaM-VanB system, included an investigation into the competence of 4-butylbenzoate (**29**) to serve as a substrate. (a) As observed with the ethyl-, isopropyl-, and propylbenzoate substrates, the TsaM-VanB system catalyzes a monooxygenation reaction on **29**. The product formed in this reaction has an m/z = 193.087, which could correspond to production of a compound that is oxygenated at the α, β, γ, or δ position. (b) To identify the product of the TsaM catalyzed reaction, Jones reagent was added to the assay. This second reaction produced a ketone (m/z = 191.0714), which confirms that the product of the enzymatic reaction is a secondary alcohol. (c) In addition to production of a secondary alcohol, the TsaM-VanB system produces a minor desaturation product (m/z = 175.0765).

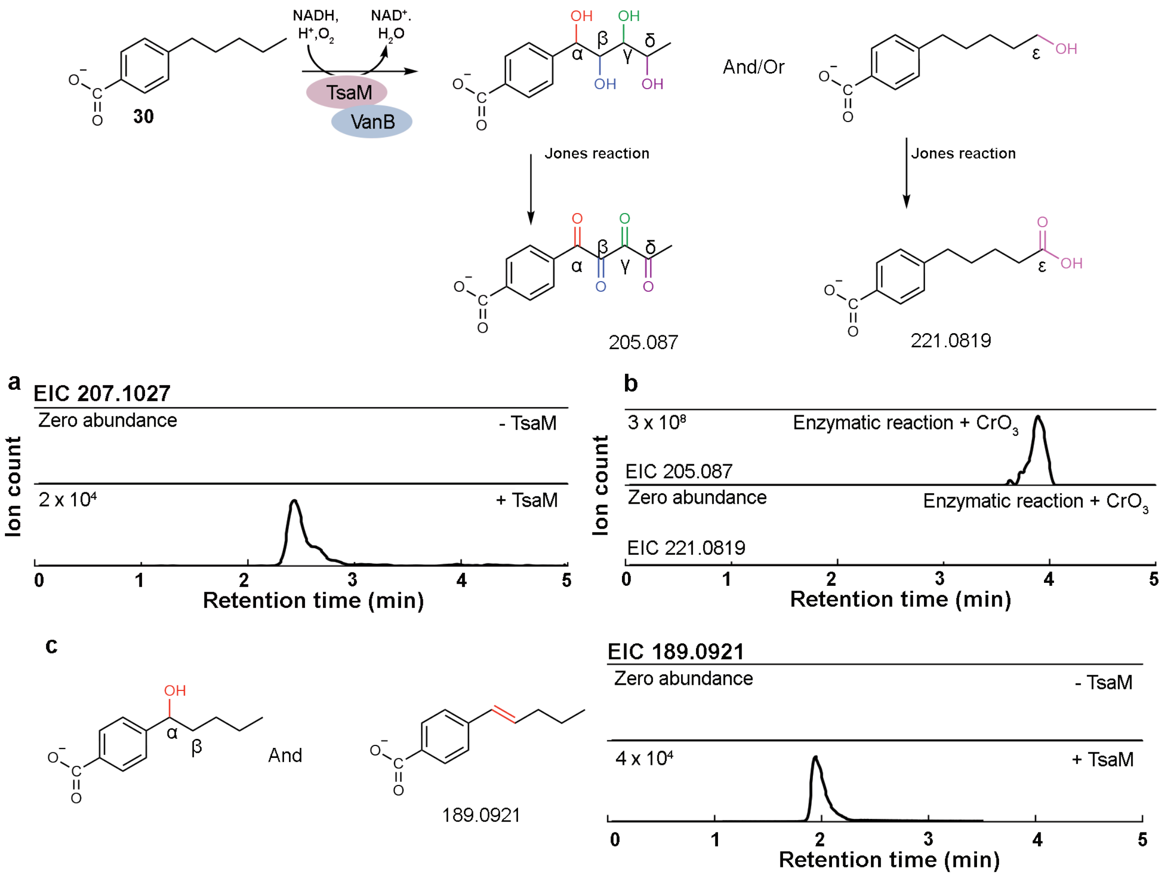

Supplementary Figure 30. The ability of the TsaM-VanB system to accept a 4-pentylbenzoate (**30**) substrate was tested. (a) The TsaM-VanB system catalyzes a monooxygenation reaction on the 4-pentylbenzoate substrate to form a compound that has an m/z = 207.1027. This m/z value could correspond to production of a compound that is oxygenated at the α, β, γ, δ, or ε position. (b) Reaction of the TsaM-VanB product with Jones reagent results in formation of a ketone product (m/z = 205.087) rather than a carboxylic acid (m/z=221.0819) product. This result is consistent with the product of the enzymatic reaction being a secondary alcohol. (c) As described for the other longer chain-length substrates, a desaturated product is also formed in this assay (m/z = 189.0921).

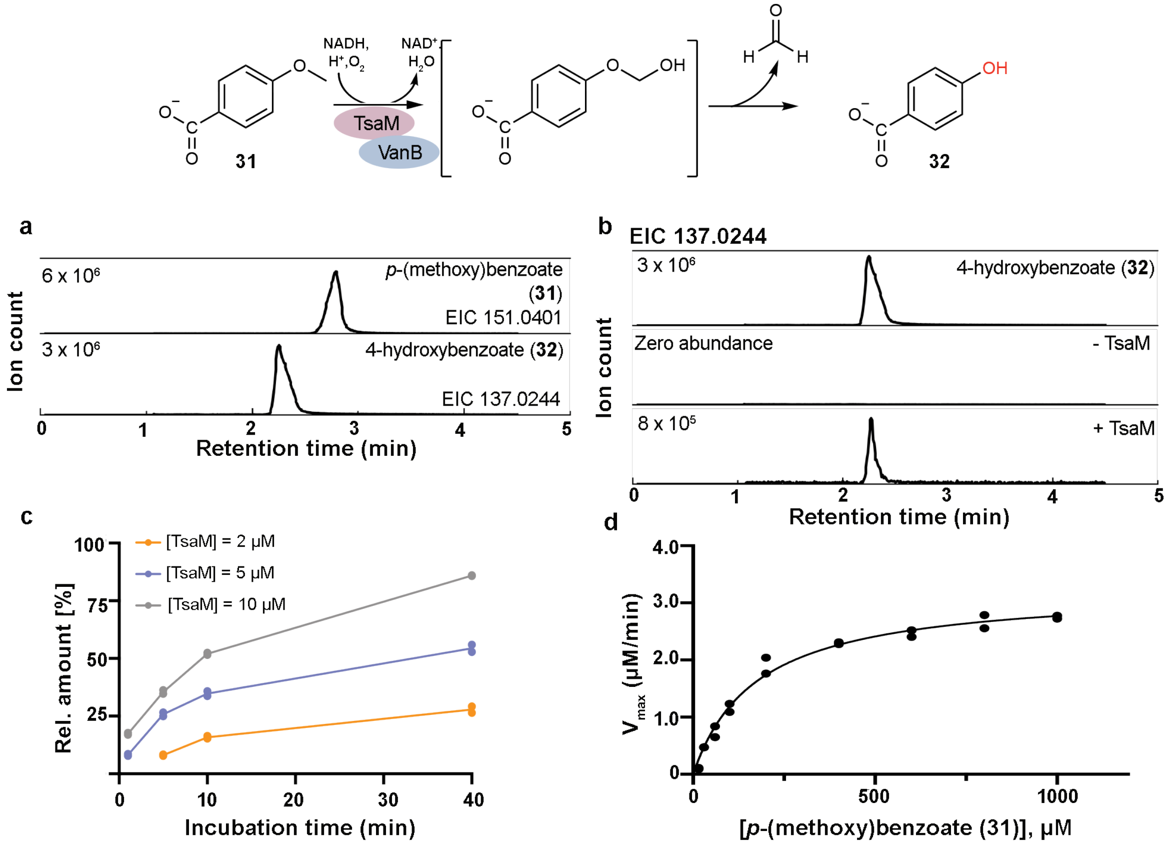

Supplementary Figure 31. The activity of the TsaM-VanB system was also tested with a *p*-(methoxy)benzoate (**31**) substrate. (a) To measure the activity of the TsaM-VanB system on *p*-(methoxy)benzoate, a previously used[4] LC-MS method was implemented to separate standards of *p*-(methoxy)benzoate (m/z = 151.0401) from the predicted 4-hydroxybenzoate (**32**) product (m/z = 137.0244). (b) As expected[4], the TsaM-VanB system accepts **31** as a substrate and produces **32**. (c) The linear range of product formation was investigated, and it was determined that 5 min with 5 μM enzyme is an optimal time for running the assays. (d) Fitting of the collected LC-MS data to the Michaelis-Menten equation over a range of substrate concentrations revealed the apparent kinetic parameters for the reaction (see Table 1). In this figure, the data in panels c and d were measured using n = 2 independent experiments. Source data are provided as a Source Data file.

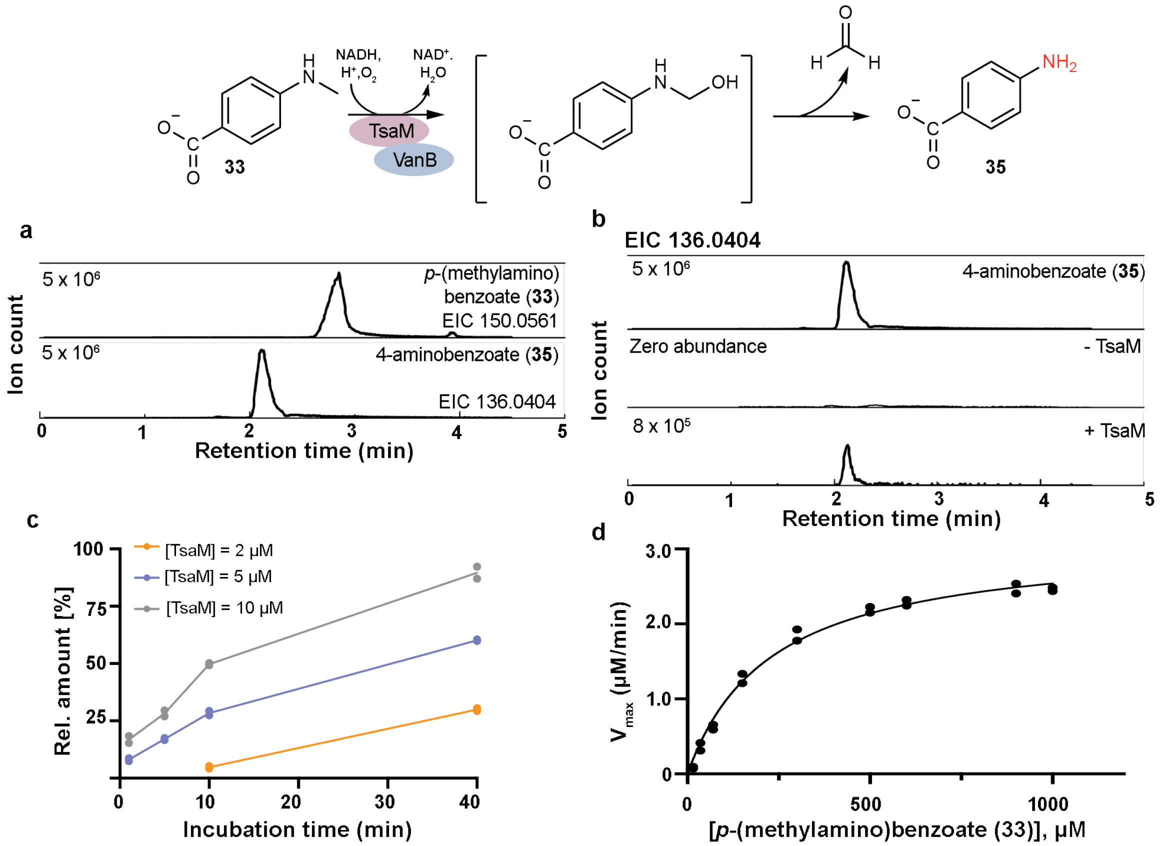

Supplementary Figure 32. The TsaM-VanB system also accepts and oxygenates a *p*-(methylamino)benzoate (**33**) substrate. (a) An LC-MS method was developed to accurately separate standards of **33** and 4-aminobenzoate (**35**). (b) Combination of TsaM-VanB with **33** reveals formation of the expected product, **35** (m/z = 136.0404). (c) The linear range of product formation was investigated, and it was determined that 5 min with 5 µM enzyme is an optimal time for running the assays. (d) Fitting of the data to the Michaelis-Menten equation revealed the apparent kinetic parameters for the reaction (see Table 1). In this figure, the data in panels c and d were measured using n = 2 independent experiments. Source data are provided as a Source Data file.

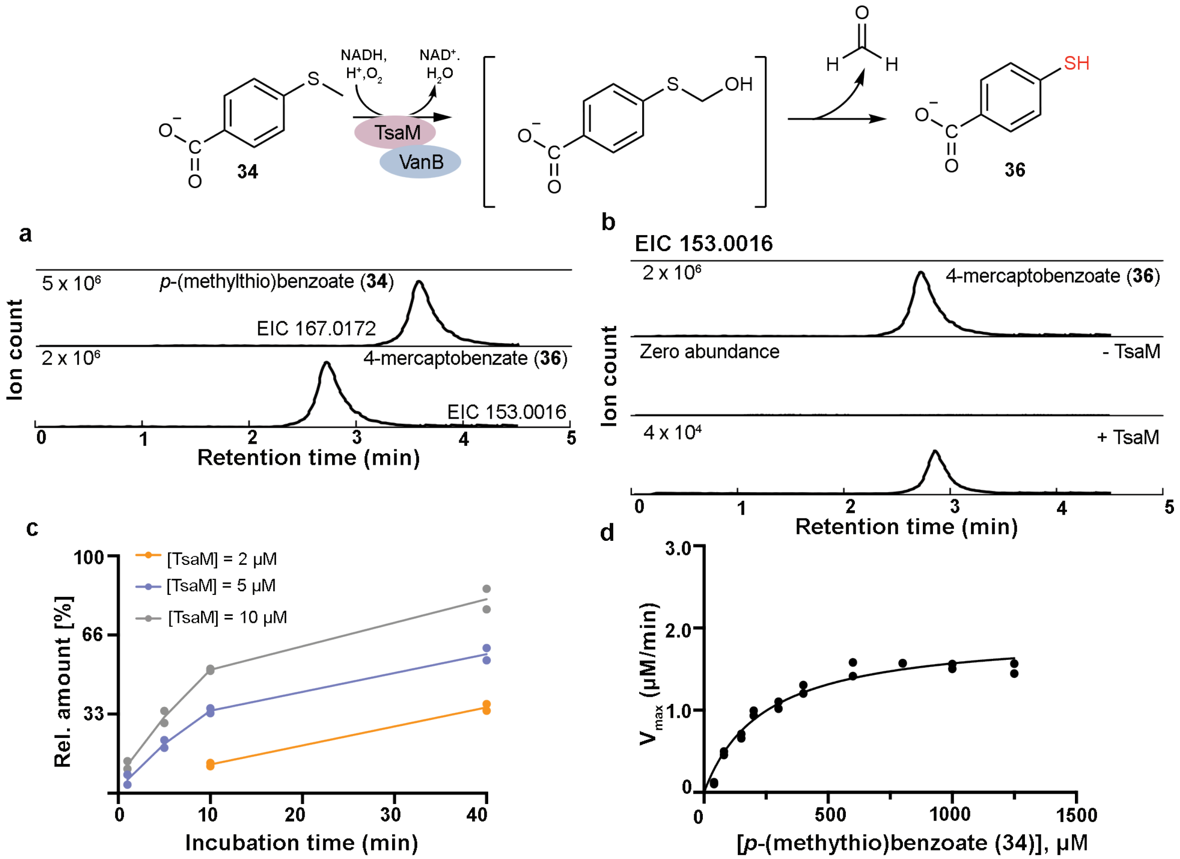

Supplementary Figure 33. The TsaM-VanB system also accepts a *p*-(methylthio)benzoate (**34**) substrate. (a) An LC-MS method was developed to accurately separate standards of **34** and 4-mercaptobenzoate (**36**). (b) Combination of TsaM-VanB with **34** reveals formation of the expected product (**36**, m/z = 153.0016). (c) The linear range of product formation was investigated, and it was determined that 5 min with 5 μM enzyme is an optimal time for running the assays. (d) Fitting of the data to the Michaelis-Menten equation revealed the apparent kinetic parameters for the reaction (see Table 1). In this figure, the data in panels c and d were measured using n = 2 independent experiments. Source data are provided as a Source Data file.

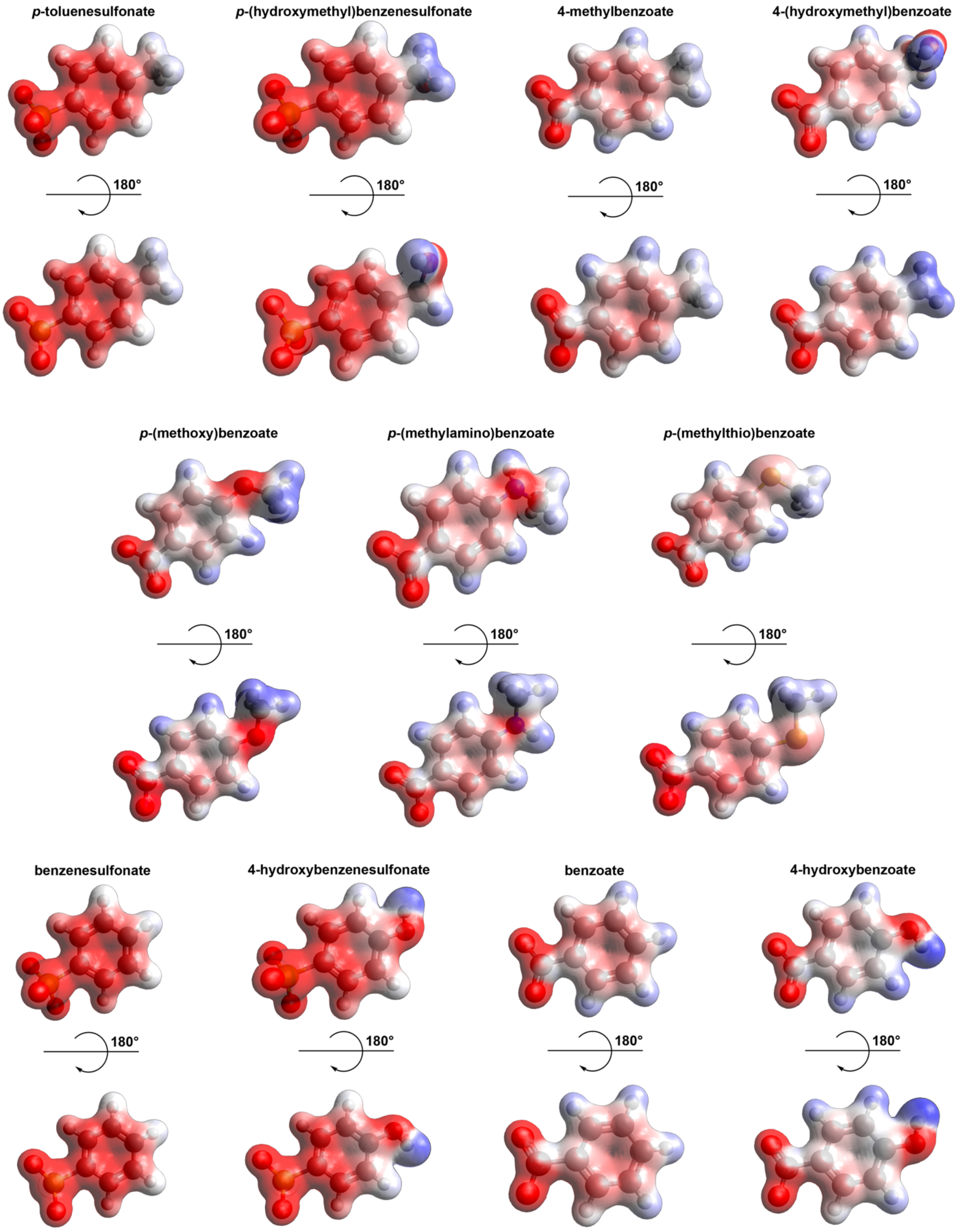
p-toluenesulfonate
p-(hydroxymethyl)benzenesulfonate
4-methylbenzoate
4-(hydroxymethyl)benzoate
180°
180°
180°
180°
p-(methoxy)benzoate
p-(methylamino)benzoate
p-(methylthio)benzoate
180°
180°
180°
benzenesulfonate
4-hydroxybenzenesulfonate
benzoate
4-hydroxybenzoate
180°
180°
180°
180°

Supplementary Figure 34. Density functional theory calculations performed on the substrates *p*-toluenesulfonate (**1**), 4-(hydroxymethyl)benzenesulfonate (**3**), 4-methylbenzoate (**2**), 4-(hydroxymethyl)benzoate (**4**), *p*-(methoxy)benzoate (**31**), *p*-(methylamino)benzoate (**33**), *p*-(methylthio)benzoate (**34**), benzoate (**38**), 4-hydroxybenzoate (**32**), benzenesulfonate (**37**), and 4-hydroxybenzenesulfonate (**41**) show differing partial charge distributions. This image shows that whereas in each substrate the charge on the carboxylate or sulfonate is maintained, different functional groups at the *p*-position create different charge distributions. For example, the molecules *p*-(hydroxymethyl)benzenesulfonate and 4-(hydroxymethyl)benzoate have regions of partial positive charge around the hydrogen atoms in the hydroxymethyl group and a region of partial negative charge around the oxygen atom in the hydroxymethyl group. The methoxy moiety of *p*-(methoxy)benzenoate introduces a region of partial negative charge at the *p*-position not present in *p*-toluenesulfonate and 4-methylbenzoate. Similarly, the methylamino group of *p*-(methylamino)benzoate introduces partial negative charge at the *p*-position and breaks the partial charge symmetry around the aromatic plane of the substrate. In *p*-(methylthio)benzoate, the methylthio functionality gives the molecule the most similar charge distribution to 4-methylbenzoate, but the inclusion of the methylthio group introduces steric bulk that is not present in *p*-toluenesulfonate and 4-methylbenzoate. Benzenesulfonate and benzoate are smaller than *p*-toluenesulfonate and 4-methylbenzoate, respectively, as both lack a functional group at the *p*-position. The hydroxy moiety of 4-hydroxybenzenesulfonate and 4-hydroxybenzoate bestows these two molecules with similar steric bulk at the *p*-position to that of 4-methylbenzoate and *p*-toluenesulfonate and further creates local charge distributions similar to the charge distributions present in *p*-(hydroxymethyl)benzenesulfonate and 4-(hydroxymethyl)benzoate. Partial charge distributions are visualized using the electrostatic potential surface around the molecule at 0.002 a.u. with positive charge in blue, neutral in white, and negative charge in red. Three species, *p*-toluenesulfonate, 4-methylbenzoate, and *p*-(methoxy)benzoate, were reported in our previous work[4] but were re-calculated here.

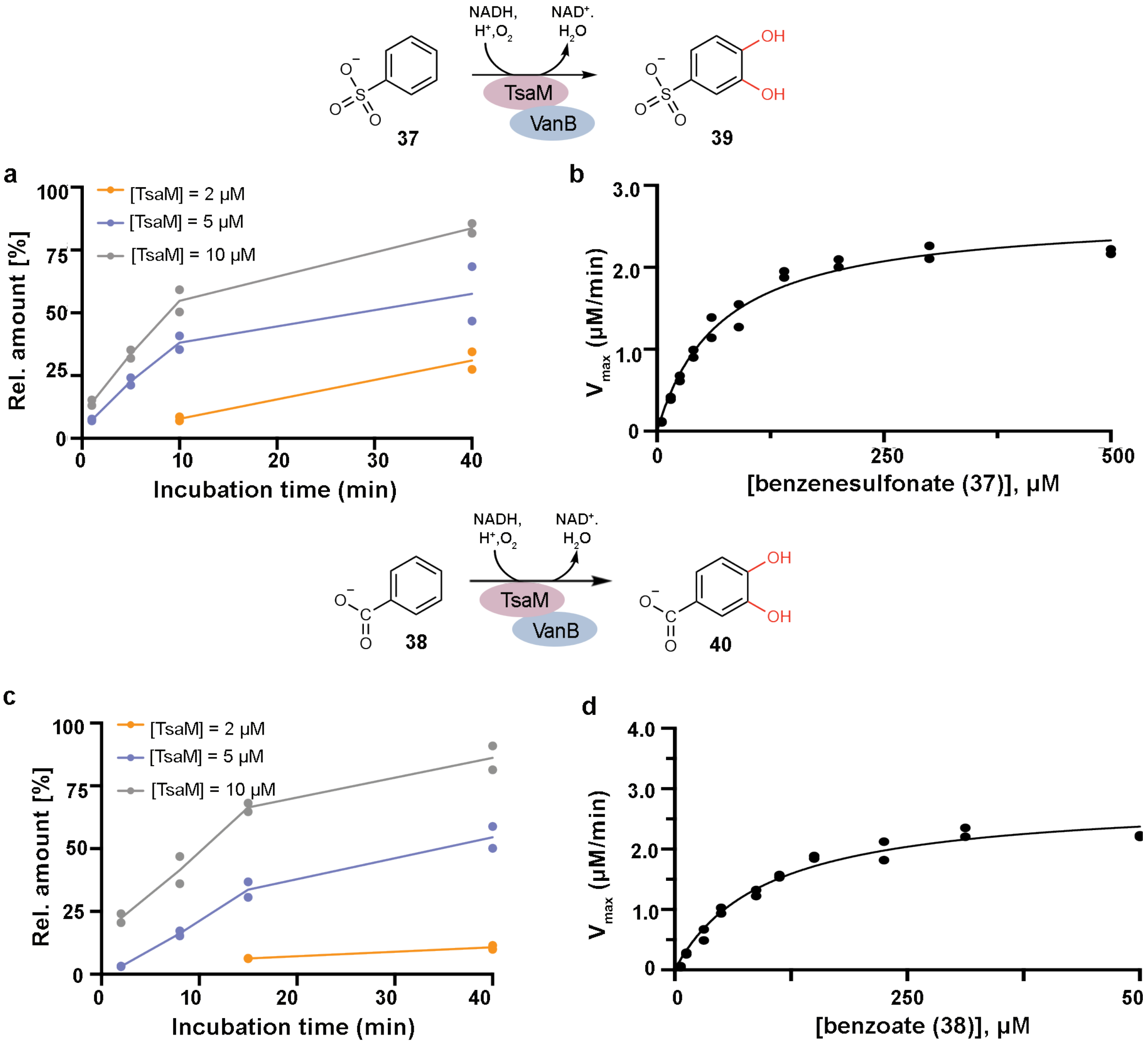

Supplementary Figure 35. The apparent kinetic parameters for the TsaM-VanB system were measured using benzenesulfonate (**37**) and benzoate (**38**) substrates. (a) The linear range for production of 3,4-dihydroxybenzene sulfonate (**39**) was investigated when **37** was provided as a substrate. This measurement suggested a time of 5 min with 5 µM TsaM is appropriate for the kinetic assay. (b) Fitting of the data to the Michaelis-Menten equation revealed the apparent kinetic parameters for the reaction (see Table 1). (c) The linear range for production of 3,4-dihydroxybenzoate (**40**) was investigated when **38** was provided as substrate. This measurement also suggested a time of 5 min with 5 µM TsaM is appropriate for the kinetic assay. (d) Fitting of the data to the Michaelis-Menten equation revealed the apparent kinetic parameters for the reaction (see Table 1). In this figure, the data were measured using n = 2 independent experiments. Source data are provided as a Source Data file.

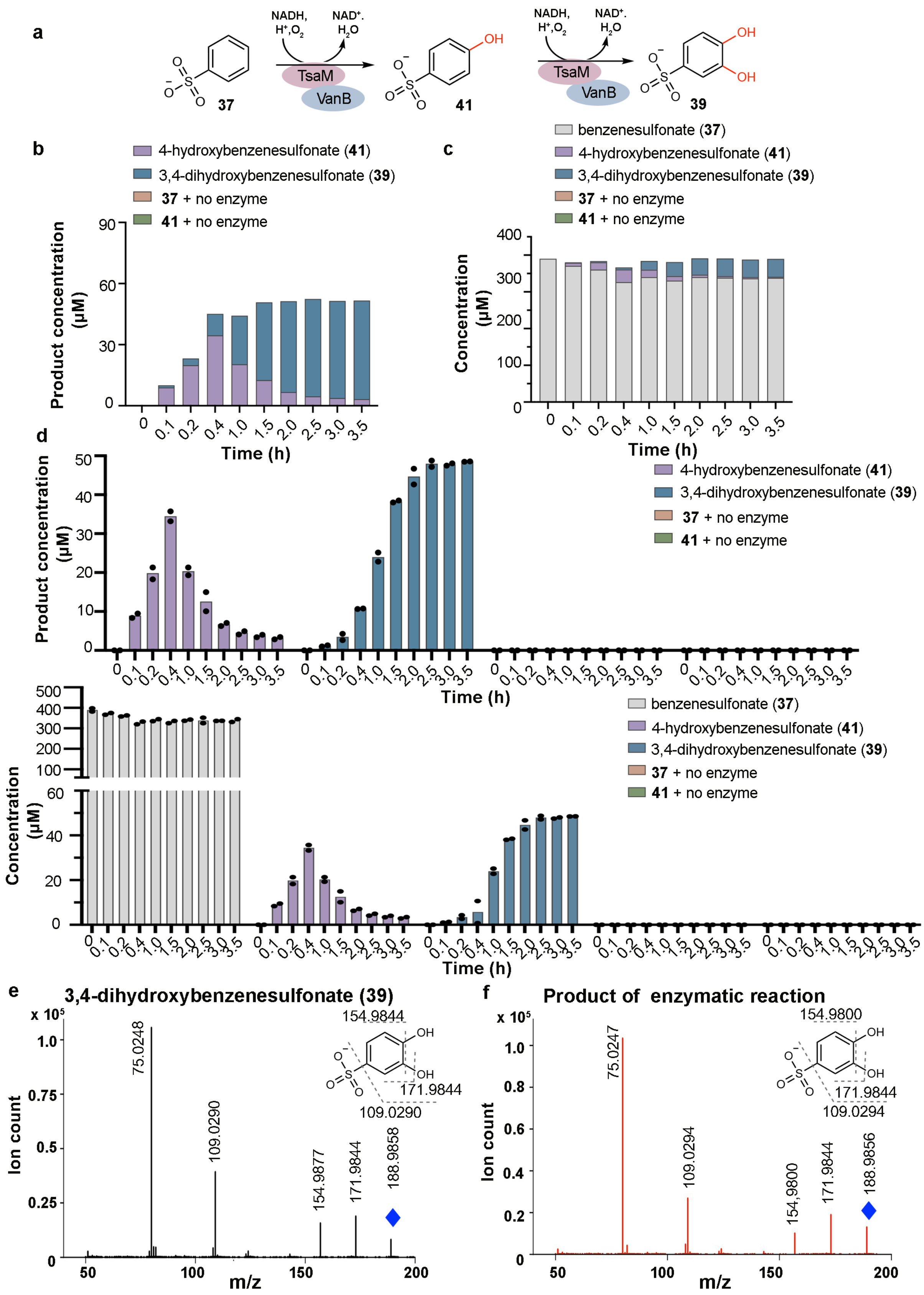
a
NADH, H+,O2
NAD+. H2O
TsaM
VanB
37
41
39
b
4-hydroxybenzenesulfonate (41)
3,4-dihydroxybenzenesulfonate (39)
37 + no enzyme
41 + no enzyme
Product concentration (μM)
Time (h)
c
benzenesulfonate (37)
Concentration (μM)
d
e
3,4-dihydroxybenzenesulfonate (39)
Ion count
m/z
f
Product of enzymatic reaction

Supplementary Figure 36. Time dependent assays on the TsaM-VanB system performed with benzenesulfonate (**37**) reveals formation of monooxygenated (4-hydroxybenzenesulfonate, **41**) and sequentially oxygenated (3,4-dihydroxybenzenesulfonate, **39**) products. (a) A general scheme of sequential oxygenation of **37**. (b) The ratio of mono- and dioxygenated products over time is consistent with sequential oxygenation. (c) A mass balance experiment reveals that the starting substrate amount is consistent with the cumulative mass of the mono- and dioxygenated products formed, indicating no unanticipated loss of substrate. (d) Data from panels b and c, now showing the total amounts of the substrate and product formed over time, and the control reactions that do not contain TsaM. For these experiments, the total amount of the products formed saturates, and the concentrations were calculated using an internal standard (see Supplementary Figure 73f). (e) The MS/MS fragmentation pattern of a 3,4-dihydroxybenzenesulfonate (**39**) product standard. (f) The MS/MS fragmentation pattern of the reaction product confirms the assignment of the product as **39**. In this figure, the data in panels b-d were measured using n = 2 independent experiments. Individual data points are represented in panel d, which corresponds to the data shown in panels b-c. Source data are provided as a Source Data file.

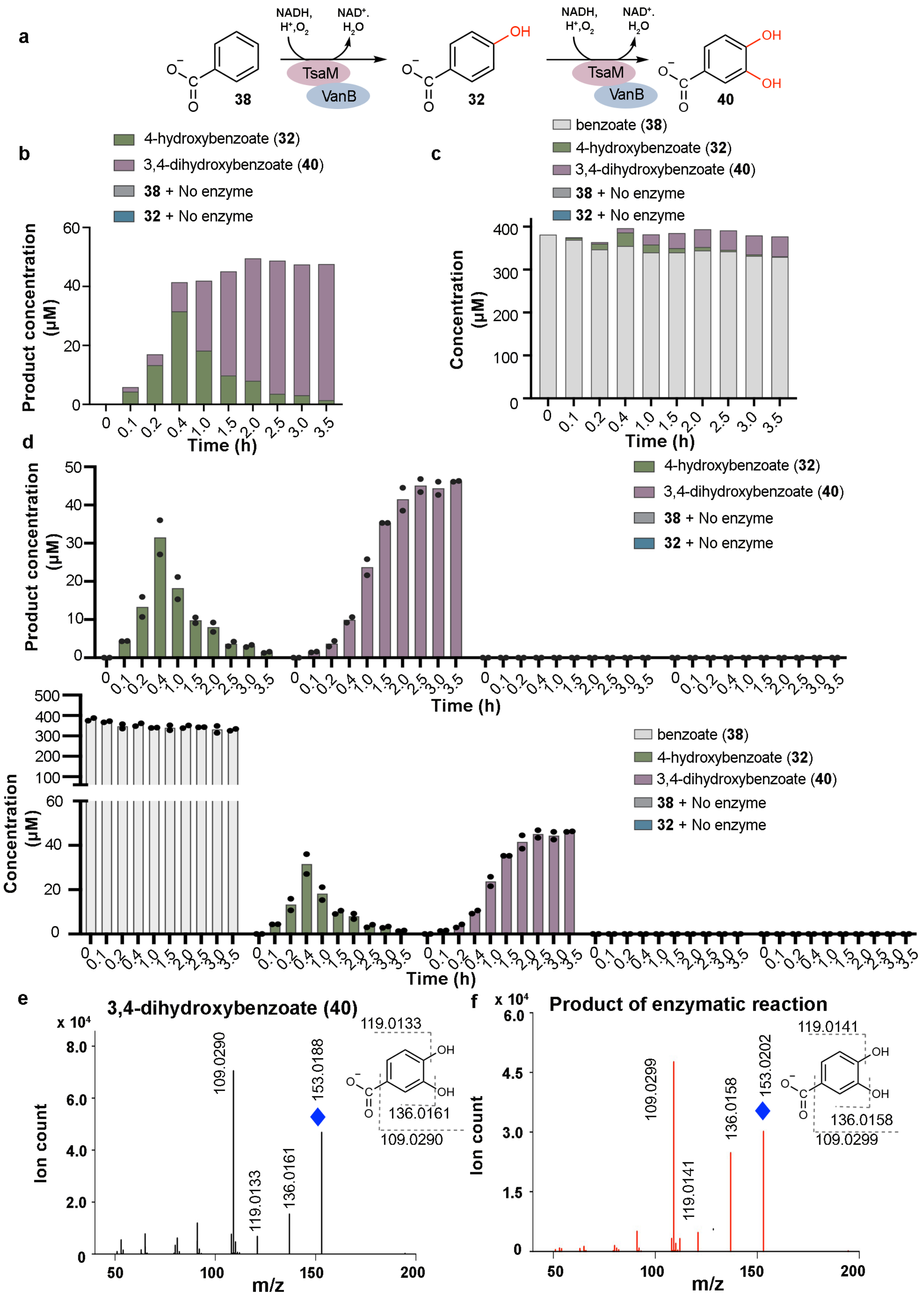
a
NADH, H+,O2
NAD+. H2O
TsaM
VanB
38
32
40
OH
b
4-hydroxybenzoate (32)
3,4-dihydroxybenzoate (40)
38 + No enzyme
32 + No enzyme
Product concentration (µM)
Time (h)
c
benzoate (38)
Concentration (µM)
d
e
3,4-dihydroxybenzoate (40)
Ion count
m/z
109.0290
119.0133
136.0161
153.0188
f
Product of enzymatic reaction
109.0299
119.0141
136.0158
153.0202

Supplementary Figure 37. Time dependent assays on the TsaM-VanB system performed with benzoate (**38**) reveals formation of monooxygenated (4-hydroxybenzoate, **32**) and sequentially oxygenated (3,4-dihydroxybenzoate, **40**) products. (a) A general scheme of sequential oxygenation of **38**. (b) The ratio of mono- and dioxygenated products over time is consistent with sequential oxygenation. (c) A mass balance experiment reveals that the starting substrate amount is consistent with the cumulative mass of the mono- and dioxygenated products, indicating no unanticipated loss of substrate. (d) Data from panels b and c, now showing the total amounts of the substrate and product formed over time, and the control reactions that do not contain TsaM. For these experiments, the total amount of the products formed saturates, and the concentrations were calculated using an internal standard (see Supplementary Figure 73f). (e) The MS/MS fragmentation pattern of a 3,4-dihydroxybenzoate (**40**) product standard. (f) The MS/MS fragmentation pattern of the reaction product confirms the assignment of the reaction product as **40**. In this figure, the data in panes b-d were measured using n = 2 independent experiments. Source data are provided as a Source Data file.

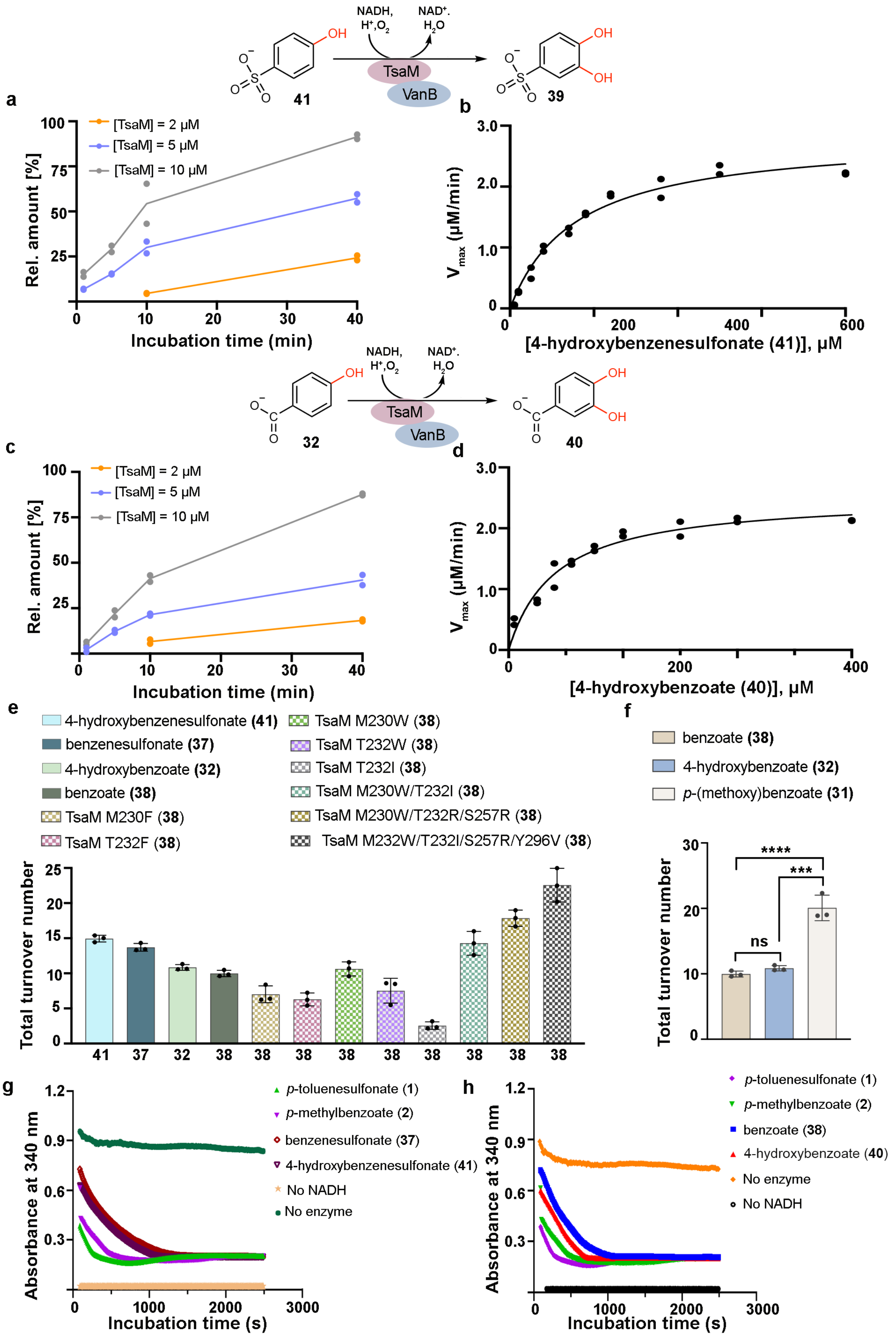

NADH, H+,O2
NAD+. H2O
TsaM
VanB
41
39
a
b
[TsaM] = 2 µM
[TsaM] = 5 µM
[TsaM] = 10 µM
Rel. amount [%]
Incubation time (min)
Vmax (µM/min)
[4-hydroxybenzenesulfonate (41)], µM
32
40
c
d
[4-hydroxybenzoate (40)], µM
e
f
4-hydroxybenzenesulfonate (41)
benzenesulfonate (37)
4-hydroxybenzoate (32)
benzoate (38)
TsaM M230F (38)
TsaM T232F (38)
TsaM M230W (38)
TsaM T232W (38)
TsaM T232I (38)
TsaM M230W/T232I (38)
TsaM M230W/T232R/S257R (38)
TsaM M232W/T232I/S257R/Y296V (38)
Total turnover number
benzoate (38)
4-hydroxybenzoate (32)
p-(methoxy)benzoate (31)
****
***
ns
g
h
p-toluenesulfonate (1)
p-methylbenzoate (2)
benzenesulfonate (37)
4-hydroxybenzenesulfonate (41)
No NADH
No enzyme
benzoate (38)
4-hydroxybenzoate (40)
Absorbance at 340 nm
Incubation time (s)

Supplementary Figure 38. The apparent kinetic parameters for the TsaM-VanB system were measured using the single monooxygenated intermediates 4-hydroxybenzenesulfonate (**41**) and 4-hydroxybenzoate (**32**). (a) The linear range for production of **39** was investigated when **41** was provided as a substrate. This measurement suggested a time of 5 min with 5 µM TsaM is appropriate for the kinetic analysis. (b) Fitting of the data on the Michaelis-Menten plot, to the Michaelis-Menten equation revelated the kinetic parameters for the reaction (see Table 1). (c) The linear range for production of **40** was investigated when **32** was provided as substrate. This measurement suggested a time of 5 min with 5 µM TsaM is appropriate for the kinetic assay. (d) Fitting of the data on the Michaelis-Menten plot, to the Michaelis-Menten equation revealed the kinetic parameters for the reaction (see Table 1). (e) The total turnover numbers (TTNs) are plotted for wild-type TsaM with each of the substrates **32**, **37**, **38**, and **41**, as well as for the TsaM variants that perform deoxygenation chemistry on benzoate. In this panel data was measured using n=3 independent experiments and is presented as mean values ± SD. (f) The TTNs are plotted for when **38**, **32**, and **31** are provided as a substrate to wild-type TsaM. In this panel **** indicates p < 0.0005, *** indicates p < 0.0001 using an ANOVA one way Tukey analysis. Here the calculated p values are 0.1241, 0.0004, and <0.0001. In this panel data was measured using n=3 independent experiments and is presented as mean values ± SD. (g) NADH consumption was monitored at 340 nm in reactions that contained TsaM, VanB, and either **1**, **2**, **37**, or **41**. (h) NADH consumption was monitored at 340 nm in reactions that contained TsaM, VanB, and either **1**, **2**, **38**, or **40**. In this figure, the data for all panels were measured using n = 2 (a-d) independent experiments. Source data are provided as a Source Data file.

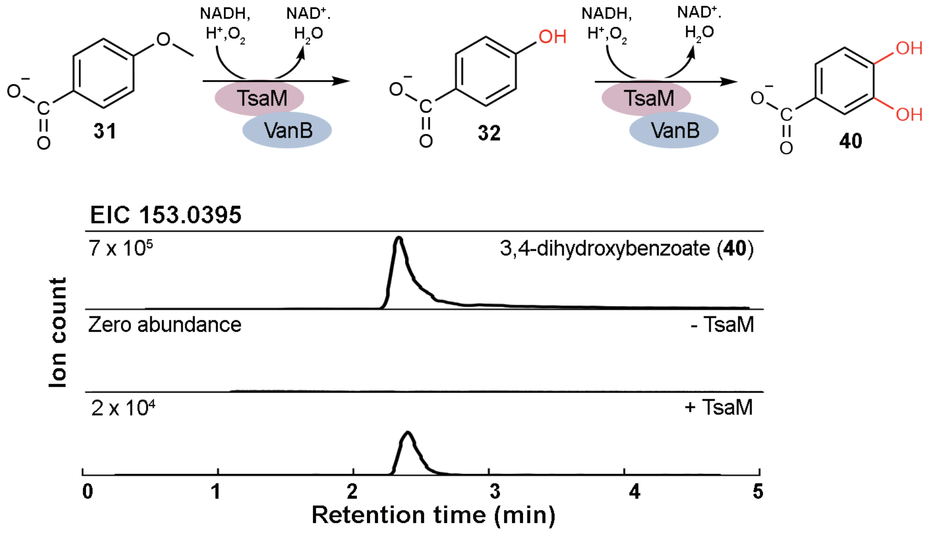

Supplementary Figure 39. When *p*-(methoxy)benzoate (**31**) is used as a substate of the TsaM-VanB system, formation of a dealkylation product, 4-hydroxybenzoate (**32**), is observed. Similarly, as **32** is another substrate of TsaM, the generation of **40** is also detected.

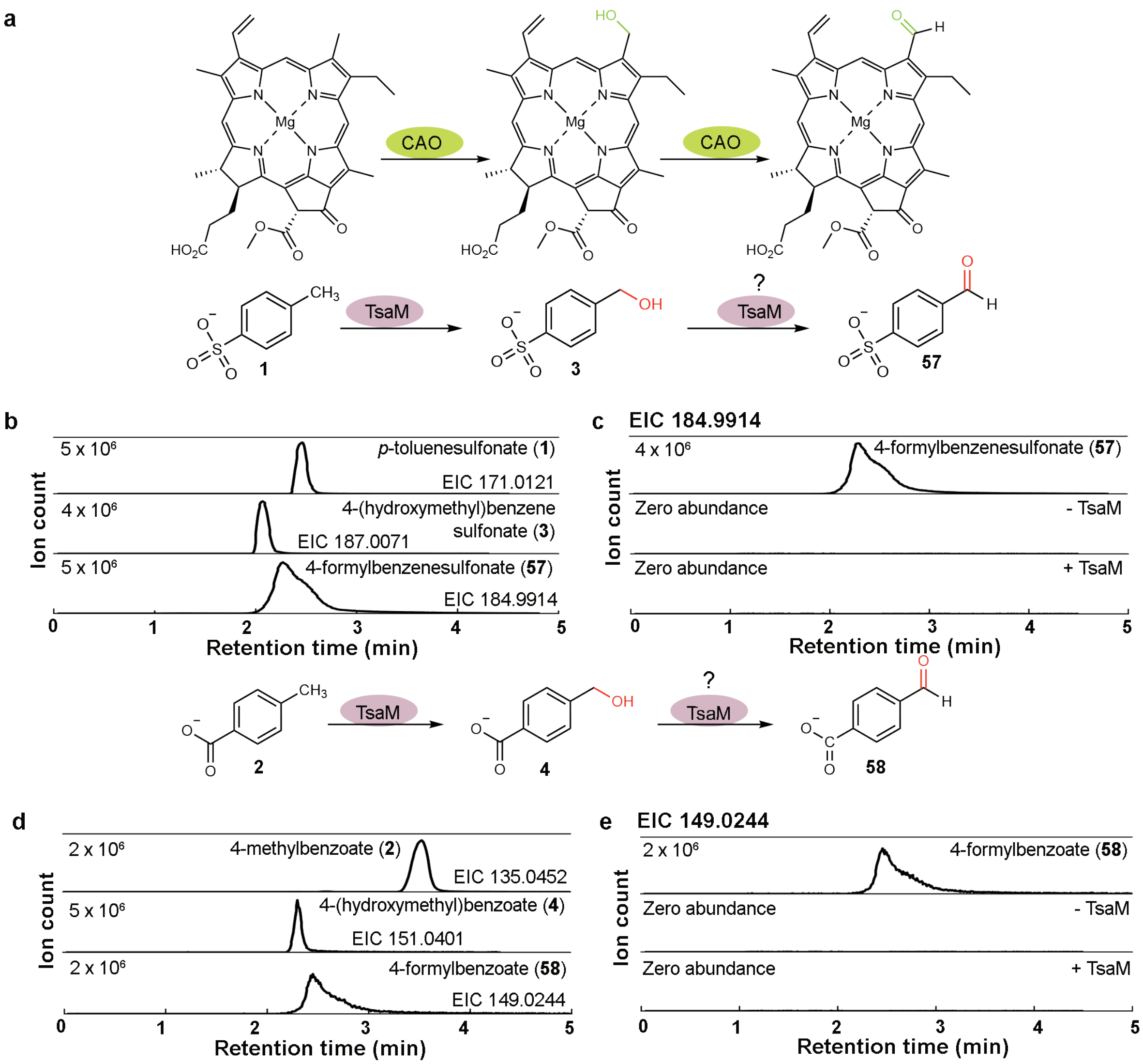

Supplementary Figure 40. TsaM, unlike CAO, does not catalyze sequential oxygenation reactions on its native substrate. (a) CAO catalyzes sequential monooxygenation reactions that convert a methyl group at the C7 position of chlorophyllide *a* into a formyl group[8]. The reaction catalyzed by CAO raised the question of whether TsaM could also catalyze sequential monooxygenation reactions on its native substrate. (b) Capitalizing on our methods to separate the substrate (**1**) from the monooxygenated product (**3**)[4], LC-MS was employed to also separate the potential formylated product (4-formylbenzenesulfonate, **57**). (c) The ability of the TsaM-VanB system to generate the formylated product was tested using the established LC-MS method. The extracted ion chromatograms from this experiment reveal that the TsaM-VanB system is unable to produce a formylated product (m/z = 184.9914), indicating that TsaM does not natively catalyze sequential monooxygenation reactions. (d) A previously described LC-MS method to separate the substrate (**2**) from its monooxygenated product (**4**)[4] was harnessed to also separate the formylated product (4-formylbenzoate, **58**). (e) The exacted ion chromatogram indicates that no formylated product is generated when **2** is provided as a substrate.

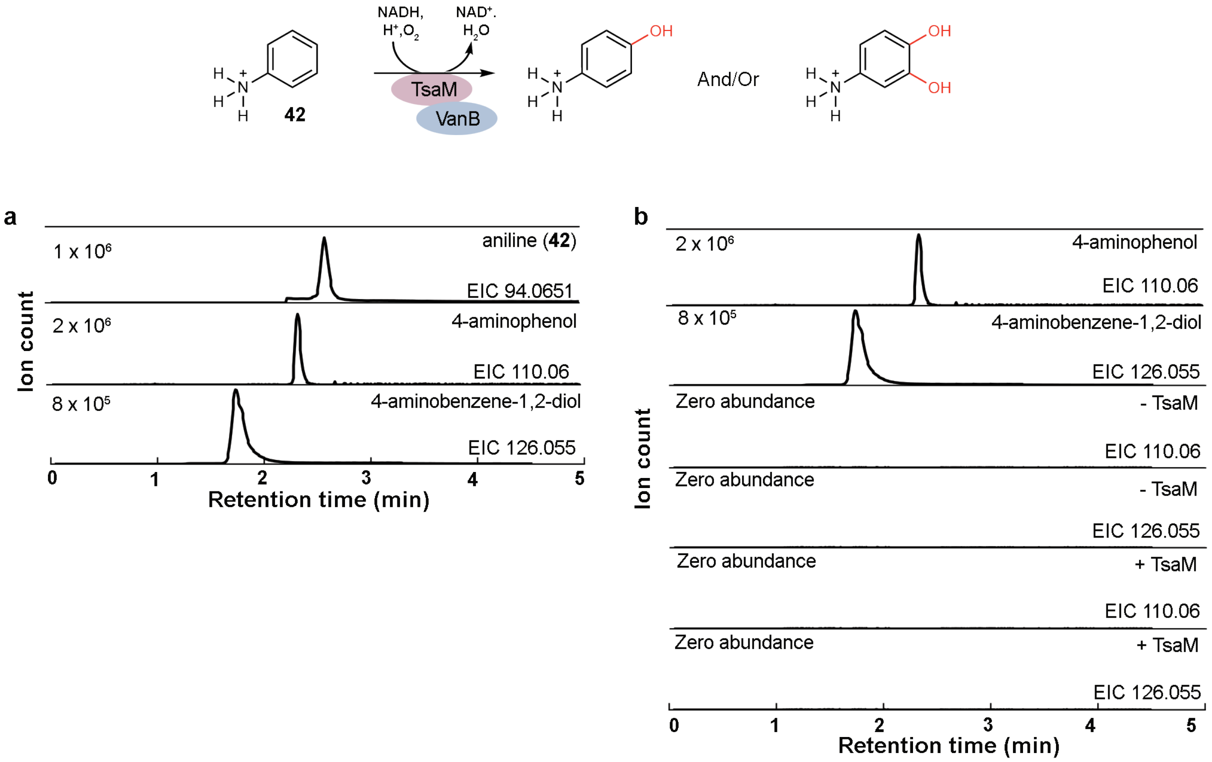

Supplementary Figure 41. Combination of the TsaM-VanB system with an aniline (**42**) substrate does not result in formation of an oxygenated product. (a) LC-MS was used to separate **42** from each of the expected products. (b) Analysis of the reaction that contained TsaM-VanB and **42** shows the absence of any mono- or dioxygenated products.

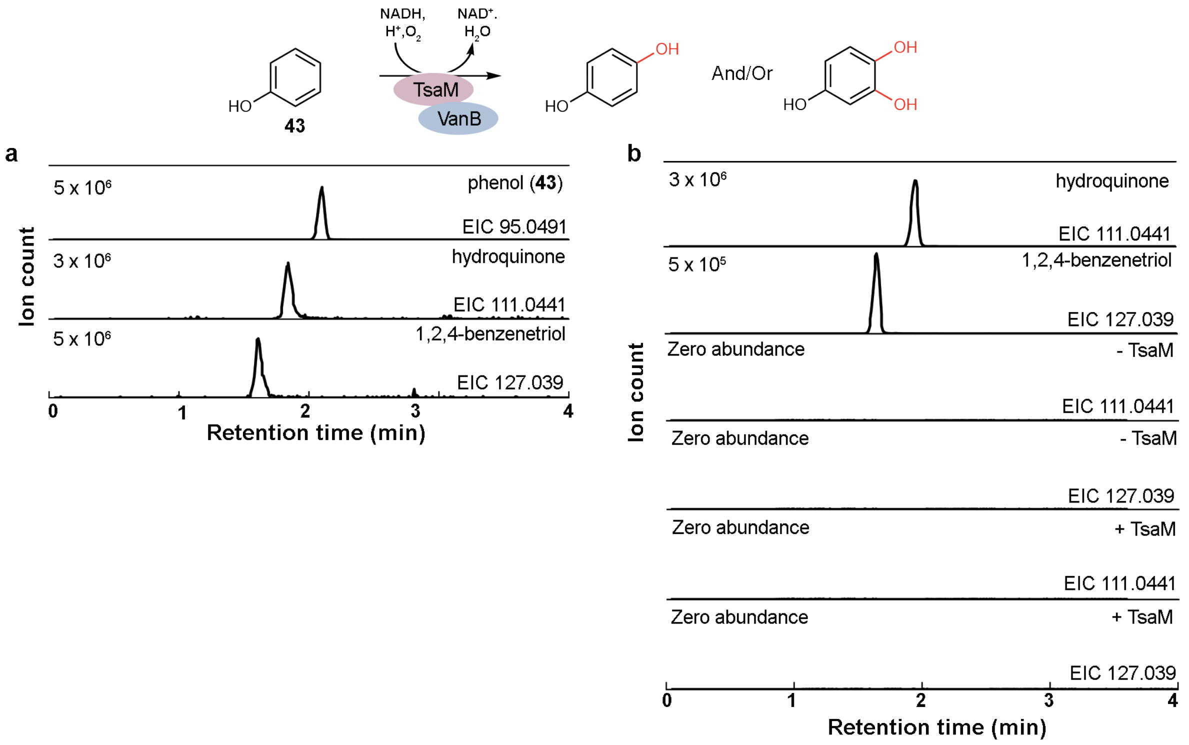

Supplementary Figure 42. Combination of the TsaM-VanB system with a phenol (**43**) substrate does not result in formation of an oxygenated product. (a) LC-MS was used to separate **43** from each of the expected products. (b) Analysis of the reaction that contained TsaM-VanB and **43** shows the absence of any mono- or dioxygenated products.

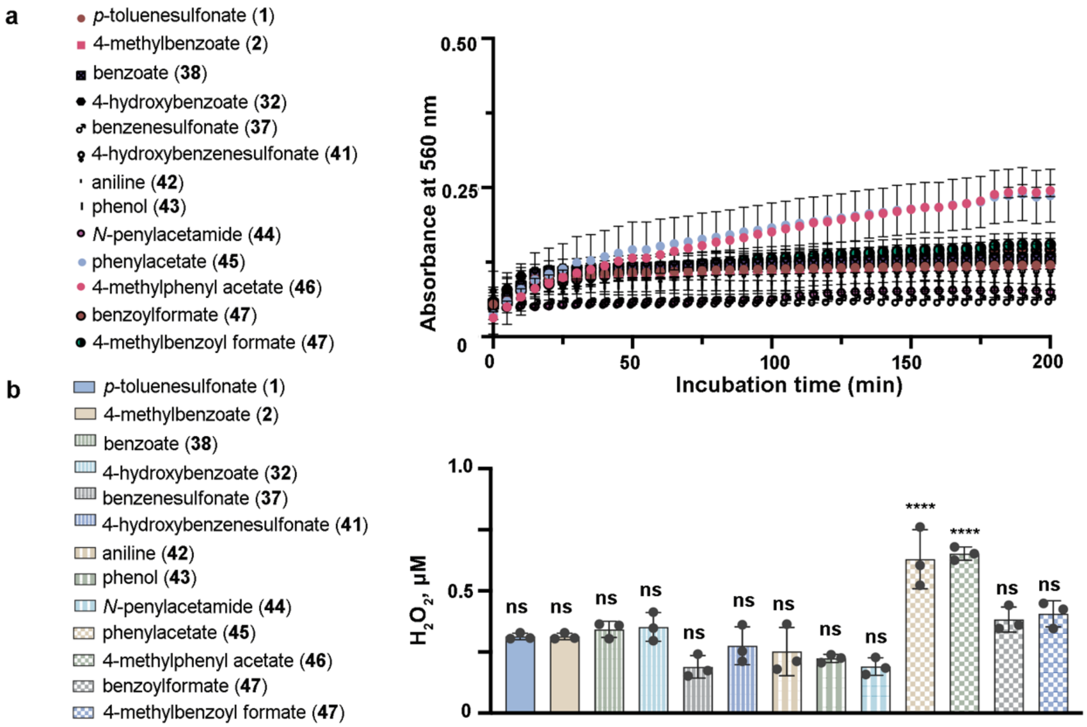

Supplementary Figure 43: An uncoupling assay reveals that supplying phenylacetate (**45**) or 4-methylphenyl acetate (**46**) as a substrate to the TsaM-VanB system leads to O_2 uncoupling. (a) The absorbance at 560 nm was measured for the TsaM-VanB system with several different substrates over a 3 h incubation. (b) The amount of uncoupling was determined by comparison to a reaction that contained TsaM-VanB and either *p*-toluenesulfonate or *p*-methylbenzoate and the absorbance signal from panel a. The conversion of the absorbance signal at 560 nm to H_2O_2 concentration was calculated using previously described methods and a previously calculated standard curve[4]. This analysis reveals that incubation of TsaM-VanB with either phenylacetate (**45**) or 4-methylphenyl acetate (**46**) leads to a significant amount of uncoupling. As these substrates are not detectably oxygenated by TsaM, the only observed activity is unproductive activation. This result contrasts that observed for aniline (**42**), phenol (**43**), and *N*-phenylacetamide (**44**) for which there is no observed hydroxylation nor uncoupling. The uncoupling result is also unlike that observed with benzoylformate (**47**) and 4-methylbenzoylformate (**48**) substrates, which support either mono- or sequential monooxygenation activity of TsaM-VanB. In this panel ****p < 0.0001 and ns indicates no significant difference from an ordinary one-way ANOVA Tukey analysis. P values from left to right >0.9999, 0.9987, 0.9855, 0.1146, 0.9878, 0.8038, 0.4078, 0.1199, <0.0001, <0.0001, 0.6962, and 0.3664. In this figure, all the data were measured in n = 3 independent experiments and are presented as the mean value ± SD of these measurements. Source data are provided as a Source Data file.

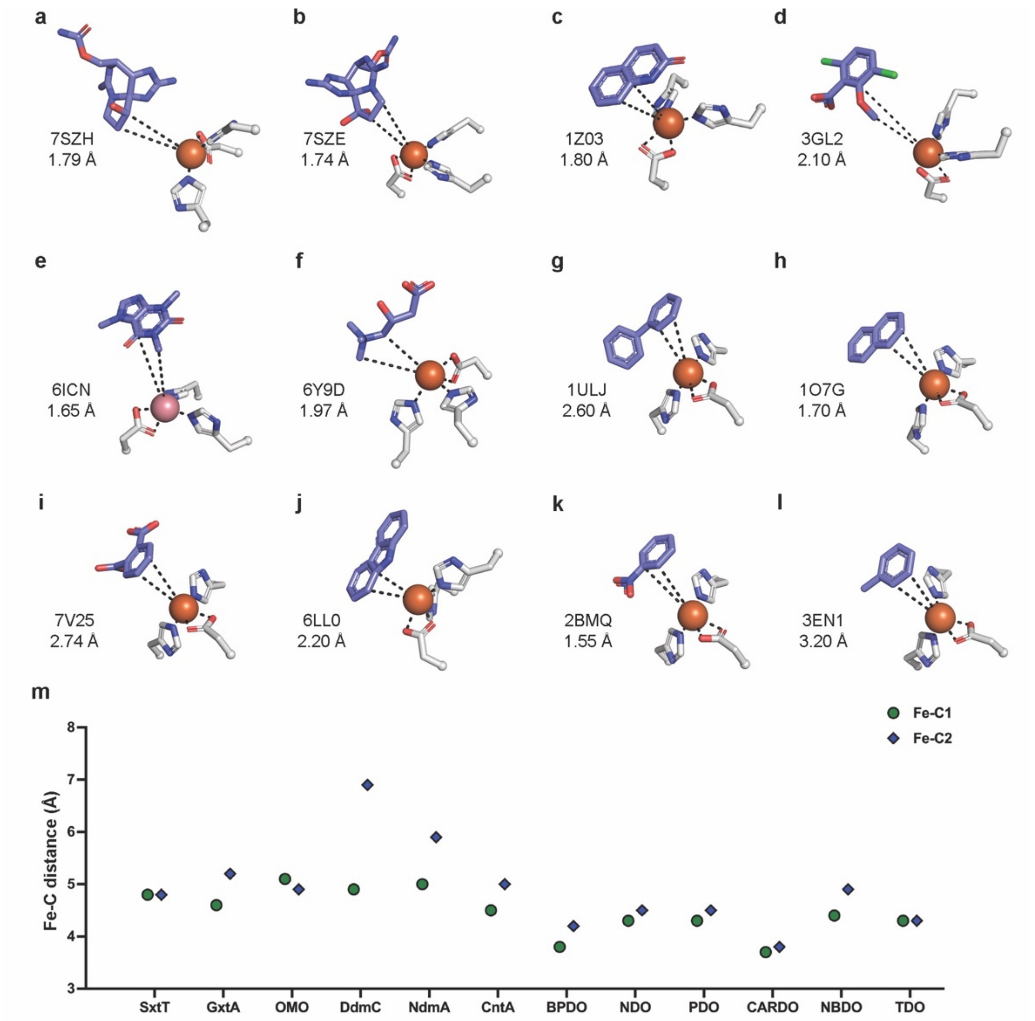

Supplementary Figure 44. The substrate bound Rieske oxygenase structures reveal that the distance between the substrate and mononuclear iron center may have a profound effect on reactivity. The PDB code and X-ray crystal structure resolution are given for each structure. The structures of (a) SxtT with β-saxitoxinol bound (PDB: 7SZH)[9], (b) GxtA with saxitoxin bound (PDB: 7SZE)[9], (c) 2-oxoquinoline 8-monooxygenase (OMO) with 2-oxoquinoline bound (PDB: 1Z03)[10], (d) dicamba monooxygenase (DdmC) with dicamba bound (PDB: 3GL2)[5], (e) NdmA with caffeine bound (PDB: 6ICN)[11], (f) CntA with L-carnitine bound (PDB: 6Y9D)[12], (g) biphenyl dioxygenase (BPDO) with biphenyl bound (PDB: 1ULJ)[13], (h) naphthalene 1,2 dioxygenase (NDO) with naphthalene bound (PDB: 1O7G)[14], (i) phthalate dioxygenase (PDO) with phthalate bound (PDB: 7V25)[15], (j) carbazole 1,9a-dioxygenase (CARDO) with carbazole bound (PDB: 6LL0), (k) nitrobenzene dioxygenase (NBDO) with nitrobenzene bound (PDB: 2BMQ)[16], and (l) toluene 2,3-dioxygenase (TDO) with toluene bound (PDB: 3EN1)[17] were used to measure the distance between the Fe center and the two closest substrate carbon atoms. (m) A plot of the distance between the first (green) and second (blue) closest carbon atoms of the substrate to the mononuclear iron center in the monooxygenase (left) and dioxygenase structures (right). Distances are visually shown in panels a through l; the shortest distances were chosen if multiple mononuclear iron centers with substrate bound were present in the solved structures. An unequal variances *t*-test shows that the distance distributions of the oxidized substrate carbon atom in the monooxygenases and the closest oxidized substrate carbon atom in the dioxygenases have significantly different means ($p = 0.0015$). Similarly, an unequal variances *t*-test shows that the distance distribution of the next closest substrate carbon atom in the monooxygenases and the second oxidized substrate carbon atom in the dioxygenases have significantly different means ($p = 0.0207$).

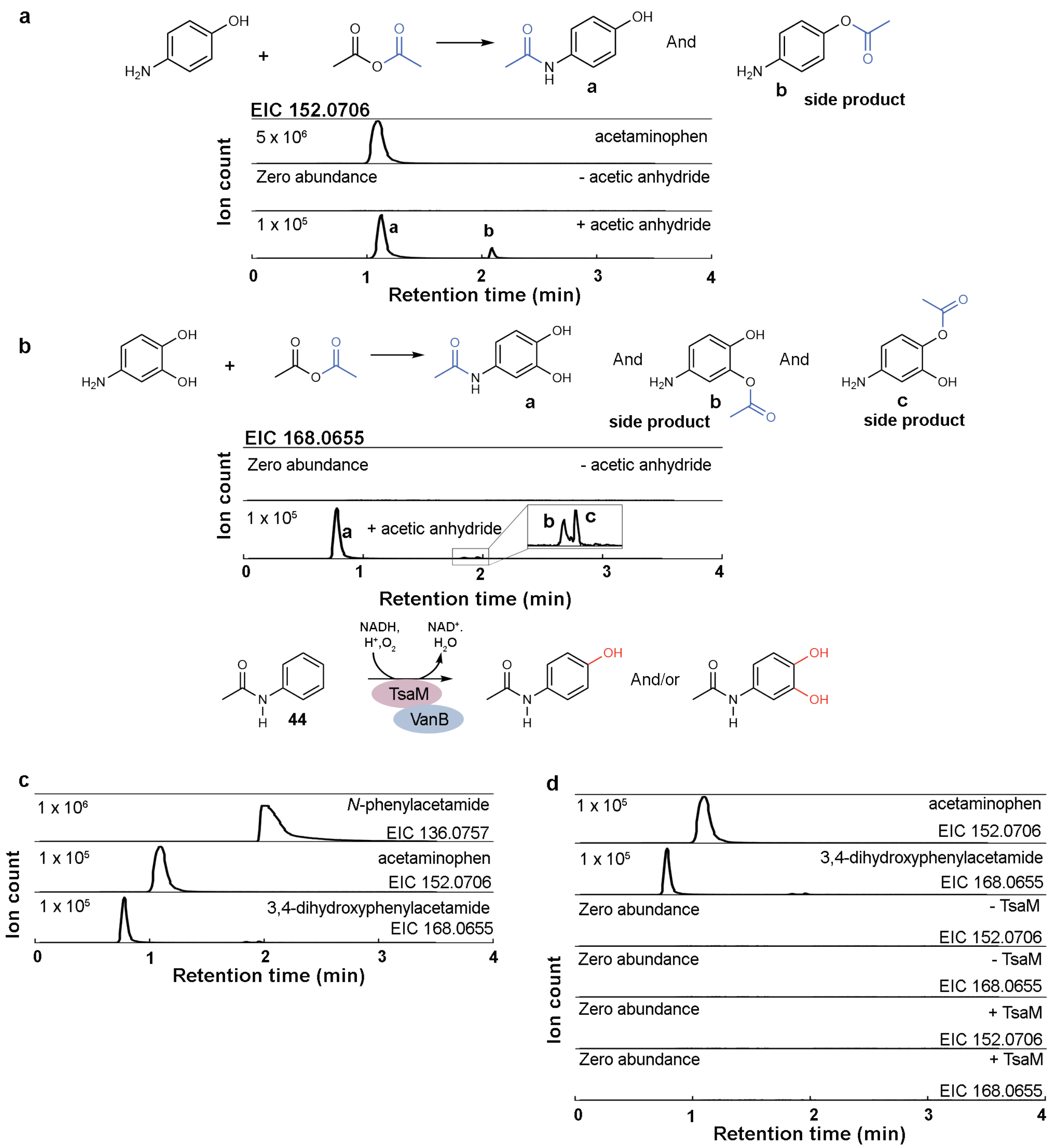

Supplementary Figure 45. Combination of the TsaM-VanB system with a *N*-phenylacetamide substrate (**44**) does not result in formation of an oxygenated product. (a) To produce the dioxygenated product standard, a synthetic method was developed. In this method, a monooxygenated product is first produced by mixing 4-aminophenol and acetic anhydride. The extracted ion chromatogram indicates that the main synthesis product (a) corresponds to the product standard (m/z = 152.0706). (b) A dioxygenated (a) product standard was synthesized following the protocol described in panel a. This method also resulted in production of minor side products (b and c). (c) LC-MS was used to separate **44** from each of the expected products that were synthesized in panels a and b. (d) Analysis of the reaction that contained TsaM-VanB and **44** showed the absence of any mono- or dioxygenated product species.

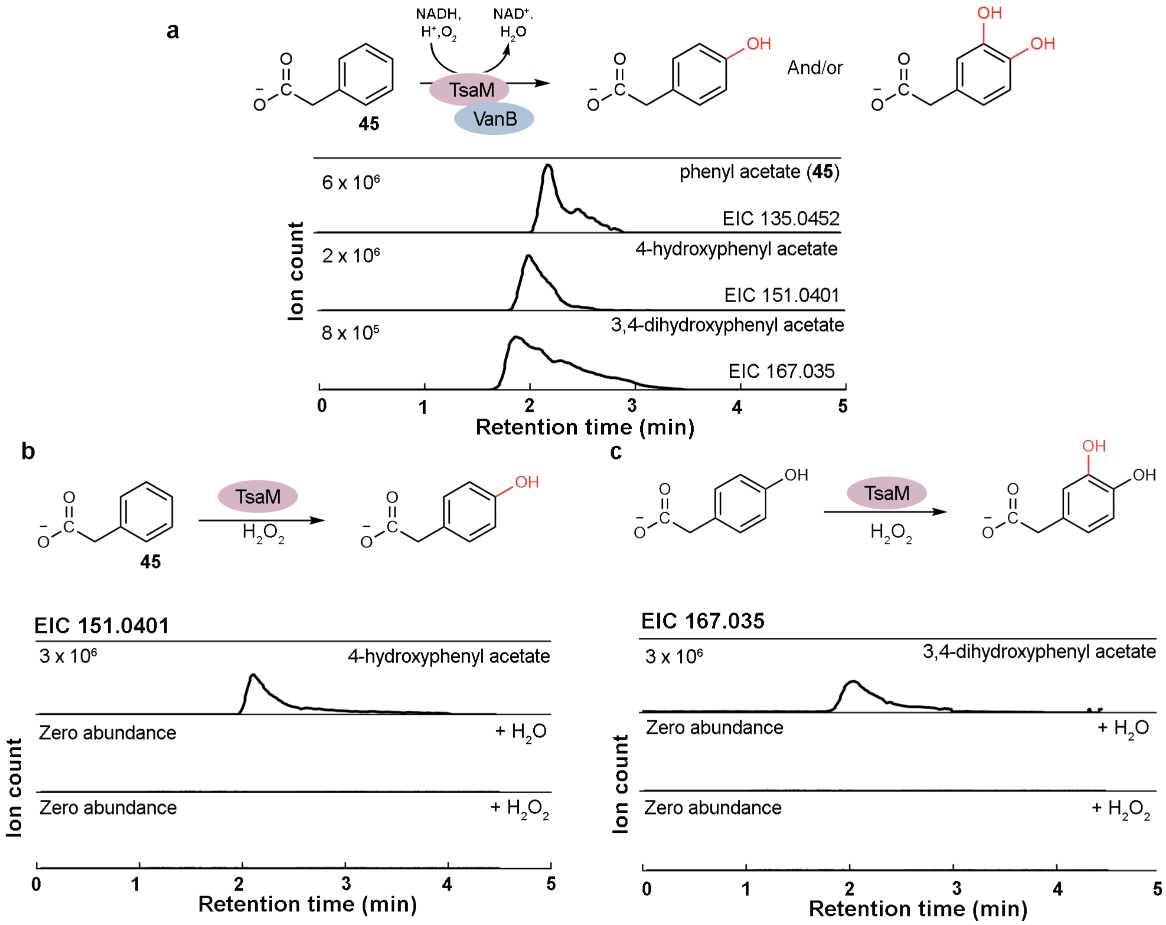

Supplementary Figure 46. The effect of changing the size of the substrate provided to the TsaM-VanB system was tested. (a) LC-MS can separate phenylacetate (**45**) from the proposed monooxygenated and dioxygenated products. (b) Providing **45** as a substrate to the TsaM-VanB system does not result in formation of a product using the H_2O_2 shunt reaction. (c) Similarly, providing a monooxygenated compound to the TsaM-VanB system as a substrate does not result in product formation, suggesting that these substrates either do not bind to TsaM or cannot be oxygenated by TsaM.

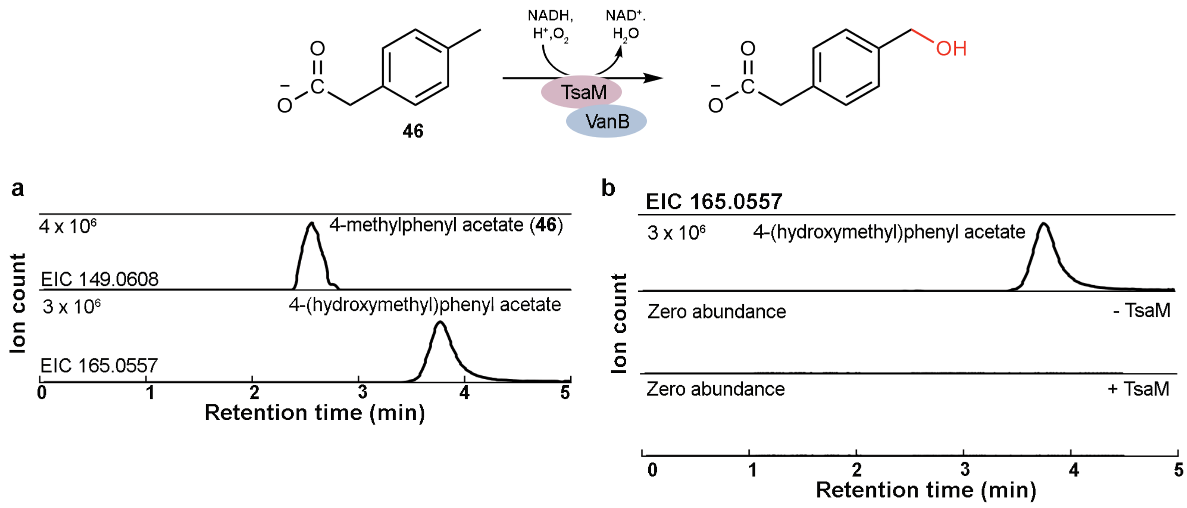

Supplementary Figure 47. Combination of the TsaM-VanB system with a 4-methylphenylacetate (**46**) substrate does not result in formation of an oxygenated product. (a) LC-MS was used to separate **46** from the proposed reaction product. (b) Analysis of the reaction that contained TsaM-VanB and **46** showed the absence of a molecule that matched a product standard.

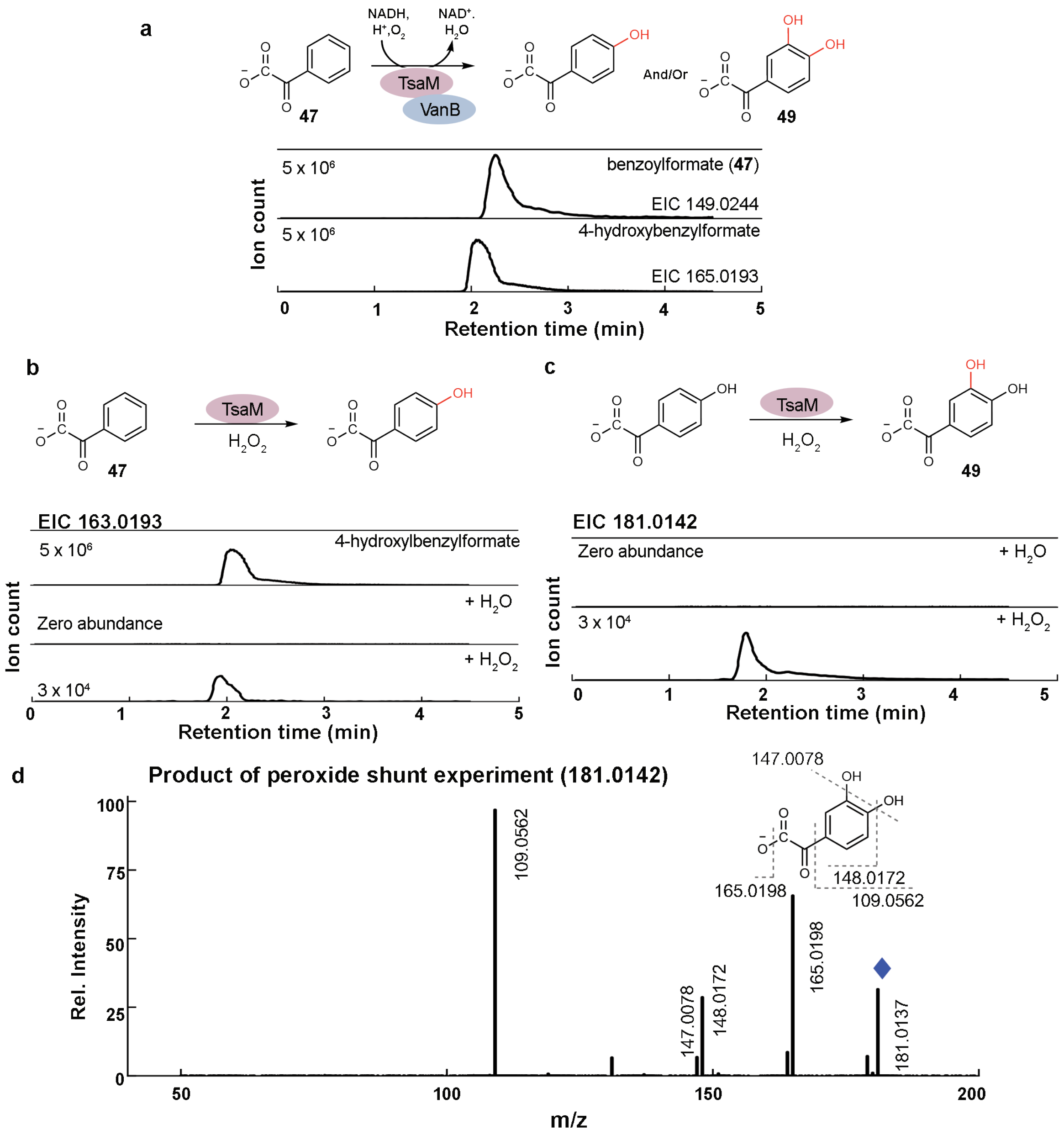

Supplementary Figure 48. The effect of changing the size of the substrate provided to the TsaM-VanB system was probed using a benzoylformate (**47**) substrate. (a) An LC-MS method was used to separate (**47**) from the proposed monooxygenated products. The dioxygenated product standard is not commercially available. (b) Providing **47** as a substrate to the TsaM-VanB system results in formation of a monooxygenated product using the H_2O_2 shunt reaction. (c) Similarly, providing the monooxygenated compound to the TsaM-VanB system as a substrate also results in formation of a dioxygenated (3,4-dihydroxybenzoylformate, (**49**) product. (d) MS/MS of the dioxygenated product was performed. The fragmentation pattern of the dioxygenated product indicates that it is the compound of interest. This data suggests that this larger substrate is sequentially oxygenated by TsaM.

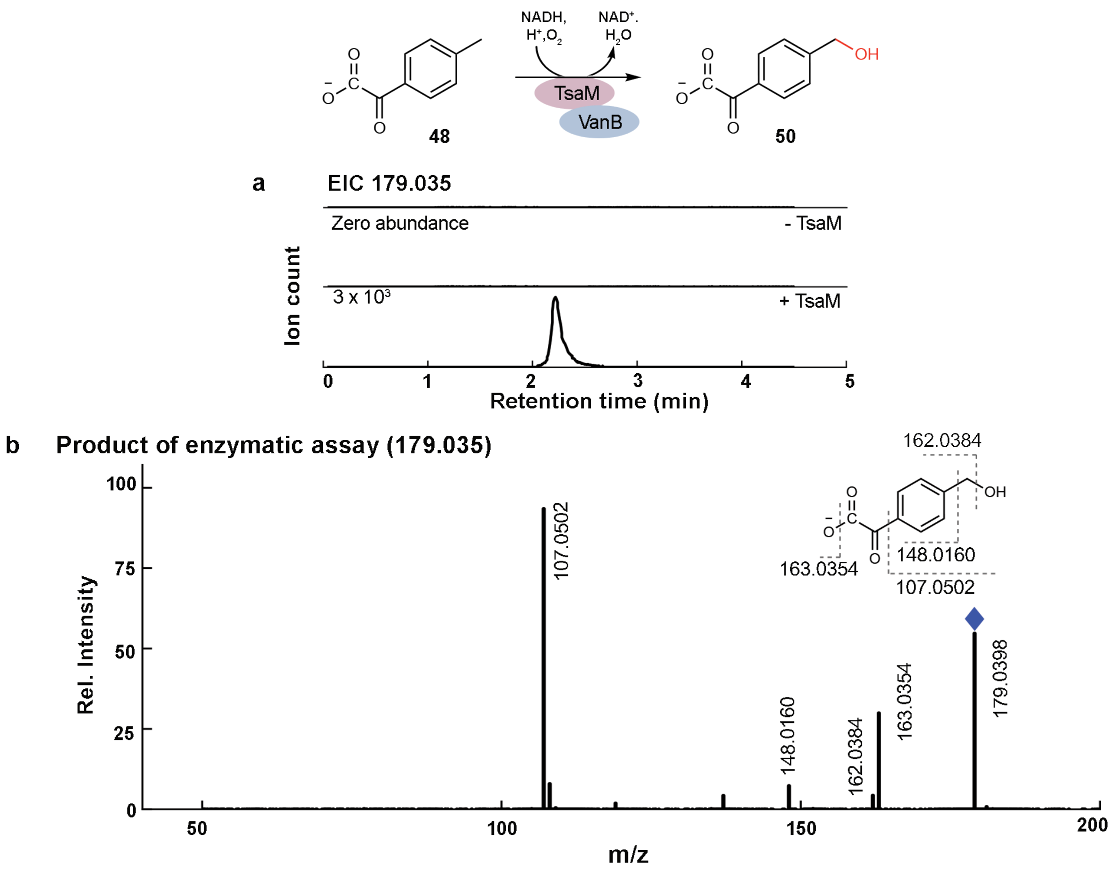

Supplementary Figure 49. TsaM is active on the larger 4-methylbenzoylformate (**48**) substrate. (a) An enzymatic assay that combines the TsaM-VanB system with **48** reveals the formation of a monooxygenated product (4-hydroxymethylbenzoylformate, **50**). (b) As a standard of **50** is not commercially available, MS/MS was used to show that this assay resulted in formation of an oxygenated product.

a

```
TsaM      --MFIRNCWYVAAWDTEIPAEGLFHRTLLNEPVLLYRDTQGRVVALENRCCHRSAPLHIG      58
DdmC      MATFVRNAWYVAALPEELSE-KPLGRTILDTPLALYRQPDGVVAALLDICPHRFAPLSDG      59

TsaM      RQEGDCVRCLYHGLKFNPSGACVEIPGQE-QIPPKTCIKSYPVVERNRLVWIWMGDPARA      117
DdmC      ILVNGHLQCPYHGLEFDGGGQCVHNPHGNGARPASLNVRSFPVVERDALIWIWPGDPALA      119

TsaM      NPDDIVDYFWHDSPEWRMKPGYIHYQANYKLIVDNLLDFTHLAWVHPTTLGTDSAASLKP      177
DdmC      DPGAIPDFGCRVDPAYRTVGGYGHVDCNYKLLVDNLMDLGHAQYVHRANAQTDAFDRLER      179

TsaM      VIERDTTGTGKLTITRWYLNDDMS-NLHKGVAKFEGKADRWQIYQWSPPALLRMDTGSAP      236
DdmC      EV---IVGDGEIQALMKIPGGTPSVLMAKFLRGANTPVDAWNDIRWNKVSAMLNFIAV--      234

TsaM      TGTGAPEGRRVPEAVQFRHTSIQTPETETTSHYWFCQARNFDLDDEALTEKIYQGVVVA-      295
DdmC      ----APEGTPKEQSIHSRGTHILTPETEASCHYFFGSSRNFGIDDPEMDGVLRSWQAQAL      290

TsaM      FEEDRTMIEAHEKILSQVPD---RPMVPIAADAGLNQGRWLLDRLLKAENGGTAP-----      347
DdmC      VKEDKVVVEAIERRRAYVEANGIRPAMLSCDEAAVRVSR-EIEKLEQLEAARLEHHHHHH      349
```

b

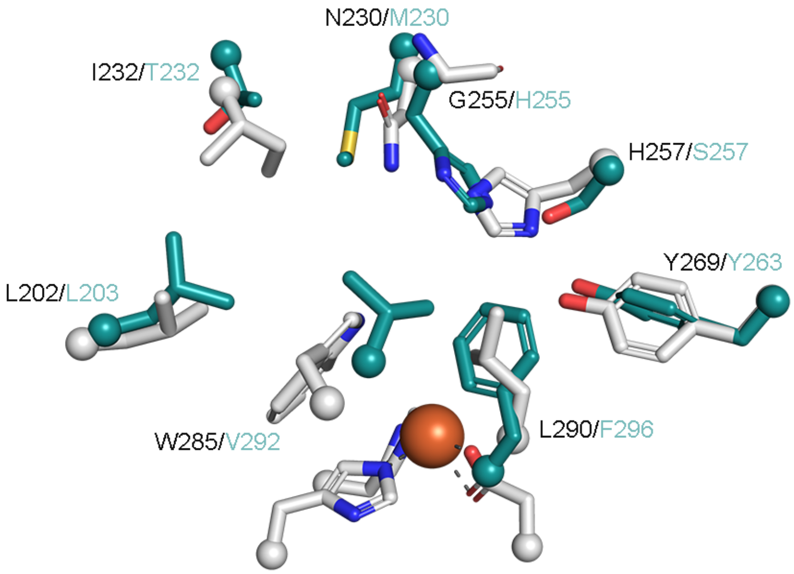

Supplementary Figure 50. A sequence alignment, previous information regarding important active site residues[4], and AlphaFold[6,7] model were used to identify residues that could be mutated to move the substrate closer to the mononuclear iron center in TsaM. (a) A sequence alignment of TsaM and DdmC. The residues involved in ligating the Rieske cluster and mononuclear iron center are highlighted in green and orange, respectively. The residues highlighted in the text are blue. (b) An AlphaFold model of TsaM (teal) was generated as previously described[4] and is overlaid with the active site residues of DdmC (PDB: 3GL2[5], gray) shows that the spatial arrangement of residues in TsaM may be slightly different than indicated in the sequence alignment. It also highlighted the possible importance of residues at the 230 and 232 positions to substrate orientation in the active site. These structures were visualized and analyzed using Pymol 2.5.2_93 software.

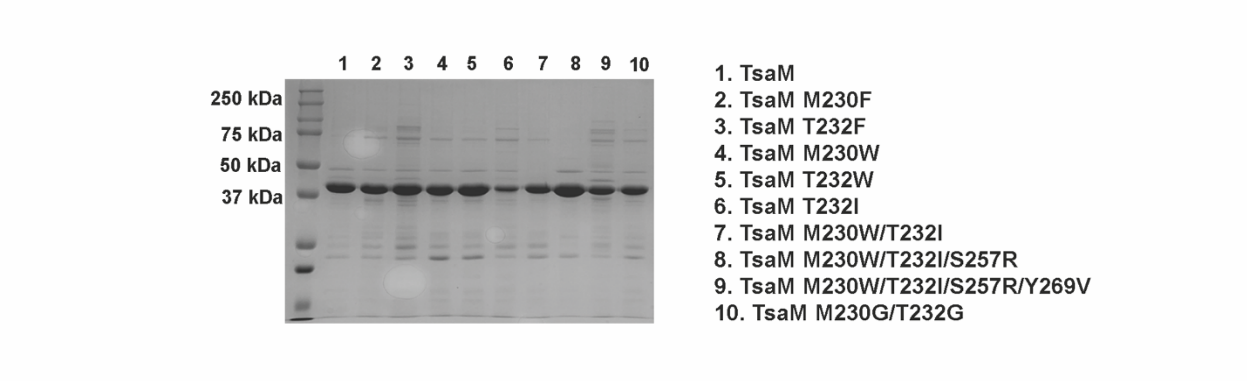

Supplementary Figure 51. The His-tagged wild-type TsaM and His-tagged TsaM variants used in this work were expressed and purified using previously described methods[4]. The purity of each protein was assessed using SDS-PAGE. Wild-type TsaM and all TsaM variants have a molecular weight of approximately 38 kDa. These variant proteins were purified and SDS-PAGE assessed approximately twice throughout the duration of this work with similar results.

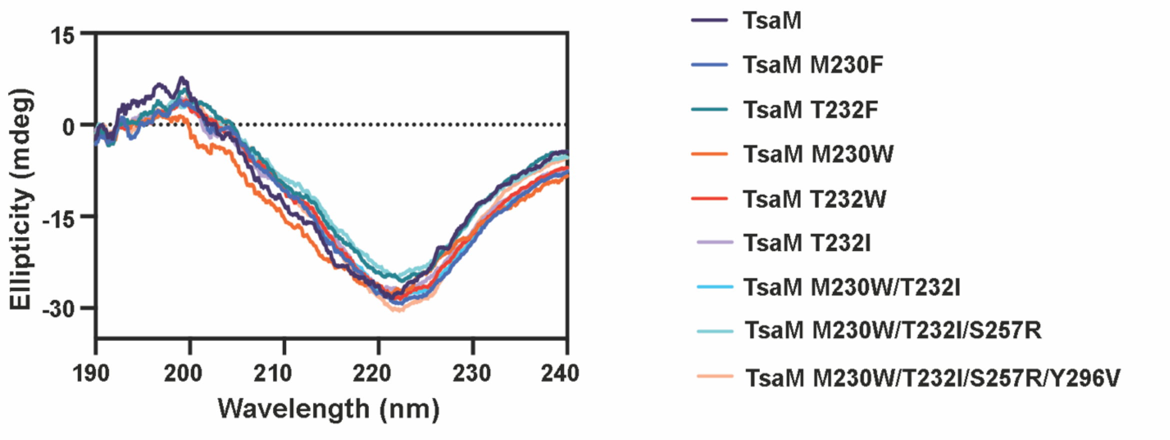

Supplementary Figure 52. Circular dichroism (CD) data of wild-type TsaM and all variants used in this work are similar, which suggests that despite different activity levels, all proteins are similarly folded. These experiments were performed using previously described methods[4]. Source data are provided as a Source Data file.

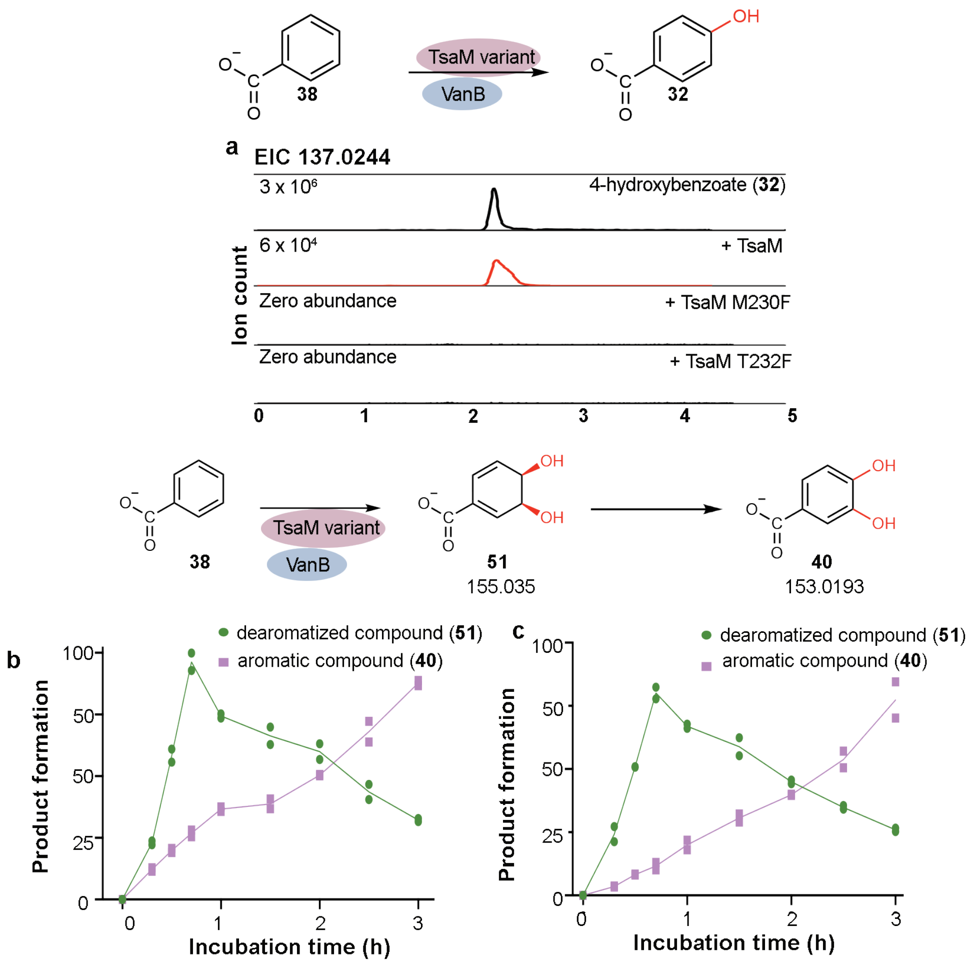

Supplementary Figure 53. The combination of the M230F and T232F TsaM variants, VanB, and **38** results in the formation of a *cis*-diol ((3*R*,4*S*)-3,4-dihydroxycyclohexa-1,5-diene-1-carboxylate, **51**). (a) Unlike wild-type TsaM, the M230F and T232F TsaM variants do not catalyze a single monoxygenation reaction on **38** to form **32.** (b) Instead, in the assays that contain the M230F variant. a dearomatized *cis*-diol product and a second dioxygenated species are formed in the assays. This aromatic species appears over time as **51** disappears. (c) The same phenomenon described in panel b is also observed with the T232F TsaM variant. In this figure, the data in panels b and c were measured using n = 2 independent experiments**.** Source data are provided as a Source Data file.

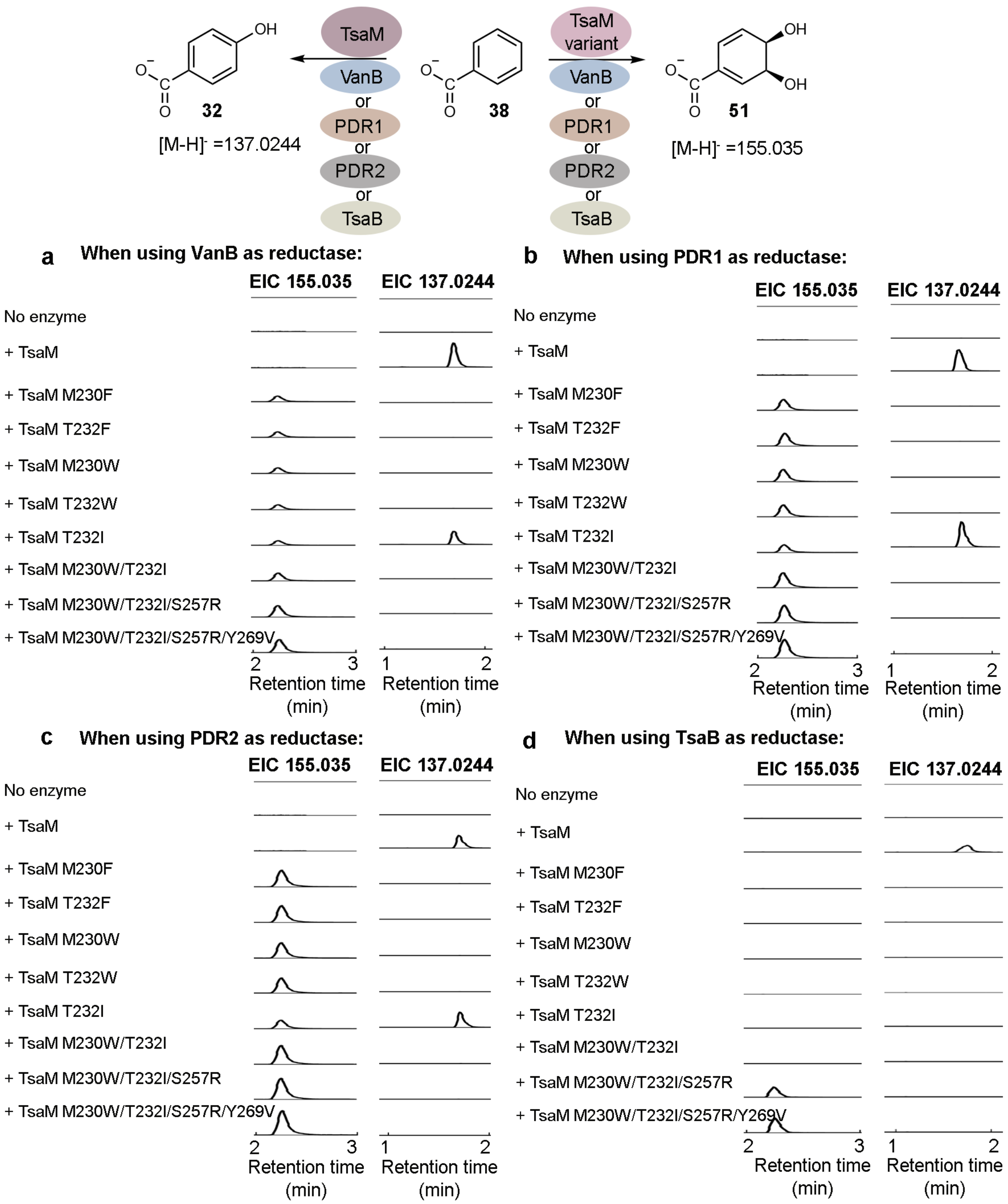

Supplementary Figure 54. TsaM M230F/W, T232F/W, M230W/T232I, M230W/T232I/S257R, or M230W/T232I/S257R/Y269V variants produce a dearomatized *cis*-diol product (**51**) when combined with benzoate (**38**), one of several different partner reductase proteins, such as (a) VanB, (b) PDR1, (c) PDR2, or (d) TsaB. This data also shows that TsaM variant T232I produces a mixture of 4-hydroxybenzoate (**32**) and **51**.

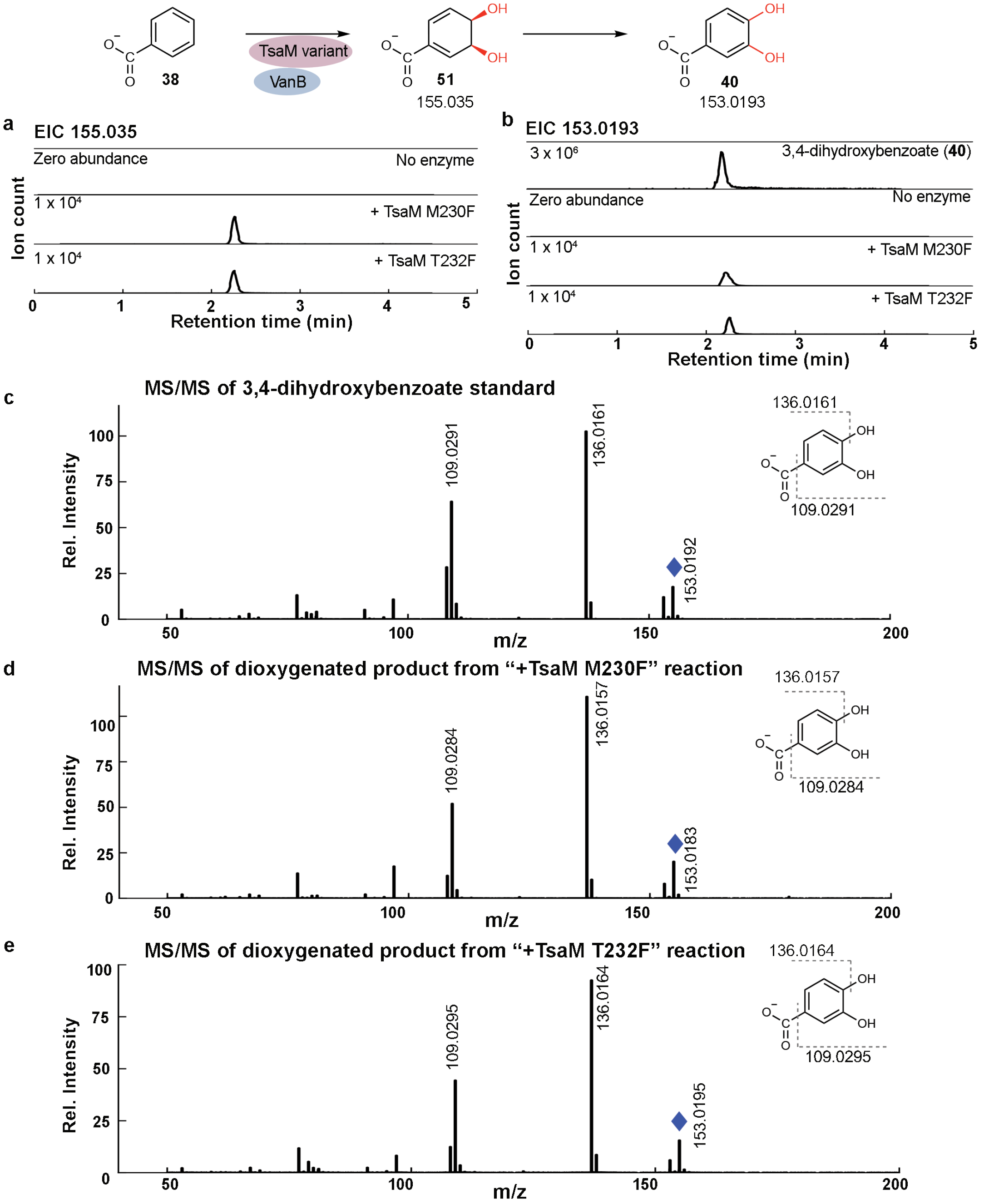

Supplementary Figure 55. The TsaM M230F and T232F variants produce a *cis*-diol ((3*R*,4*S*)-3,4-dihydroxycyclohexa-1,5-diene-1-carboxylate, **51**) when provided with a benzoate (**38**) substrate. (a) Incubation of M230F TsaM with **38** results in formation of **51,** which can be visualized using LC-MS. (b) The product of the M230F TsaM variant with **38** is converted into an aromatic species (**40**) over time. (c) The identity of the aromatic product was verified by comparison of the MS/MS fragmentation of a product standard. (d) The MS/MS fragmentation pattern of the product from the TsaM M230F variant reaction matches that shown in panel c. (e) The MS/MS fragmentation pattern of the product from the TsaM T232F variant reaction also matches that shown in panel c.

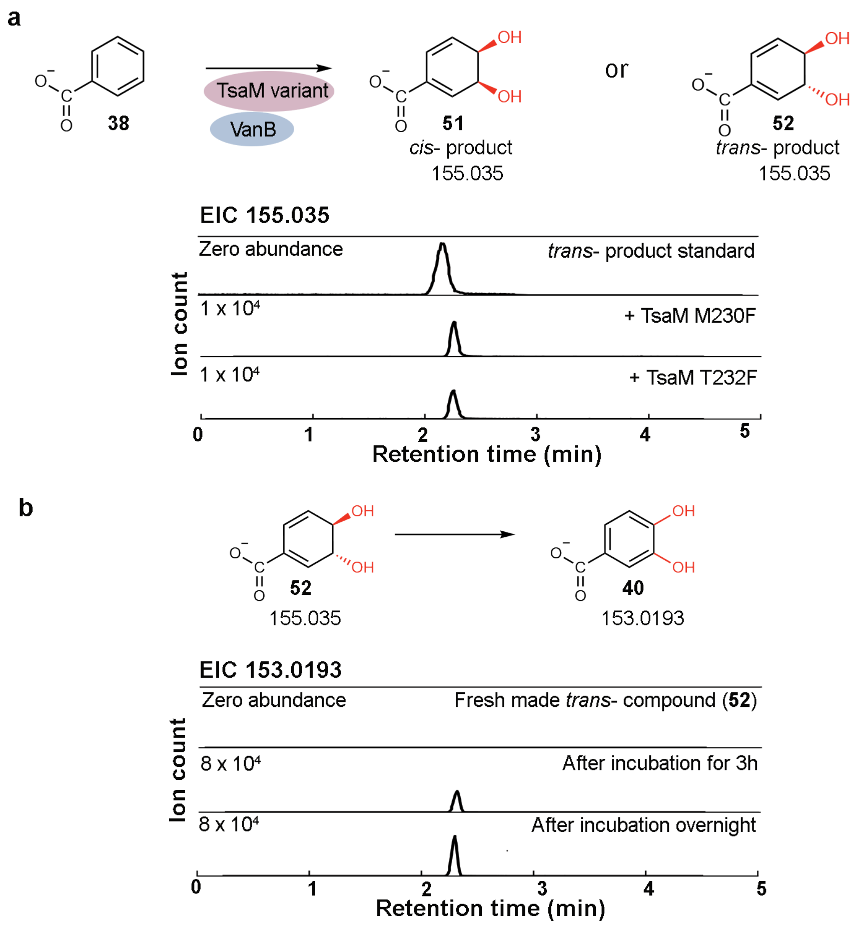

Supplementary Figure 56. The combination of the M230F or T232F TsaM variants, VanB, and **38** results in the formation of a *cis*-diol (**51**), the identify of which was verified in multiple ways. (a) To verify the identity of the dioxygenated product, a standard of (3*R*,4*R*)-3,4-dihydroxycyclohexa-1,5-diene-1-carboxylate (**52,** *trans*-product) was purchased. This species has the same mass but a different retention time than the enzymatically formed *cis*-diol product. (b) Similar to that observed with the reaction product, the standard of **52** converts into an aromatic molecule (**40**) over time.

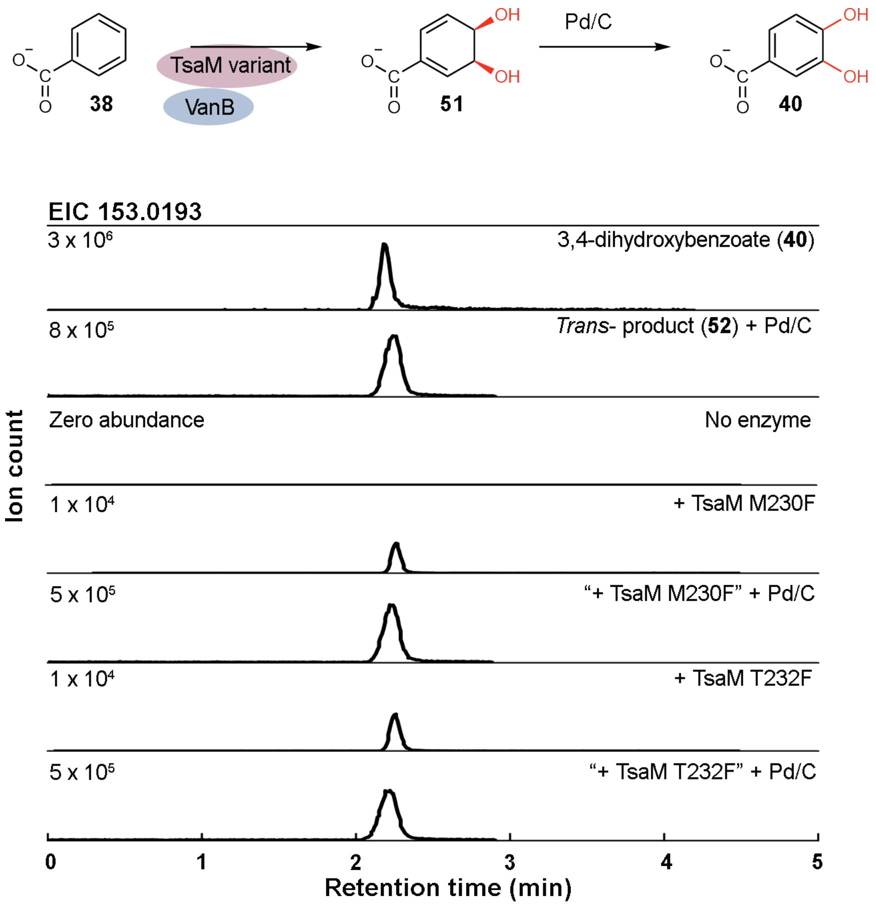

Supplementary Figure 57. The combination of the M230F or T232F TsaM variants, VanB, and **38** results in the formation of a *cis*-diol (**51**), the identify of which was verified via reaction with Pd/C. This reaction resulted in formation of the expected aromatic compound **40** when reacted with either the purchased standard of (3*R*,4*R*)-3,4-dihydroxycyclohexa-1,5-diene-1-carboxylate (**52**) or the product of the TsaM variant reactions.

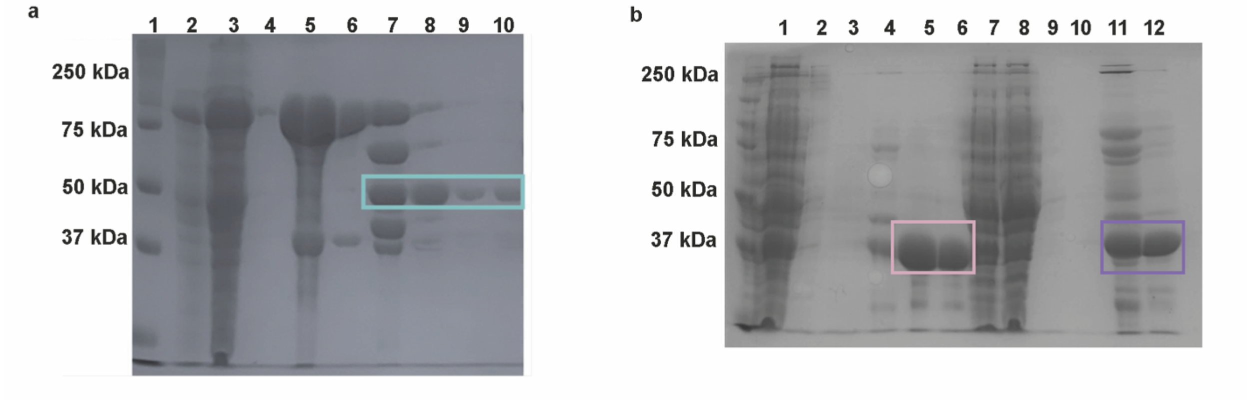

Supplementary Figure 58. Phthalate dioxygenase (PDO) and its annotated reductases, PDR1 and PDR2, were recombinantly expressed and purified using affinity chromatography. (a) The purity of PDO was assessed using SDS-PAGE. PDO has a molecular weight of approximately 49 kDa and its presence was verified by comparison to a protein standard. In this gel, lanes correspond to the supernatant fraction following cell lysis (2-3), the fraction that bound to the MBP column (4-6), the fraction prior to TEV cleavage (7), and the tag cleaved PDO (8-10). The cyan box indicates where PDO runs on the gel. This protein was purified and SDS page assessed approximately four times throughout the duration of this work with similar results. (b) The purity of PDR1 (pink box) and PDR2 (purple box) was assessed using SDS-PAGE. Both PDR1 and PDR2 have a molecular weight of approximately 35 kDa. This protein was purified and SDS-PAGE assessed three times throughout the duration of this work with similar results

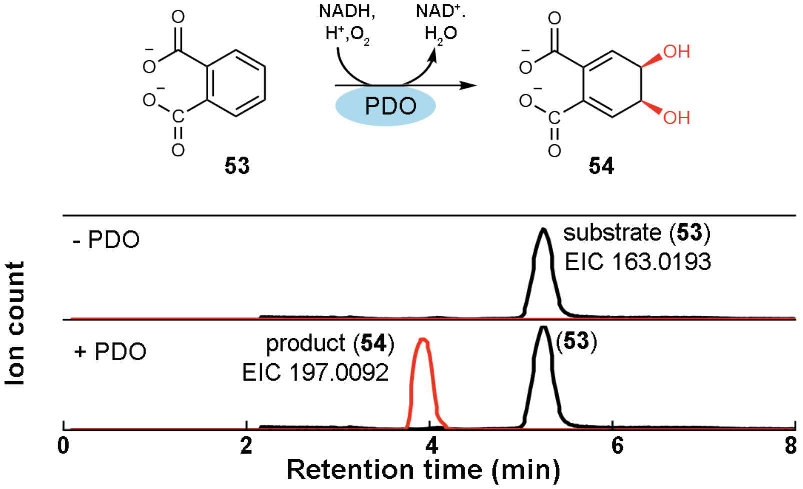

Supplementary Figure 59. The isolated PDO is active on its native phthalate (**53**) substrate. When provided to PDO, as expected, a dioxygenated ((4*R*,5*S*)-4,5-dihydrocyclohexa-2,6-diene-1,2-dicarboxylic acid, **54**) product is formed (red trace, m/z = 197.0092).

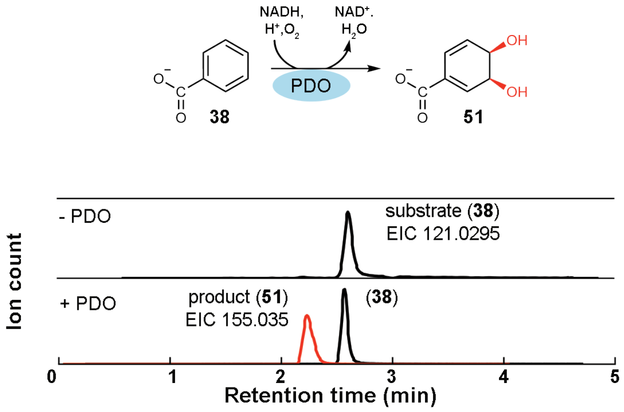

Supplementary Figure 60. The isolated PDO is also active on **38.** The extracted ion chromatogram of the generated dioxygenated product (**51**) is shown in red (m/z = 155.035).

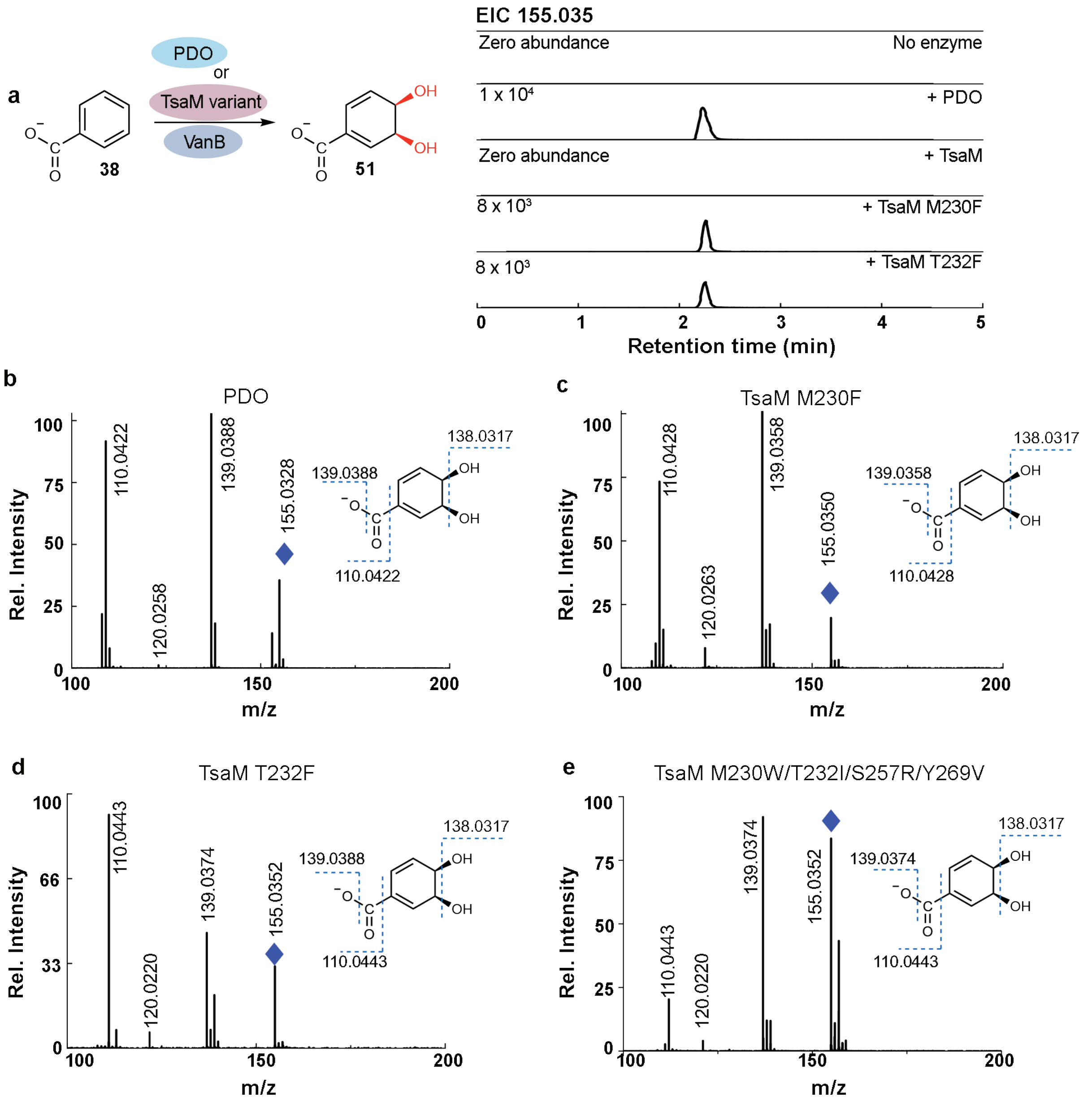

Supplementary Figure 61. The *cis*-diol (**51**) that is produced by the TsaM variants is identical to that produced by PDO when provided with **38** as a substrate. (a) The dioxygenated product (**51**) generated using PDO shares the same retention time with the product of the TsaM M230F and T232F variant reactions. (b) The MS/MS spectrum of the dioxygenated product generated with PDO. (c) The MS/MS fragmentation pattern of the reaction product with TsaM M230F matches that shown in panel b. (d) The MS/MS fragmentation pattern of the reaction product with TsaM T232F matches that shown in panel b. (e) Similarly, the MS/MS fragmentation pattern of the reaction product with TsaM M230W/T232I/S257R/Y269V matches that shown in panel b.

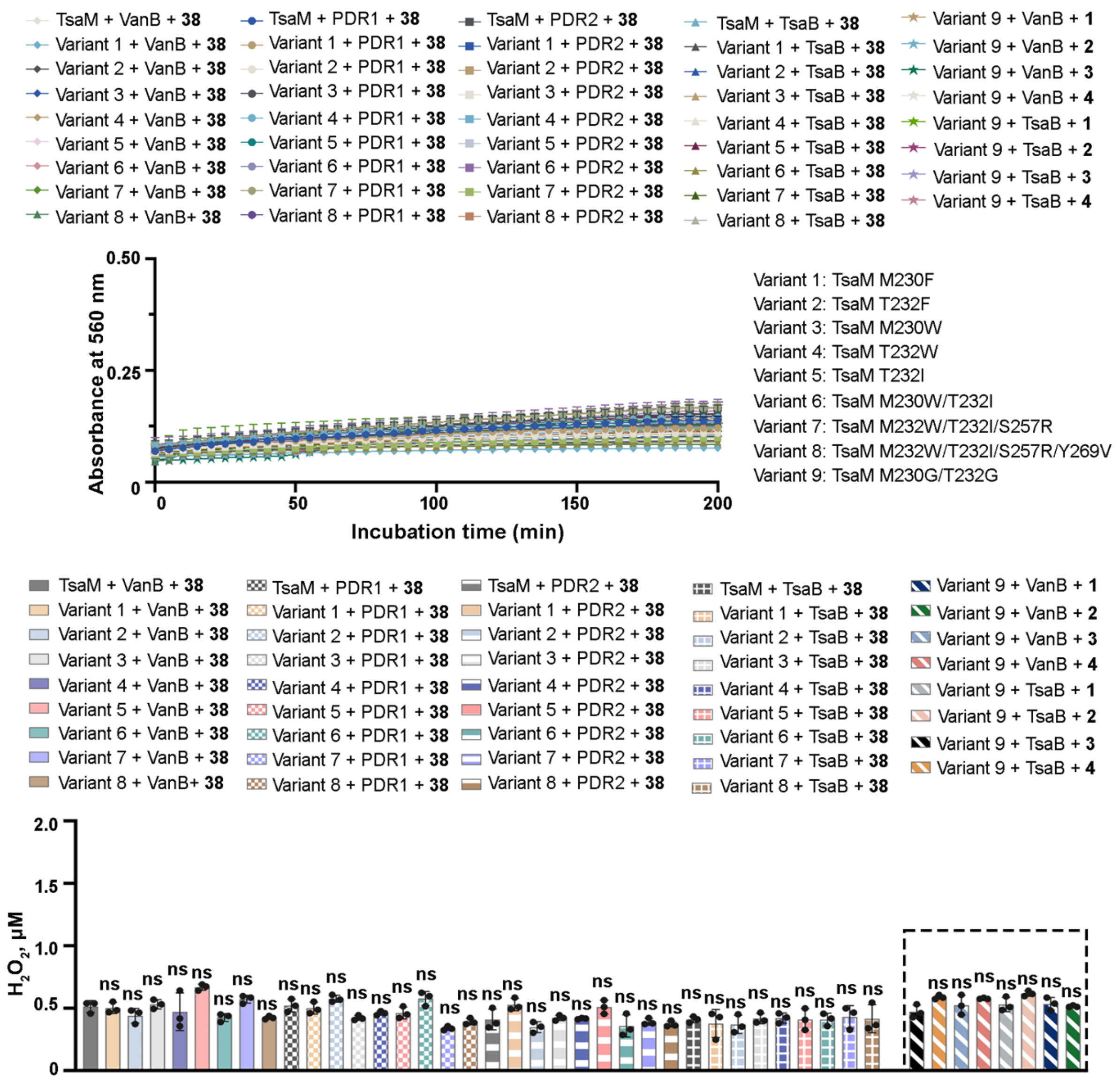

Supplementary Figure 62. An uncoupling assay reveals that the TsaM variant reactions that lead to formation of dioxygenated or sequentially monooxygenated products do not cause uncoupling. For these assays, the absorbance at 560 nm was measured when either wild-type TsaM or a TsaM variant was incubated with a reductase system (VanB, PDR1, PDR2, and TsaB) and benzoate (**12**), *p*-toluenesulfonate (**1**), 4-methylbenzoate (**2**), 4-hydroxymethyl benzenesulfonate (**3**), or 4-(hydroxymethyl)benzenesulfonate (**4**) over a 3 h incubation. The amount of uncoupling was determined by comparison to a reaction that contained TsaM-VanB and either *p*-toluenesulfonate or *p*-methylbenzoate and the absorbance signal from the top panel. The conversion of the absorbance signal at 560 nm to H_2O_2 concentration was calculated using previously described methods and a previously calculated standard curve[4]. The data in the box corresponds to the M230G/T232G TsaM variant that catalyzes sequential monooxygenation reactions rather than dioxygenation chemistry. In this figure, all the data were measured in n = 3 independent experiments and are presented as the mean value ± SD of these measurements. In this figure ns indicates no significant difference from an ordinary one-way ANOVA Tukey analysis. Source data are provided as a Source Data file.

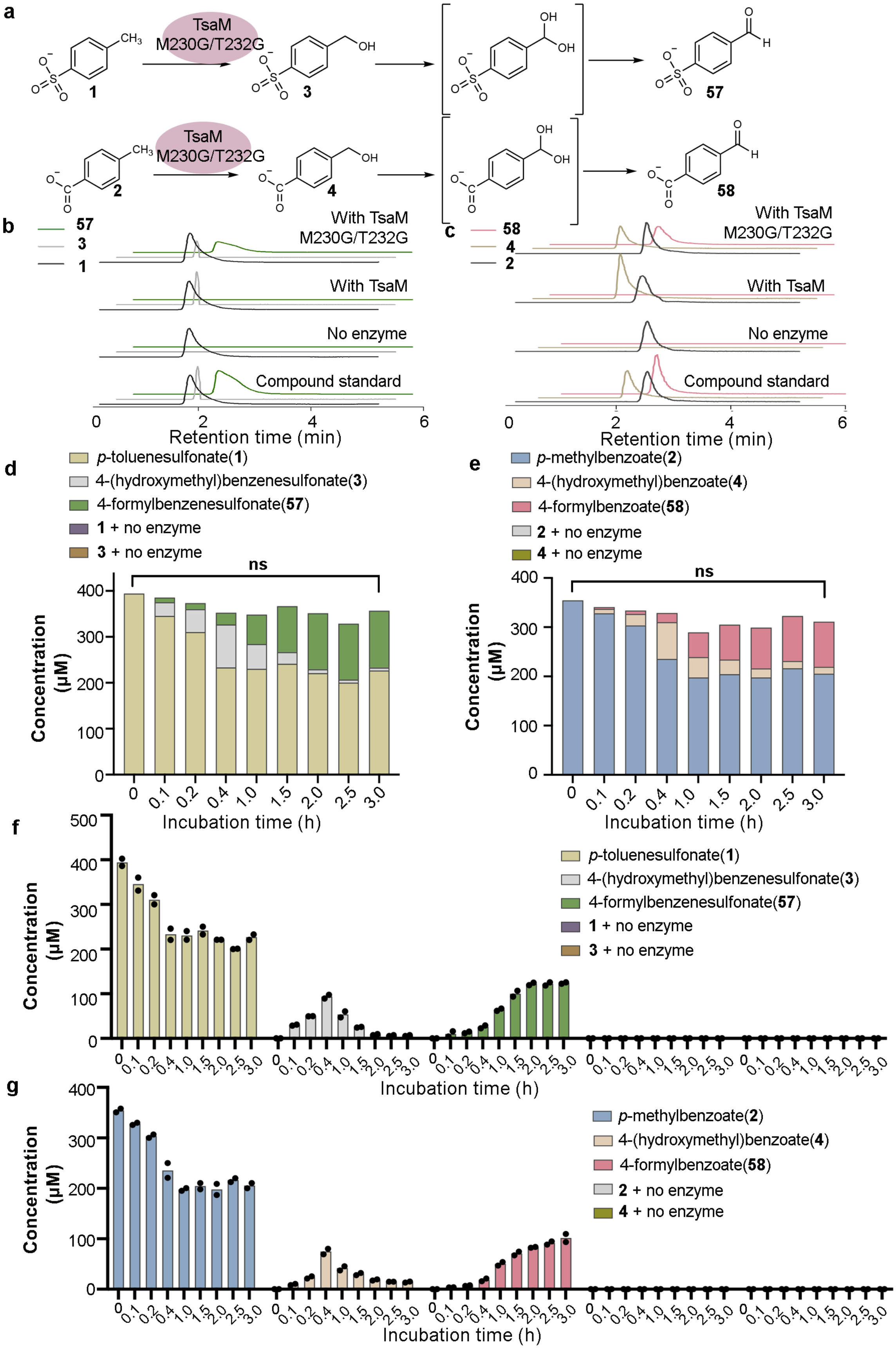
a
TsaM M230G/T232G
1
3
57
2
4
58
b
57
3
1
With TsaM M230G/T232G
With TsaM
No enzyme
Compound standard
Retention time (min)
c
58
4
2
d
p-toluenesulfonate(1)
4-(hydroxymethyl)benzenesulfonate(3)
4-formylbenzenesulfonate(57)
1 + no enzyme
3 + no enzyme
ns
Concentration (μM)
Incubation time (h)
e
p-methylbenzoate(2)
4-(hydroxymethyl)benzoate(4)
4-formylbenzoate(58)
2 + no enzyme
4 + no enzyme
f
g

Supplementary Figure 63. TsaM M230G/T232G catalyzes a sequential monooxygenation reaction on the native substrates of TsaM. (a) The reaction scheme catalyzed by TsaM M230G/T232G on native substrates **1** and **2** to form products **57** and **58**. (b) LC-MS trace of compound standard (**1**, **3**, and **57**), no enzyme control reaction, TsaM wild-type, and the TsaM M230G/T232G catalyzed reaction on native substrate **1.** This data shows that this TsaM variant generates an aldehyde product (**57**). (c) LC-MS traces show that the TsaM variant M230G/T232G also generates an aldehyde product (**58**) when provided with **2** as a substrate. (d) The ratio of mono- and dioxygenated products (**3** and **57**) over time is consistent with sequential oxygenation of **1**. (e) The ratio of mono- and dioxygenated products (**4** and **58**) over time is consistent with sequential oxygenation of **2**. For the mass balance experiments in panels d and e, ns means that the total substrate and product masses were determined to be not significantly different at t = 0 h and t = 3 h using an ANOVA one way Tukey's analysis, where p = 0.2382 for panel d, and p = 0.0979 for panel e. (f) Data from panel d, now showing the total amounts of the substrate (**1**) and products (**3** and **57**) formed over time, and the control reactions that do not contain TsaM. (g) Data from panel e, now showing the total amounts of the substrate (**2**) and products (**4** and **58**) formed over time, and the control reactions that do not contain TsaM. Data for panels d-g were measured using n = 2 independent experiments. Individual data points are represented in panels f-g for the data shown in panels d-e. For the experiments illustrated in panels d-g, the total amount of the products formed saturates and the concentrations were calculated using an internal standard (see Supplementary Figure 73a, b, o, and p). Source data are provided as a Source Data file.

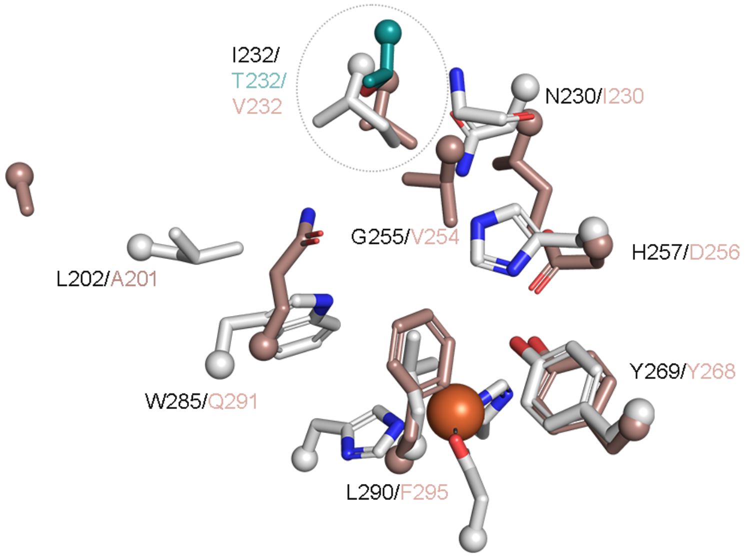

Supplementary Figure 64. A sequence alignment, prior knowledge regarding important active site residues[4], and AlphaFold[6,7] models were used to identify residues that could be mutated to move the substrate closer to the mononuclear iron center in VanA. Selected residues from the AlphaFold models of TsaM (teal) and VanA (mauve) are overlaid with the active site residues of DdmC (PDB: 3GL2[5], gray). Val232, the equivalent of Thr232 (TsaM, dark teal) and Ile232 (DdmC), was mutated into a bulky Phe residue to afford dioxygenation chemistry. This structural overlay was created using Pymol 2.5.2_93 software.

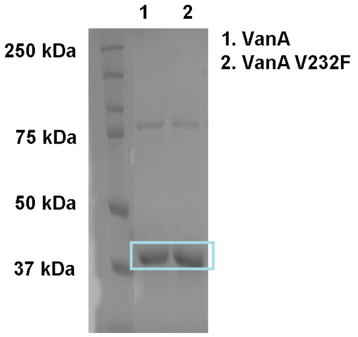

Supplementary Figure 65. Vanillate *O*-demethylase (VanA) and a VanA V232F variant were recombinantly expressed and purified using affinity chromatography using previously described methods[4]. The purity of VanA and VanA V232F was assessed using an SDS-PAGE. VanA has a molecular weight of approximately 38 kDa. These proteins were both purified and SDS-PAGE assessed approximately twice throughout the duration of this work with similar results.

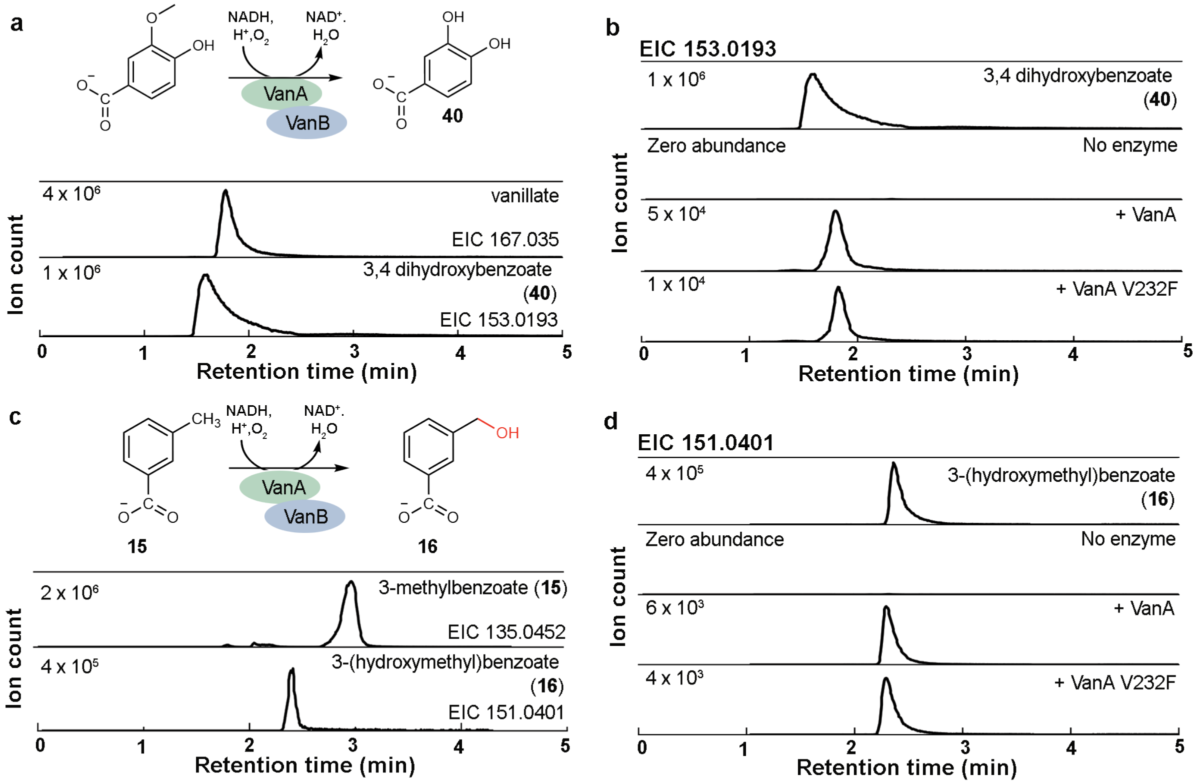

Supplementary Figure 66. The activity of VanA and VanA V232F with the native substrate vanillate and substrate analog 3-methylbenzoate (**15**) was tested with the reductase VanB. (a) The standard of vanillate and **40** can be separated using LC-MS. (b) Recombinantly expressed and purified VanA and VanA V232F each catalyze the expected transformation of vanillate into **40.** (c) An LC-MS method was created to separate a standard of **15** from a standard of **16**. (d) LC-MS revealed that combination of the VanA-VanB system with **15**, results in formation of **16** (m/z=151.0401).

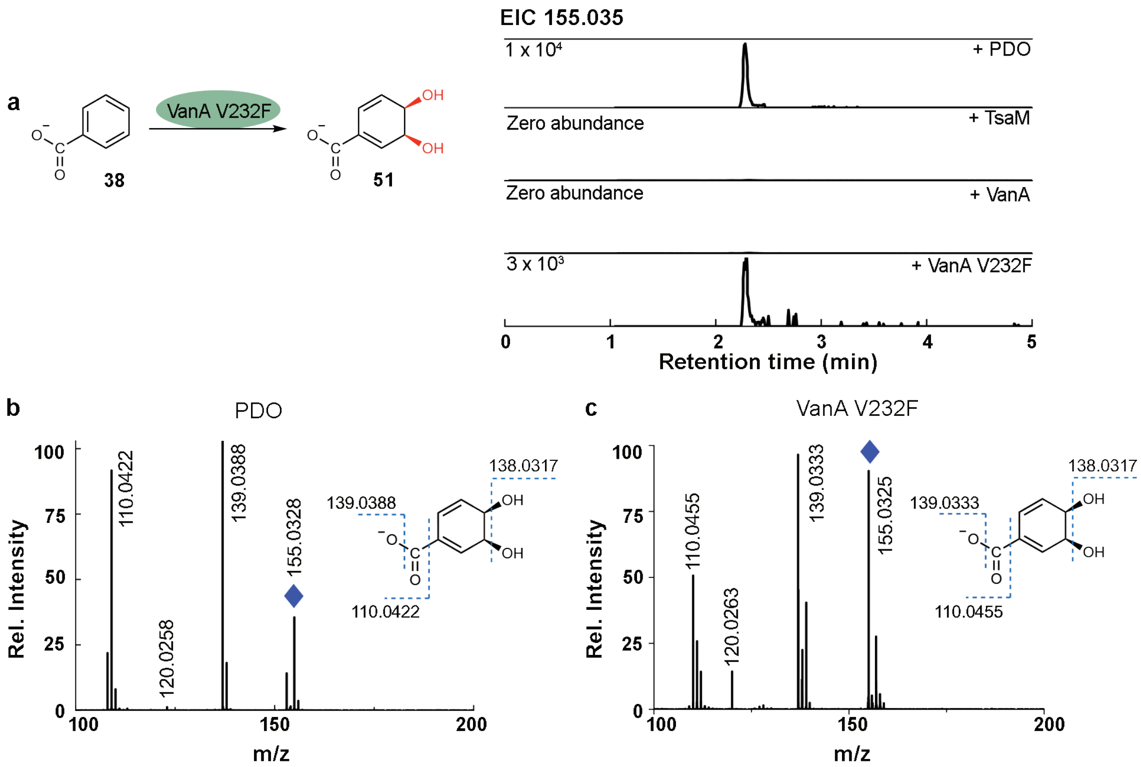

Supplementary Figure 67. The VanA variant produces a *cis*-diol (**51**) when provided with a benzoate (**38**) substrate. (a) Incubation of VanA V232F with **38** results in formation of **51,** which can be observed using LC-MS. The peak generated from the VanA V232F reaction shares the same retention time as the peak generated from the PDO reaction. (b) The MS/MS fragmentation pattern of the product from the PDO reaction. (c) The MS/MS fragmentation pattern of the product from the VanA V232F variant reaction matches that shown in panel b.

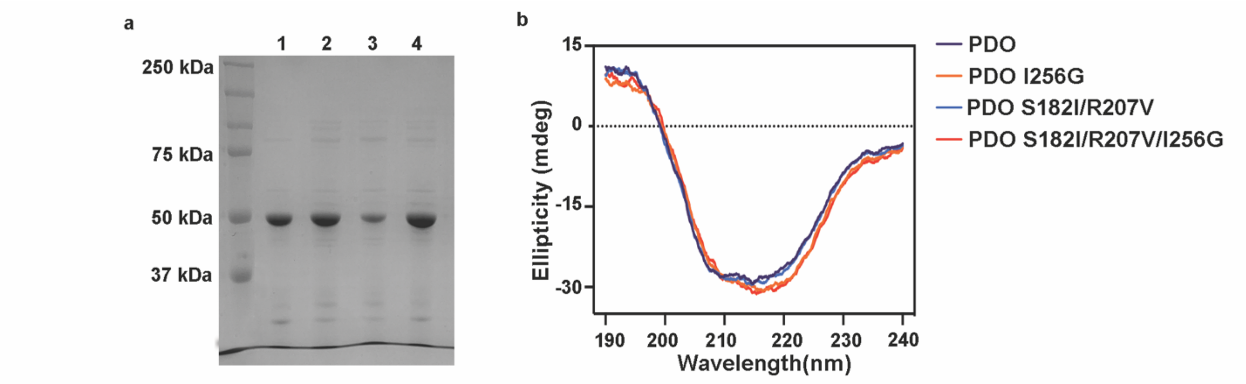

Supplementary Figure 68. PDO variants were recombinantly expressed and purified using affinity chromatography. (a) Each PDO variant has a molecular weight of approximately 49 kDa. The purity and presence of these variants was verified by comparison to a protein standard on SDS-PAGE. Samples correspond to wild-type PDO (1), I256G PDO (2), S182I/R207V PDO (3), and S182I/R207V/I256G (4). (b) Circular dichroism (CD) data of wild-type PDO and variant overlay well, suggesting they are similarly folded. Source data are provided as a Source Data file. These variant proteins were purified and SDS-PAGE assessed approximately twice throughout the duration of this work with similar results.

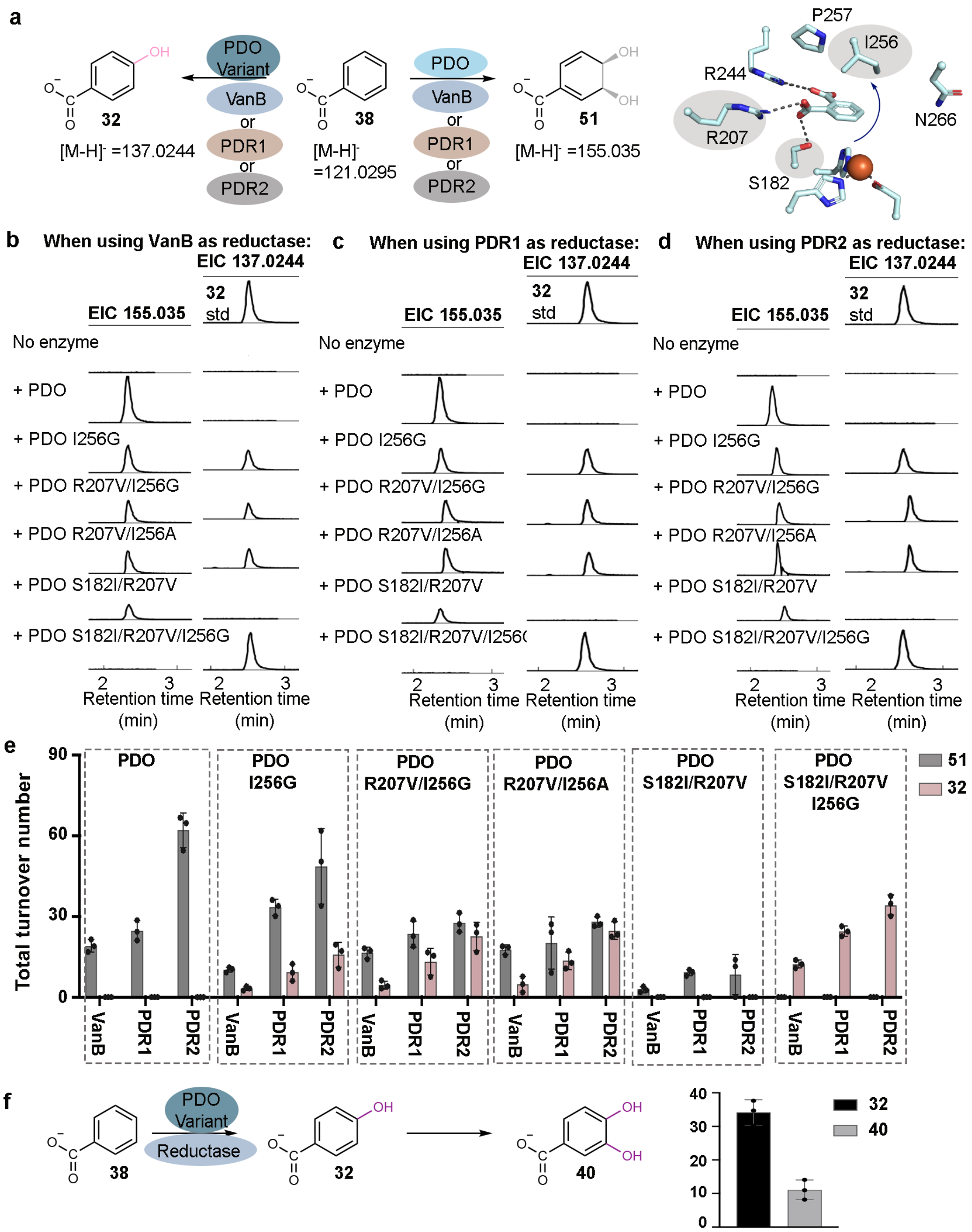

Supplementary Figure 69. The rationally designed PDO variants perform a monooxygenation reaction. (a) The PDO variants were tested for their ability to generate a monooxygenated product (**32**) as well as a dearomatized *cis*-diol (**51**) when **38** is provided as a substrate. (b) LC-MS revealed that combination of the wild-type PDO or S182I/R207V with VanB and **38**, results in formation of only **51** (m/z=155.035). In contrast, combination of the triple variant PDO enzyme with VanB results in formation of only **32** (m/z=137.0244). Finally, combination of I256G PDO with VanB and **38**, results in formation a combination of **32** and **51**. (c-d) A similar trend observed in panel b is also observed with PDR1 and PDR2 in place of VanB. (e)The TTNs are plotted for substrates **51** and **32**. In this panel data was measured using n=3 independent experiments and are presented as mean values ± SD. (f) Combination of the PDO triple variant with **38** and PDR2 leads to formation of monooxygenated (**32**) and dioxygenated compounds (**40**). In this panel data was measured using n=3 independent experiments and are presented as mean values ± SD**.** The amount of **51** and **32** was calculated by using a constructed standard curve of **38** and **32** (see Supplementary Figures 73j and 74e*)*. Source data are provided as a Source Data file.

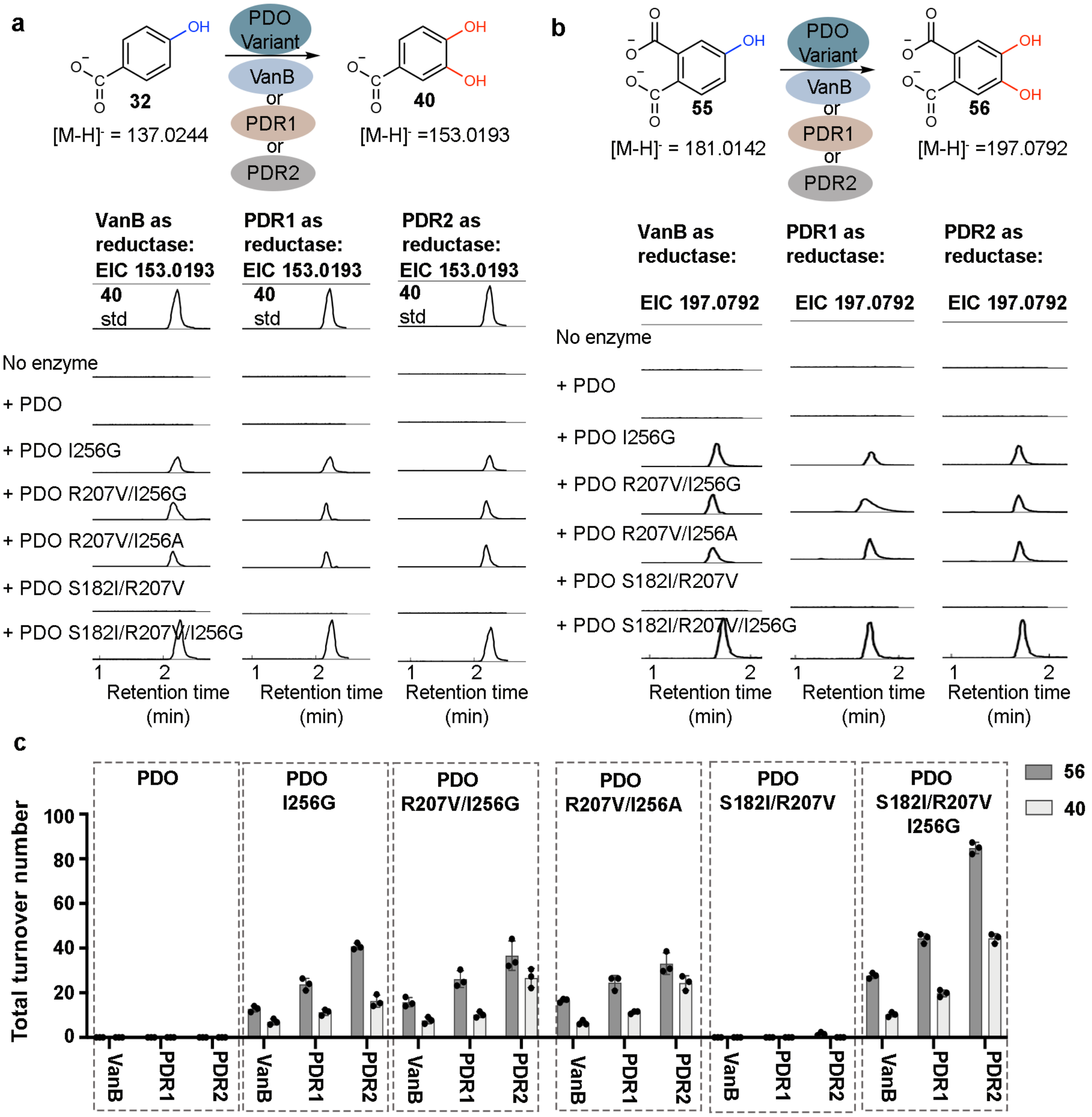

Supplementary Figure 70. The rationally designed PDO variants perform a monooxygenation reaction. (a) All PDO variants, except for S182I/R207V, generate a dioxygenated product (**40**) when **32** is provided as a substrate. (b) Likewise, all PDO variants, except for S182I/R207V, generate a dioxygenated product (**56**) when **55** is provided as a substrate. (c)The TTNs are plotted for production of **56** and **40** by the different combinations of PDO variant and reductases. In this panel data was measured using n=3 independent experiments and are presented as mean values ± SD**.** The amount of **56** and **40** was calculated by using a constructed standard curve of **40** and **55** (see Supplementary Figures 73g and 74e). Source data are provided as a Source Data file.

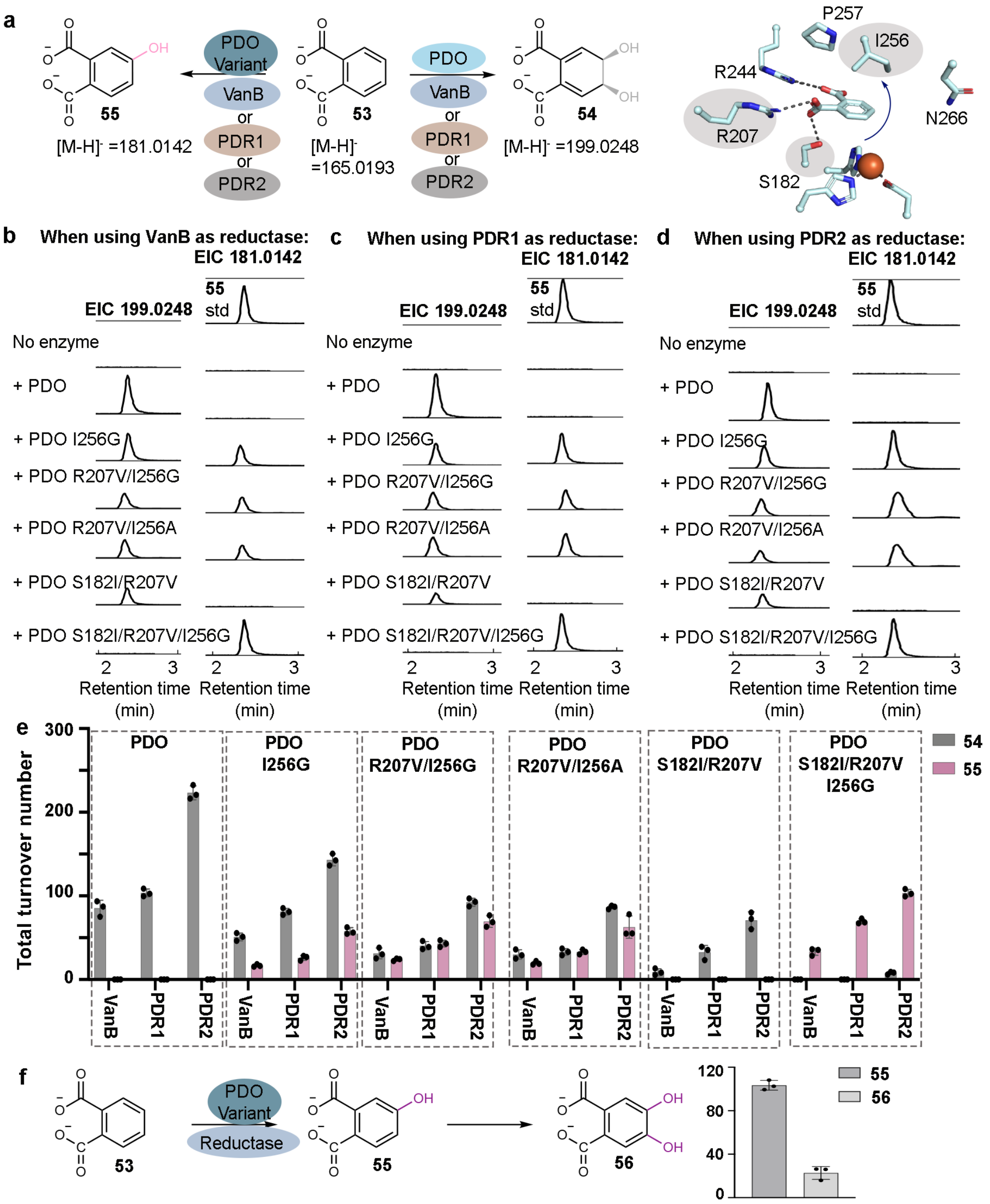

Supplementary Figure 71. The rationally designed variants of PDO perform a monooxygenation reaction on the reported native substrate. (a) The active site of PDO was used to motivate rational mutagenesis experiments (PDB: 7V25[15]). (b) LC-MS revealed that combination of the wild-type PDO or S182I/R207V PDO with VanB and **53** results in formation of only **54** (m/z=199.0248). In contrast, combination of the triple variant PDO enzyme with VanB results in formation of only **55** (m/z=181.0142). Finally, combination of I256G PDO with VanB and **53** results in formation a combination of **54** and **55**. (c-d) A similar trend observed in panel b is also observed with PDR1 and PDR2 in place of VanB. (e) The TTNs are plotted for substrates **54** and **55**. (f) Combination of the PDO triple variant with **53** and PDR2 leads to formation of monooxygenated (**55**) and sequentially monooxygenated compounds (**56**). In all cases, the data is presented as the mean value of these measurements**.** In panels e-f were measured using n=3 independent experiments and are presented as mean values ± SD. The amount of **54** and **55** was calculated by using a constructed standard curve (see Supplementary Figures 73n and 74f). To note, due to the lack of a commercially available product standard of either dioxygenated product, the individual amount of 3,4-dihydroxyphthalate relative to the dearomatized *cis*-diol (**51**) is unable to be quantified. The level of **54**

produced is reflective of any sequential monooxygenation reaction or dearomatized dioxygenation performed. Source data are provided as a Source Data file.

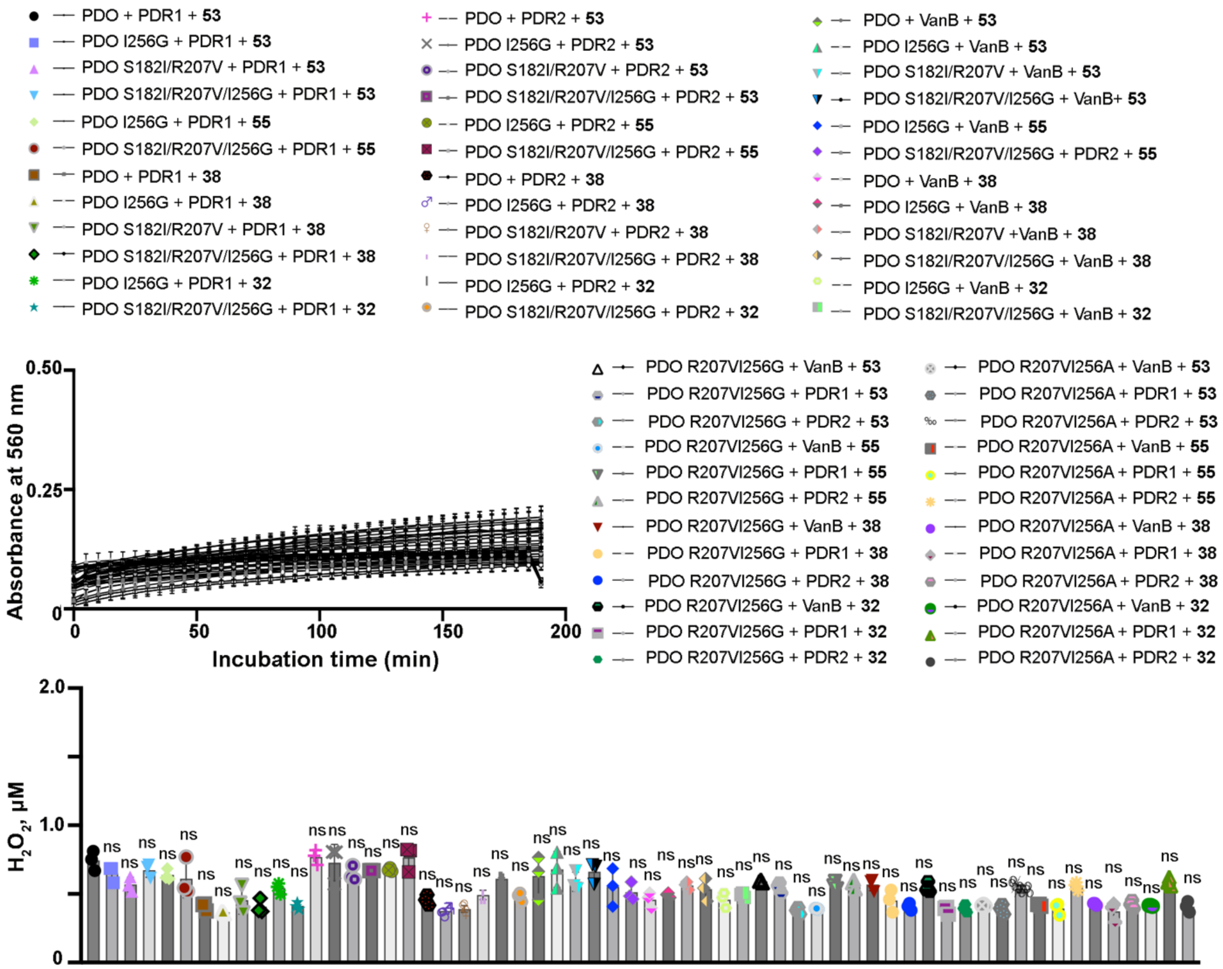

Supplementary Figure 72. An uncoupling assay reveals that the PDO variant reactions that lead to formation of dioxygenated or sequentially monooxygenated products do not cause uncoupling. For these assays, the absorbance at 560 nm was measured when either wild-type TsaM or a TsaM variant was incubated with a reductase system (VanB, PDR1, or PDR2) and phthalate (**53**), benzoate (**38**), 4-hydroxybenzoate (**32**) over a 3 h incubation. The amount of uncoupling was determined by comparison to a reaction that contained TsaM-VanB and either *p*-toluenesulfonate or *p*-methylbenzoate and the absorbance signal from the top panel. The conversion of the absorbance signal at 560 nm to H_2O_2 concentration was calculated using previously described methods and a previously calculated standard curve[4]. In this figure, all the data were measured in n = 3 independent experiments and are presented as the mean value ± SD of these measurements. In this figure, ns indicates no significant difference from an ordinary one-way ANOVA Tukey analysis. Source data are provided as a Source Data file.

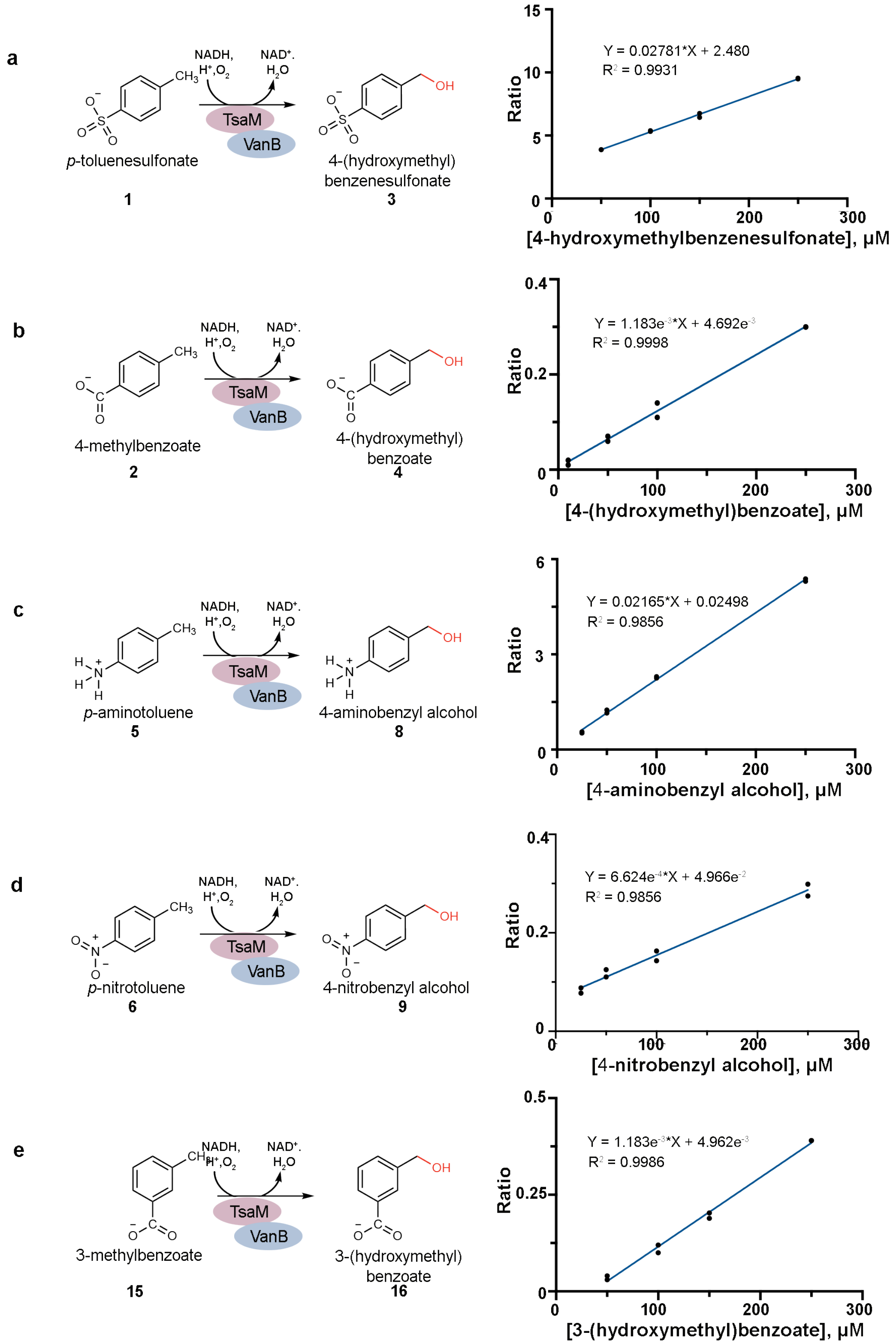
a
NADH, H+,O2
NAD+. H2O
TsaM
VanB
p-toluenesulfonate
1
4-(hydroxymethyl) benzenesulfonate
3
Y = 0.02781*X + 2.480
R2 = 0.9931
Ratio
[4-hydroxymethylbenzenesulfonate], µM
b
4-methylbenzoate
2
4-(hydroxymethyl) benzoate
4
Y = 1.183e-3*X + 4.692e-3
R2 = 0.9998
[4-(hydroxymethyl)benzoate], µM
c
p-aminotoluene
5
4-aminobenzyl alcohol
8
Y = 0.02165*X + 0.02498
R2 = 0.9856
[4-aminobenzyl alcohol], µM
d
p-nitrotoluene
6
4-nitrobenzyl alcohol
9
Y = 6.624e-4*X + 4.966e-2
R2 = 0.9856
[4-nitrobenzyl alcohol], µM
e
3-methylbenzoate
15
3-(hydroxymethyl) benzoate
16
Y = 1.183e-3*X + 4.962e-3
R2 = 0.9986
[3-(hydroxymethyl)benzoate], µM

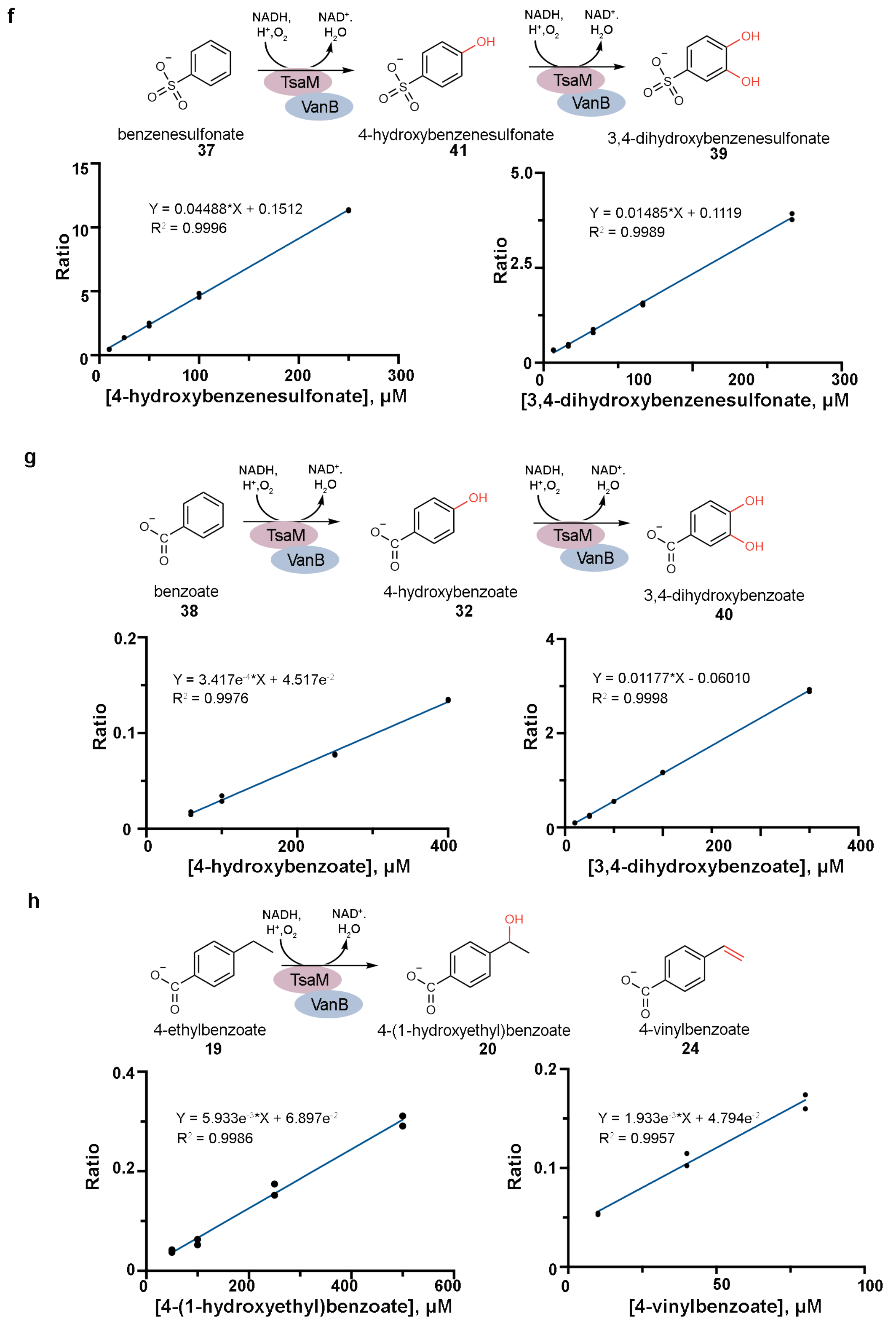
f
NADH, H+,O2
NAD+, H2O
TsaM
VanB
OH
benzenesulfonate
37
4-hydroxybenzenesulfonate
41
3,4-dihydroxybenzenesulfonate
39
Y = 0.04488*X + 0.1512
R2 = 0.9996
Ratio
[4-hydroxybenzenesulfonate], μM
Y = 0.01485*X + 0.1119
R2 = 0.9989
Ratio
[3,4-dihydroxybenzenesulfonate, μM
g
benzoate
38
4-hydroxybenzoate
32
3,4-dihydroxybenzoate
40
Y = 3.417e-4*X + 4.517e-2
R2 = 0.9976
Ratio
[4-hydroxybenzoate], μM
Y = 0.01177*X - 0.06010
R2 = 0.9998
Ratio
[3,4-dihydroxybenzoate], μM
h
4-ethylbenzoate
19
4-(1-hydroxyethyl)benzoate
20
4-vinylbenzoate
24
Y = 5.933e-3*X + 6.897e-2
R2 = 0.9986
Ratio
[4-(1-hydroxyethyl)benzoate], μM
Y = 1.933e-3*X + 4.794e-2
R2 = 0.9957
Ratio
[4-vinylbenzoate], μM

i

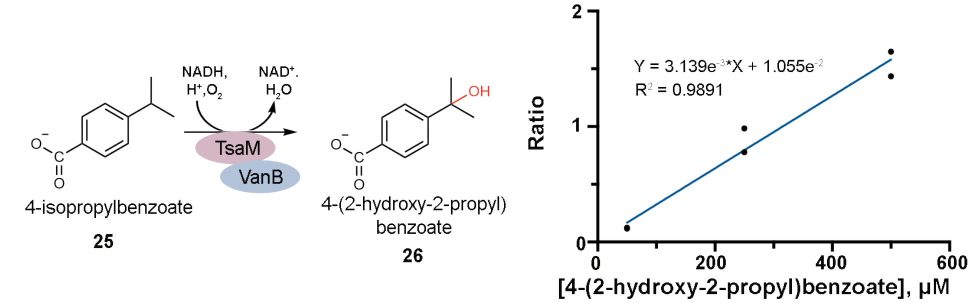

NADH, H+,O2
NAD+. H2O
TsaM
VanB
4-isopropylbenzoate
25
4-(2-hydroxy-2-propyl) benzoate
26
Y = 3.139e-3*X + 1.055e-2
R2 = 0.9891
Ratio
[4-(2-hydroxy-2-propyl)benzoate], µM

j

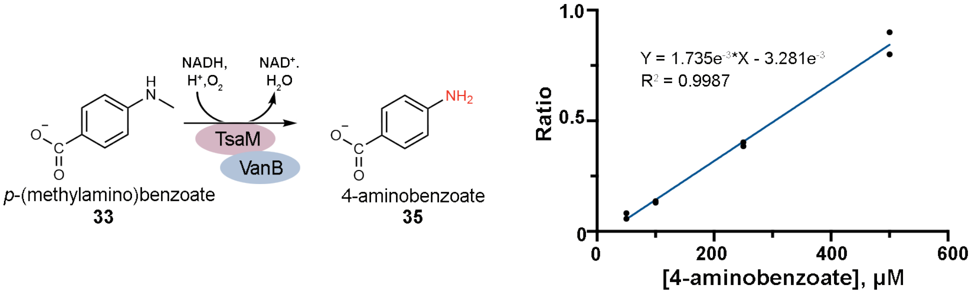

NADH, H+,O2
NAD+. H2O
TsaM
VanB
p-(methylamino)benzoate
33
4-aminobenzoate
35
Y = 1.735e-3*X - 3.281e-3
R2 = 0.9987
Ratio
[4-aminobenzoate], µM

k

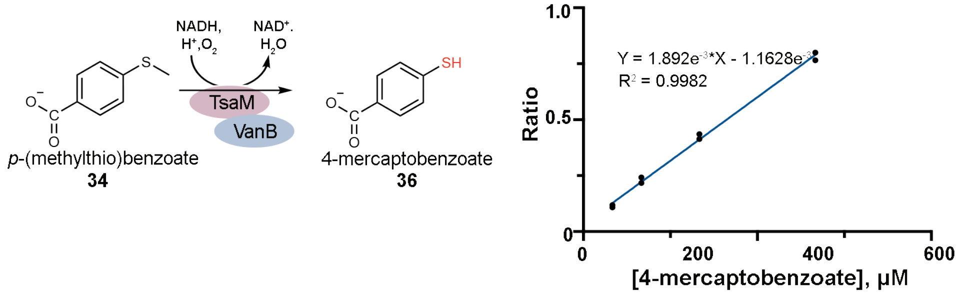

NADH, H+,O2
NAD+. H2O
TsaM
VanB
p-(methylthio)benzoate
34
4-mercaptobenzoate
36
Y = 1.892e-3*X - 1.1628e-3
R2 = 0.9982
Ratio
[4-mercaptobenzoate], µM

l

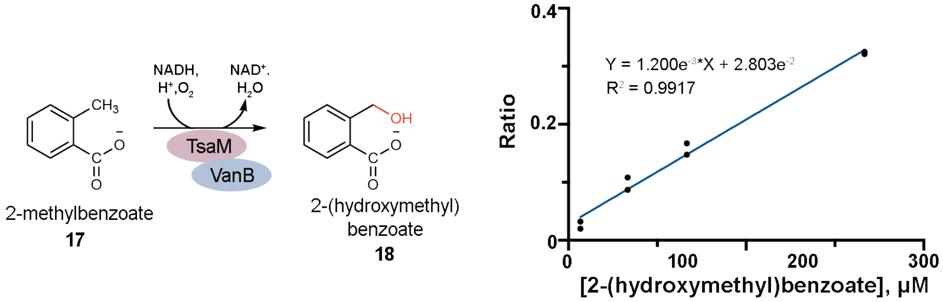

NADH, H+,O2
NAD+. H2O
TsaM
VanB
2-methylbenzoate
17
2-(hydroxymethyl) benzoate
18
Y = 1.200e-3*X + 2.803e-2
R2 = 0.9917
Ratio
[2-(hydroxymethyl)benzoate], µM

m

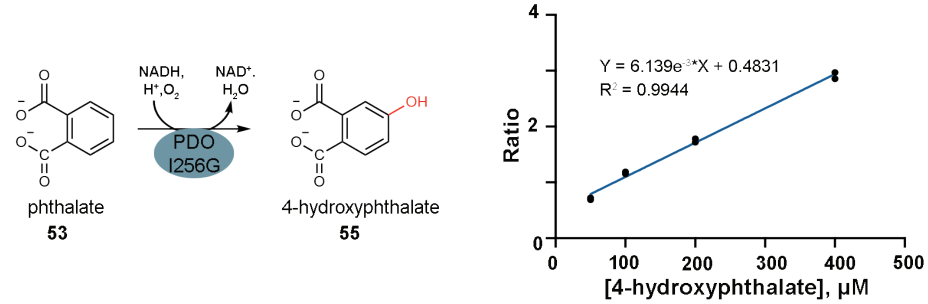

NADH, H+,O2
NAD+. H2O
PDO I256G
phthalate
53
4-hydroxyphthalate
55
Y = 6.139e-3*X + 0.4831
R2 = 0.9944
Ratio
[4-hydroxyphthalate], µM

n

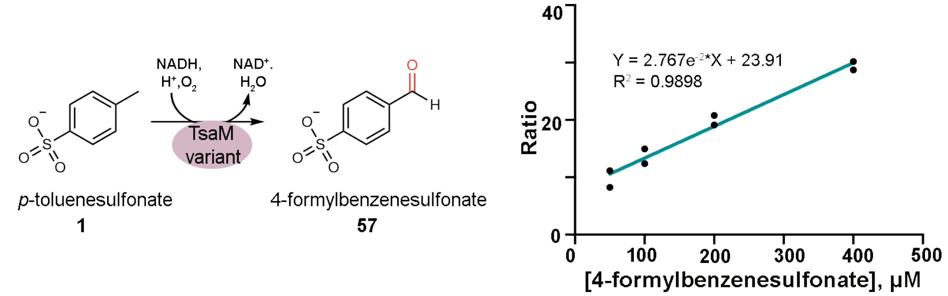

o

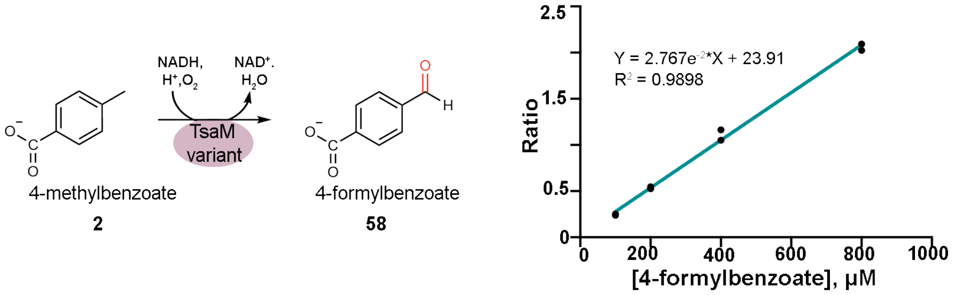

Supplementary Figure 73. The standard curves used to measure product formation in each of the LC-MS based enzymatic assays. In all panels, the x-axis corresponds to the product used and the y-axis is the measured ratio of the peak area of the product: peak area of the internal standard. In order, the standard curves are shown for: (a) 4-(hydroxymethyl)benzenesulfonate (**3**), (b) 4-(hydroxymethyl)benzoate (**4**), (c) 4-aminobenzyl alcohol (**8**), (d) 4-nitrobenzyl alcohol (**9**), (e) 3-(hydroxymethyl)benzoate (**16**), (f) 4-hydroxybenezesulfonate (**41**, left panel) and 3,4-dihydroxybenzenesulfonate (**39**, right panel), (g) 4-hydroxybenzoate (**32**, left panel), 3,4-dihydroxybenzoate (**40**, right panel), (h) 4-(1-hydroxyethyl)benzoate (**20**, left panel) and 4-vinylbenzoate (**24**, right panel), (i) 4-(2-hydroxy-2-propyl)benzoate (**26**), (j) 4-aminobenzoate (**35**), (k) 4-mercaptobenzoate (**36**), (l) 2-(hydroxymethyl)benzoate (**18**), (m) 4-hydroxyphthalate (**55**), (n) 4-formylbenzenesulfonate (**57**), and (o) 4-formylbenzoate (**58**). In this figure, the data were measured using n = 2 independent experiments. Source data are provided as a Source Data file.

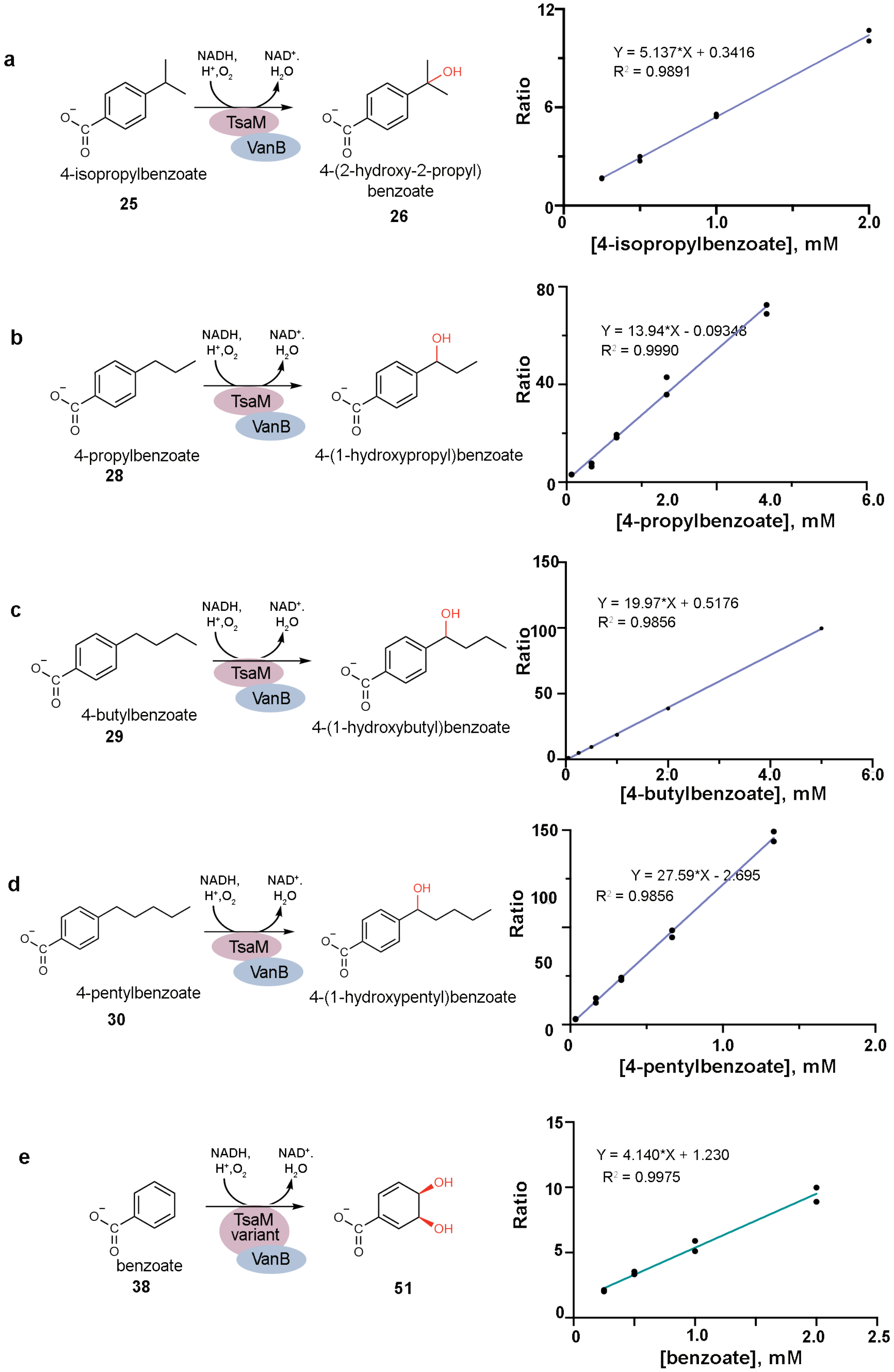
a
NADH, H+,O2
NAD+. H2O
TsaM
VanB
OH
4-isopropylbenzoate
25
4-(2-hydroxy-2-propyl) benzoate
26
Y = 5.137*X + 0.3416
R2 = 0.9891
Ratio
[4-isopropylbenzoate], mM
b
4-propylbenzoate
28
4-(1-hydroxypropyl)benzoate
Y = 13.94*X - 0.09348
R2 = 0.9990
[4-propylbenzoate], mM
c
4-butylbenzoate
29
4-(1-hydroxybutyl)benzoate
Y = 19.97*X + 0.5176
R2 = 0.9856
[4-butylbenzoate], mM
d
4-pentylbenzoate
30
4-(1-hydroxypentyl)benzoate
Y = 27.59*X - 2.695
R2 = 0.9856
[4-pentylbenzoate], mM
e
TsaM variant
benzoate
38
51
Y = 4.140*X + 1.230
R2 = 0.9975
[benzoate], mM

f

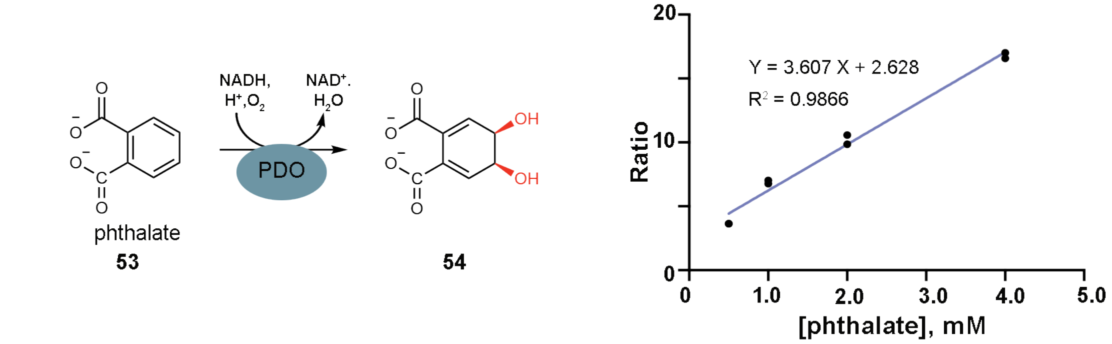

Supplementary Figure 74. The standard curves used to measure substrate consumption in each of the LC-MS based enzymatic assays. In all panels, the x-axis corresponds to the concentration of the substrate used and the y-axis is the measured ratio of the peak area of the substrate: peak area of the internal standard. In order, the standard curves are shown for: (a) 4-isopropylbenzoate (**25**), (b) 4-propylbenzoate (**28**), (c) 4-butylbenzoate (**29**), (d) 4-pentylbenzoate (**30**), (e) benzoate (**38**), and (f) phthalate (**53**). In this figure, all of the data were measured using n = 2 independent experiments. Source data are provided as a Source Data file.

Coordinates of optimized geometries and absolute energies of calculated substrates

<u>4-methylbenzoate</u>

-459.537974394701 hartrees

C	-4.54702074298506	1.99453996573033	0.08001276749091
C	-4.60016564456092	0.60390652496477	0.05956853513919
C	-3.43836278508864	-0.16162100622968	0.12148318894904
C	-2.21719726762972	0.50702629037829	0.20468677129974
C	-3.32531658728304	2.66289677242470	0.15406373602047
C	-2.16011793888439	1.89520103549388	0.21525952780680
C	-3.25378757326865	4.16727385388618	0.17913492889139
H	-4.21536522155561	4.61260623851257	-0.08359653780340
H	-2.50990157344699	4.54377250779388	-0.52667796433775
H	-2.96877147358523	4.54103168832433	1.16806364306059
H	-5.46480883036038	2.57276702379598	0.03129417200898
H	-5.53906885658165	0.07141795712800	-0.01392907217637
C	-3.50565499913586	-1.70901231271176	0.08838547762396
O	-4.64981079595005	-2.18506630432558	-0.08831246075417
O	-2.41182566684713	-2.29819393649958	0.24035825029328
H	-1.19907461758505	2.39778709506482	0.27156565002475
H	-1.32241942525165	-0.09901339373117	0.25658938646259

<u>*p*-toluenesulfonate</u>

-894.834084742024 hartrees

C	-1.77061645822939	1.33127430237941	0.66516409941531
C	-1.65910827805387	-0.05166519165798	0.75763416999719
C	-0.57730305058591	-0.62465510417723	1.41595197074268
C	0.39507871628558	0.20256863110879	1.96915198714086
C	-0.80688067088998	2.17100722827056	1.22074783553857
C	0.27927300280820	1.58318379921222	1.87373363991755
H	-2.61584031587443	1.76845123794312	0.14480149022815
C	-0.93875165312651	3.66890110369273	1.13205627355094
H	1.24147249214737	-0.25639769529420	2.46077178992079
H	1.04602210444417	2.21618463752201	2.30839425786094
H	-2.39784125245339	-0.70272175181316	0.31141765672286
S	-0.46276167342189	-2.41789504298982	1.61078706748035
O	-1.11560365787364	-2.96379891834399	0.42164095890694
O	-1.19142946074600	-2.65946692557656	2.85619193818792
O	0.97136973694915	-2.68585930094372	1.69189572928608
H	-1.66441315635325	3.95830758274591	0.37176534705632
H	0.01451787114236	4.13854415016511	0.87804612179806
H	-1.27495429616857	4.09793725775678	2.08160766624846

4-(hydroxymethyl)benzenesulfonate

-970.055654532235 hartrees

C	-1.82326000490056	1.30329943972252	0.69711969926394
C	-1.70136508828326	-0.08048815673849	0.78064191245674
C	-0.59861049911773	-0.64630415664598	1.41084286261342
C	0.38360852229086	0.18402150201286	1.94575127260962
C	-0.85236352910323	2.14171161450667	1.24374915010447
C	0.25465435041044	1.56173440058657	1.87095580194738
H	-2.68152741532517	1.74065894307578	0.19815123448718
C	-0.96490010924316	3.63604678941653	1.14138960446496
H	1.24284492244211	-0.27500720800595	2.41335804219360
H	1.02173628467952	2.20400024328087	2.28696168893203
H	-2.44677546184076	-0.73548747891590	0.35100964671859
S	-0.45312800110758	-2.43876898028527	1.60735751956319
O	-1.12190387600331	-2.99769211540653	0.43396030165513
O	-1.14958366199183	-2.68034676589410	2.87057669070938
O	0.98660101681775	-2.68012835355499	1.65818368204572
H	-1.89768020831946	3.90202598386028	0.63063939589055
O	0.15344110300455	4.23793528184232	0.46873220846954
H	-0.98164694541651	4.09845349233688	2.13068244819629
H	0.42140860100734	3.62638552480591	-0.22388316232174

4-(hydroxymethyl)benzoate

-534.760675315505 hartrees

C	-4.50465723090645	2.01163678443072	-0.02091104117372
C	-4.56887807978421	0.62504869607253	-0.08347811999411
C	-3.42899513381920	-0.15501447040355	0.10002212068809
C	-2.21570416078879	0.49282290379454	0.33316715051267
C	-3.29231016636762	2.66065416661077	0.22894879000023
C	-2.14606639492278	1.87907836857725	0.39793038495187
C	-3.23048811598161	4.15838024999887	0.27064513866342
H	-4.20269283810212	4.56045003871397	0.57701364367863
O	-2.86243819408442	4.74870135515669	-0.99330900946494
H	-2.48173797506181	4.49980005523665	0.98802438484864
H	-5.40321530276891	2.60493727430389	-0.15905464281274
H	-5.49847068946350	0.10349724946209	-0.26748977194068
C	-3.51932061700685	-1.70317759092892	0.06153313524794
O	-4.66986218138777	-2.15690342979881	-0.12675438260421
O	-2.43671649060393	-2.30701821725223	0.22671131691623
H	-1.19756112867216	2.36804111858811	0.59405230675233
H	-1.34243044932874	-0.12931654490731	0.47789245905008
H	-3.05325485094907	4.08985199234472	-1.66714386331974

p-(methoxy)benzoate

-534.747867443463 hartrees

C	-4.54584607422121	2.31495636389391	0.03397471707990
C	-4.55333022312740	0.91866409370214	0.02969033242600
C	-3.38025606137637	0.18002165153813	0.12236231916696
C	-2.17466251162206	0.87954067004136	0.21124832725926
C	-3.33128611354309	2.98905915080263	0.12783852488217
C	-2.14167948479261	2.26591418926025	0.21044811838438
H	-5.47904013570763	2.85580214267900	-0.03751784311815
H	-5.48045389264984	0.36615614088211	-0.04792017510462
C	-3.40972085280538	-1.36754103361733	0.13048006338734
O	-4.54092766052066	-1.87998974046666	-0.02734417712434
O	-2.29970293926427	-1.92266435070278	0.29485867327409
H	-1.20962189183824	2.81351073038818	0.27884448900713
H	-1.26784206858830	0.29440470702944	0.28572580478902
O	-3.20247189282024	4.36493077342016	0.14965697334340
C	-4.37721192749053	5.13569634552842	0.08060379284843
H	-4.06160014617985	6.17632304283904	0.14619438338072
H	-5.06039747355782	4.91438630381260	0.90721683457378
H	-4.91487864989447	4.97787881896939	-0.86136115845547

p-(methylamino)benzoate

-514.878396803185 hartrees

C	-4.51979237101871	2.29349233475451	-0.06098663342511
C	-4.53841849207353	0.89968545012392	-0.02328036432474
C	-3.37484405355680	0.15443142002369	0.12873113463614
C	-2.17203908897807	0.85275438343681	0.24768609164481
C	-3.30856863599344	2.98189308738260	0.04161489017462
C	-2.13262976408824	2.23768872592956	0.20452208259315
H	-5.44687637838403	2.83905398457804	-0.17927157722648
H	-5.46752690596519	0.35231427828610	-0.11390533052786
C	-3.40796822579548	-1.39087273216640	0.16562555584186
O	-4.53946533529571	-1.90559238950524	0.01481412349497
O	-2.29907008236526	-1.94698468792897	0.34044548199118
H	-1.18472740443380	2.76150252997679	0.28930953886208
H	-1.27179690690358	0.26628592995378	0.37609636632791
N	-3.22340171779215	4.38399952718089	-0.05833616799396
C	-4.39926210511097	5.20058676374551	0.13665240956613
H	-4.09793032921798	6.24840965004730	0.16201584366836
H	-4.95239947802851	4.97091454753813	1.05992763539201
H	-5.09420209091992	5.07748663499712	-0.69665543952744
H	-2.40992063407853	4.74526056164581	0.41311435883235

<u>*p*-(methylthio)benzoate</u>

-857.700875596015 hartrees

C	-4.40394870180580	2.30064276870033	-0.05670991047262
C	-4.48828720116000	0.91159064410921	-0.01274984305529
C	-3.35555436297697	0.11563181547012	0.12433391902167
C	-2.11723424538084	0.75213293473499	0.20595483968494
C	-3.16048478059869	2.92319473855624	0.03993660809617
C	-2.01285542340764	2.13541072169759	0.16883713334730
H	-5.30627171765836	2.88494520035833	-0.16943094381253
H	-5.44250991150892	0.40682274775646	-0.08535161457802
C	-3.47274559639739	-1.42651750275574	0.19989717107637
O	-4.63649937085140	-1.87273498664493	0.09090356369973
O	-2.39465984891874	-2.03938753329076	0.36574703446987
H	-1.04333162338148	2.61351384239497	0.25228806756871
H	-1.24303734211202	0.12421284184629	0.31535490735471
S	-2.92149857583236	4.68528874589663	-0.00225495299612
C	-4.60776965270562	5.32779507709674	0.12586158919047
H	-4.51853908596547	6.40292265813419	0.27720925215250
H	-5.12597963404510	4.89023318716152	0.97738126298499
H	-5.17972292529315	5.14135209877780	-0.78220808373286

<u>benzoate</u>

-420.230758824772 hartrees

C	-4.55080300875837	2.02664329140789	0.07904066589669
C	-4.60040245270845	0.63649003235651	0.05434065017857
C	-3.43430398390231	-0.12619328785298	0.11658627091655
C	-2.21094814112907	0.53807893674755	0.19996739718826
C	-3.32315017862498	2.67962532848096	0.15829134261593
C	-2.15112545615942	1.92873323215847	0.21605230208206
H	-3.27920680643636	3.76273034692103	0.17493282164507
H	-5.46699465308669	2.60539102400929	0.03415889160839
H	-5.53674214524667	0.09946739792672	-0.01724721087452
C	-3.50529067207291	-1.67546019699666	0.09102911918782
O	-4.65118035686828	-2.14865506261335	-0.08100844270069
O	-2.41319277273127	-2.26666485624592	0.24471911563870
H	-1.19219281344060	2.43216616616748	0.27656394148706
H	-1.31910655883460	-0.07242235246700	0.25273313513011

4-hydroxybenzoate

-495.459177540199 hartrees

C	-4.54559209370127	2.02577457411057	0.07750373434773
C	-4.59710139495446	0.63510807657692	0.05333271354933
C	-3.43748345317140	-0.13345210930843	0.11659396805601
C	-2.21583392312108	0.53319049003880	0.20048783333311
C	-3.31397953370789	2.66880285857398	0.15643981765074
C	-2.14292770218154	1.92258914820213	0.21588862846764
O	-3.20826698021050	4.05043324533627	0.17678783631613
H	-5.45856061607784	2.61418531438752	0.03149539489255
H	-5.53754522981787	0.10537494241952	-0.01924474772231
C	-3.51484012683654	-1.67966132275534	0.09112829435225
O	-4.66394010992068	-2.14852504667023	-0.07579774516666
O	-2.42497348333195	-2.27660456348074	0.23980307808216
H	-1.19344007975094	2.44004006437467	0.27688599736004
H	-1.32301135793302	-0.07546267572384	0.25472122487348
H	-4.09770391528302	4.41260700391818	0.14179397160780

benzenesulfonate

-855.526727032854 hartrees

C	-1.77867706296890	1.36099572415127	0.65999434584753
C	-1.66160503370114	-0.02180871149243	0.74826894396959
C	-0.57585298428832	-0.59146079837703	1.40741844670334
C	0.39876912812131	0.23122125004736	1.96341332914064
C	-0.81047164935284	2.18508432153274	1.22713403232644
C	0.28070963635274	1.61399483865558	1.87772909840435
H	-2.62460138349153	1.79903680350073	0.14327311779821
H	-0.90484048037942	3.26207231497421	1.15680323449786
H	1.24320919604898	-0.23249450071228	2.45370557914088
H	1.04146802566320	2.24824046037740	2.31746630697745
H	-2.39814335295190	-0.67758101169256	0.30515569851699
S	-0.46547763065520	-2.38626354283162	1.60929992163429
O	-1.12226275681760	-2.93591137135375	0.42421122306616
O	-1.19287182403343	-2.61761802092664	2.85728573439707
O	0.96772817245405	-2.65842775585299	1.68917098757919

4-hydroxybenzenesulfonate

-930.754832695926 hartrees

C	-1.77906998814352	1.35956829745053	0.66558462363585
C	-1.66523060459111	-0.02337763828043	0.76031381221942
C	-0.58222066038393	-0.59825029007332	1.41464604394230
C	0.38904626951699	0.22786847920119	1.97054100226733
C	-0.80083045482448	2.17516291292651	1.22533991140449
C	0.28551754958616	1.61073488283166	1.88375424456816
H	-2.62295143382051	1.80579063730479	0.14788198396564
O	-0.86143432432903	3.55260745398457	1.13907338742185
H	1.23249606071786	-0.23254681120641	2.46521083957060
H	1.03599321280900	2.26004832507148	2.31562846009526
H	-2.40781799027776	-0.67225342959028	0.31724457424695
S	-0.46864590049745	-2.39155535404286	1.60177425439732
O	-1.12415613478340	-2.92883976666953	0.40958768705817
O	-1.19441866664634	-2.64506417907226	2.84638188148588
O	0.96606796374717	-2.65881232004262	1.67787723749692
H	-		

Uncropped Gels from Supplementary Figures

Supplementary Fig. 2a

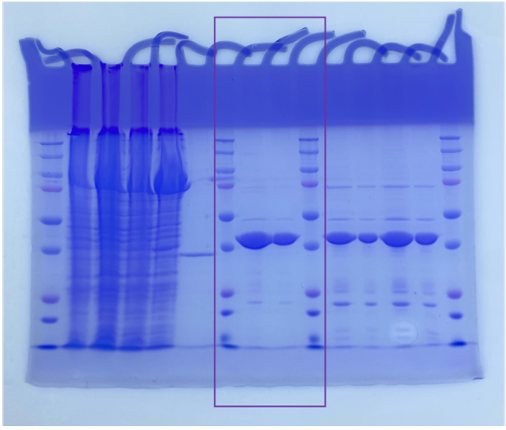

Supplementary Fig. 3a

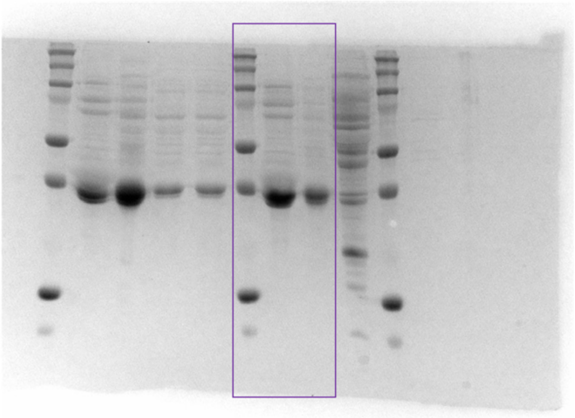

Supplementary Fig. 51

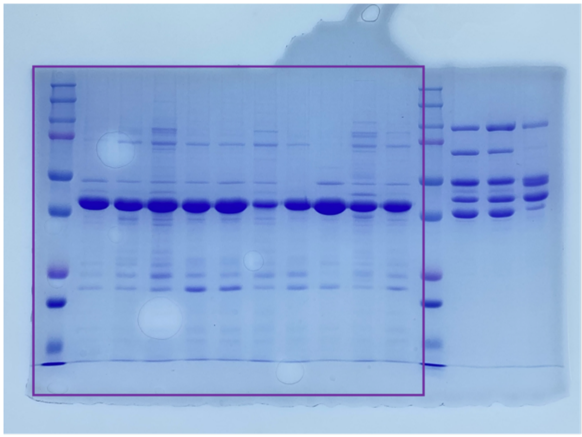

Supplementary Fig. 58a-b

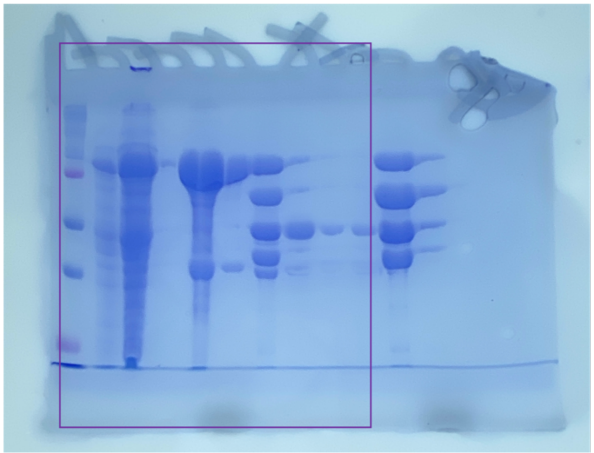

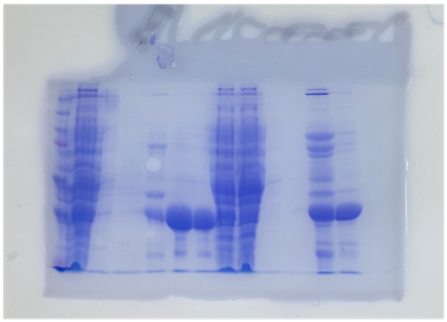

Supplementary Fig. 65

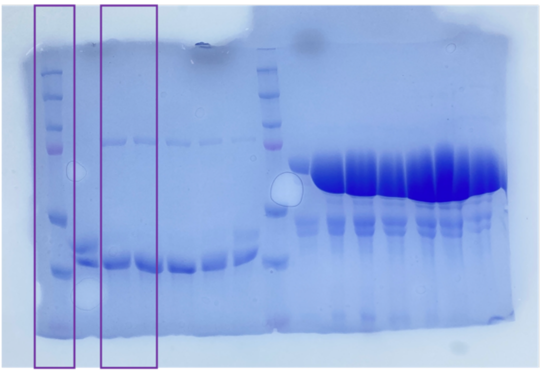

Supplementary Fig. 68a

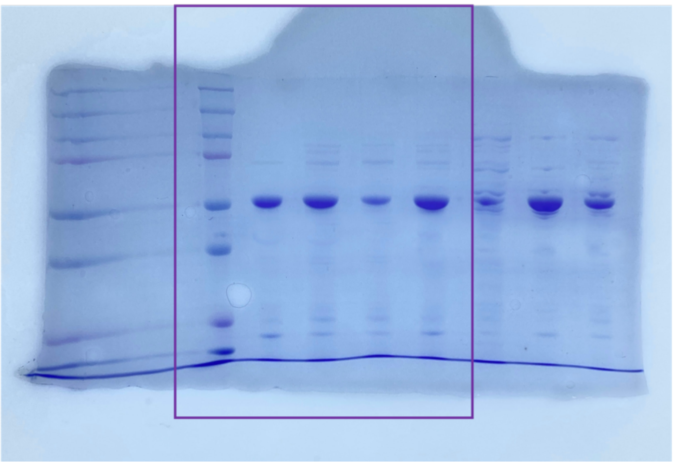

Supplementary References

1 Rogers, M. S. & Lipscomb, J. D. Salicylate 5-Hydroxylase: Intermediates in Aromatic Hydroxylation by a Rieske Monooxygenase. *Biochemistry*, doi:10.1021/acs.biochem.9b00292 (2019).

2 Rivard, B. S. *et al.* Rate-Determining Attack on Substrate Precedes Rieske Cluster Oxidation during Cis-Dihydroxylation by Benzoate Dioxygenase. *Biochemistry* **54**, 4652-4664, doi:10.1021/acs.biochem.5b00573 (2015).

3 Rogers, M. S., Gordon, A. M., Rappe, T. M., Goodpaster, J. D. & Lipscomb, J. D. Contrasting Mechanisms of Aromatic and Aryl-Methyl Substituent Hydroxylation by the Rieske Monooxygenase Salicylate 5-Hydroxylase. *Biochemistry* **62**, 507-523, doi:10.1021/acs.biochem.2c00610 (2023).

4 Tian, J., Garcia, A. A., Donnan, P. H. & Bridwell-Rabb, J. Leveraging a Structural Blueprint to Rationally Engineer the Rieske Oxygenase TsaM. *Biochemistry* **62**, 1807-1822, doi:10.1021/acs.biochem.3c00150 (2023).

5 Dumitru, R., Jiang, W. Z., Weeks, D. P. & Wilson, M. A. Crystal structure of dicamba monooxygenase: a Rieske nonheme oxygenase that catalyzes oxidative demethylation. *J Mol Biol* **392**, 498-510, doi:10.1016/j.jmb.2009.07.021 (2009).

6 Mirdita, M. *et al.* ColabFold: making protein folding accessible to all. *Nat Methods* **19**, 679-682, doi:10.1038/s41592-022-01488-1 (2022).

7 Jumper, J. *et al.* Highly accurate protein structure prediction with AlphaFold. *Nature* **596**, 583-589, doi:10.1038/s41586-021-03819-2 (2021).

8 Liu, J., Knapp, M., Jo, M., Dill, Z. & Bridwell-Rabb, J. Rieske Oxygenase Catalyzed C-H Bond Functionalization Reactions in Chlorophyll b Biosynthesis. *ACS Cent Sci* **8**, 1393-1403, doi:10.1021/acscentsci.2c00058 (2022).

9 Liu, J. *et al.* Design principles for site-selective hydroxylation by a Rieske oxygenase. *Nat Commun* **13**, 255, doi:10.1038/s41467-021-27822-3 (2022).

10 Martins, B. M., Svetlitchnaia, T. & Dobbek, H. 2-Oxoquinoline 8-monooxygenase oxygenase component: active site modulation by Rieske-[2Fe-2S] center oxidation/reduction. *Structure* **13**, 817-824, doi:10.1016/j.str.2005.03.008 (2005).

11 Kim, J. H. *et al.* Structural and Mechanistic Insights into Caffeine Degradation by the Bacterial N-Demethylase Complex. *J Mol Biol* **431**, 3647-3661, doi:10.1016/j.jmb.2019.08.004 (2019).

12 Quareshy, M. *et al.* Structural basis of carnitine monooxygenase CntA substrate specificity, inhibition, and intersubunit electron transfer. *Journal of Biological Chemistry* **296**, doi:10.1074/jbc.RA120.016019 (2021).

13 Furusawa, Y. *et al.* Crystal structure of the terminal oxygenase component of biphenyl dioxygenase derived from Rhodococcus sp. strain RHA1. *J Mol Biol* **342**, 1041-1052, doi:10.1016/j.jmb.2004.07.062 (2004).

14 Karlsson, A. *et al.* Crystal structure of naphthalene dioxygenase: side-on binding of dioxygen to iron. *Science* **299**, 1039-1042, doi:10.1126/science.1078020 (2003).

15 Mahto, J. K. *et al.* Molecular insights into substrate recognition and catalysis by phthalate dioxygenase from Comamonas testosteroni. *J Biol Chem* **297**, 101416, doi:10.1016/j.jbc.2021.101416 (2021).

16 Friemann, R. *et al.* Structural insight into the dioxygenation of nitroarene compounds: the crystal structure of nitrobenzene dioxygenase. *J Mol Biol* **348**, 1139-1151, doi:10.1016/j.jmb.2005.03.052 (2005).

17 Friemann, R. *et al.* Structures of the multicomponent Rieske non-heme iron toluene 2,3-dioxygenase enzyme system. *Acta Crystallogr D Biol Crystallogr* **65**, 24-33, doi:10.1107/S0907444908036524 (2009).
