# Supplementary material for: Custom tuning of Rieske oxygenase reactivity
Source: Nat Commun. 2023 Sep 20;14:5858. doi: 10.1038/s41467-023-41428-x (PMC10511449; doi:10.1038/s41467-023-41428-x)
Supplement: Supplementary file 3 — Description of Additional Supplementary Files [file 41467_2023_41428_MOESM3_ESM.pdf]

<u>Description of Additional Supplementary Files</u>

Supplementary Data 1: This file contains the protein and DNA sequences for all proteins expressed and purified in this work. This file also contains a list of suppliers and CAS numbers for all of the chemicals used in this work.
